# Design of novel and highly selective SARS-CoV-2 main protease inhibitors

**DOI:** 10.1128/aac.00562-24

**Published:** 2024-09-03

**Authors:** Adi N. R. Poli, Ian Tietjen, Nitesh K. Nandwana, Joel Cassel, Troy E. Messick, Emery T. Register, Frederick Keeney, Rajesh Rajaiah, Atul K. Verma, Kabita Pandey, Arpan Acharya, Siddappa N. Byrareddy, Luis J. Montaner, Joseph M. Salvino

**Affiliations:** 1 Medicinal Chemistry, The Wistar Institute, Philadelphia, Pennsylvania, USA; 2 HIV-1 Program in the Vaccine and Immunotherapy Center, The Wistar Institute, Philadelphia, Pennsylvania, USA; 3 The Wistar Cancer Center Molecular Screening, The Wistar Institute, Philadelphia, Pennsylvania, USA; 4 The Wistar Institute, Philadelphia, Pennsylvania, USA; 5 Department of Pharmacology & Experimental Neuroscience, University of Nebraska Medical Center, Omaha, Nebraska, USA; 6 Molecular and Cellular Oncogenesis (MCO) Program, The Wistar Institute, Philadelphia, Pennsylvania, USA; IrsiCaixa Institut de Recerca de la Sida, Barcelona, Spain

**Keywords:** SARS-CoV-2, protease inhibitor, peptide mimetic, benzoxazepine

## Abstract

We have synthesized a novel and highly selective severe acute respiratory syndrome coronavirus 2 (SARS-CoV-2) main protease peptide mimetic inhibitor mimicking the replicase 1ab recognition sequence -Val-Leu-Gln- and utilizing a cysteine selective acyloxymethyl ketone as the electrophilic warhead to target the active site Cys145. Utilizing a constrained cyclic peptide that locks the conformation between the P3 (Val) and P2 (Leu) residues, we identified a highly selective inhibitor that fills the P2 pocket occupied by the leucine residue sidechain of PF-00835231 and the dimethyl-3-azabicyclo-hexane motif in nirmatrelvir (PF-07321332). This strategy resulted in potent and highly selective Mpro inhibitors without inhibiting essential host cathepsin cysteine or serine proteases. The lead prototype compound 1 (MPro IC_50_ = 230 ± 18 nM) also inhibits the replication of multiple SARS-CoV-2 variants *in vitro*, including SARS-CoV-2 variants of concern, and can synergize at lower concentrations with the viral RNA polymerase inhibitor, remdesivir, to inhibit replication. It also reduces SARS-CoV-2 replication in SARS-CoV-2 Omicron-infected Syrian golden hamsters without obvious toxicities, demonstrating *in vivo* efficacy. This novel lead structure provides the basis for optimization of improved agents targeting evolving SARS-CoV-2 drug resistance that can selectively act on Mpro versus host proteases and are less likely to have off-target effects due to non-specific targeting. Developing inhibitors against the active site of the main protease (Mpro), which is highly conserved across coronaviruses, is expected to impart a higher genetic barrier to evolving SARS-CoV-2 drug resistance. Drugs that selectively inhibit the viral Mpro are less likely to have off-target effects warranting efforts to improve this therapy.

## INTRODUCTION

Severe acute respiratory syndrome coronavirus 2 (SARS-CoV-2) caused a global pandemic in 2020 which continues to inflict substantial morbidity and mortality worldwide. As of February 2024, over 775 million SARS-CoV-2 cases, resulting in over 7.0 million deaths worldwide, were reported to the World Health Organization. Although more than 21 distinct SARS-CoV-2 vaccines are approved globally (1), the virus continues to evolve rapidly to generate variants of concern (VOCs) and VOC subvariants with improved transmission and/or reduced responsiveness to current vaccine measures, particularly after partial vaccination (2–5). VOCs contain mutations in the SARS-CoV-2 spike receptor-binding domain (6), the primary viral regulator of cell entry and the main target of neutralizing antibody activity. These mutations, in turn, drive impaired recognition of the virus by human antibody-mediated immunity (4, 7, 8). Furthermore, poor vaccine accessibility in many parts of the world, combined with widespread vaccine hesitancy in vaccine-accessible regions, increases the risk of sustained SARS-CoV-2 infections and the emergence of variants with vaccine breakthrough potential. These ongoing events all demonstrate a necessity for additional viral countermeasures.

Current licensed antiviral therapies include immunosuppressants, chemotherapy against the viral RNA-dependent RNA polymerase (i.e., remdesivir; molnupiravir) (9–11), and neutralizing antibody infusions during advanced disease, with the latter two depending on access to in-patient infusion resources (12). In addition, Pfizer was recently granted FDA full approval for their oral antiviral treatment Paxlovid (nirmatrelvir-ritonavir combination), which inhibits the SARS-CoV-2 chymotrypsin-like cysteine or main protease (3CLpro or Mpro). The Mpro inhibitor, nirmatrelvir, is co-administered with ritonavir, a CYP P450 3A4 inhibitor, to enhance metabolic stability (13) and thus has the potential for both undesired drug-drug interactions and incomplete viral inhibition (14). Thus, additional antiviral chemotherapies with improved drug-like properties such as improved metabolic stability, avoidance of CYP P450 co-inhibitors, and minimal drug-drug interactions remain needed for next-generation first-line treatments.

SARS-CoV-2 contains two overlapping open reading frames at the end of the 5´ terminal, which encodes for two essential polypeptides called pp1a and pp1ab. These polypeptides produce most of the proteins involved in the replicase-transcriptase complex, the large majority of which are processed by Mpro at ≥11 viral cleavage sites (15, 16). Thus, Mpro is responsible for the release of the mature non-structural proteins Nsp5–16, including Mpro (Nsp5) itself. These are required for further viral replication and transmission (15) and loss of Mpro activity by therapeutic targeting blocks progression of SARS-CoV-2 replication. Moreover, as the active sites of Mpro are highly conserved across coronaviruses (17), Mpro inhibitors may impart a higher genetic barrier to evolving SARS-CoV-2 drug resistance when administrated either alone or in combination with agents that target other aspects of viral replication. Also, since new coronaviruses are expected to emerge, targeting an essential but structurally conserved enzyme may provide additional therapeutic leads for future coronavirus outbreaks. Importantly, drugs that selectively act on Mpro versus host proteases are less likely to have off-target effects due to non-specific targeting. As no known human protease shares the same substrate specificity as Mpro (18, 19), developing inhibitors with high Mpro selectivity appears feasible. Selectivity for current Mpro inhibitors under development is a crucial consideration (13, 20).

We report a novel series of SARS-CoV-2 Mpro inhibitors that use a conformationally restricted peptidomimetic scaffold (21) to mimic the bioactive protease-bound conformation (22). This strategy results in potent and highly selective Mpro inhibitors without inhibiting essential host cathepsin cysteine proteases or serine proteases. The lead compound 1 also inhibits replication of multiple SARS-CoV-2 variants *in vitro,* including SARS-CoV-2 VOC, and can synergize at lower concentrations with the viral RNA polymerase inhibitor remdesivir to inhibit replication. Finally, it also reduces SARS-CoV-2 replication in hamsters without obvious toxicities, demonstrating *in vivo* efficacy for this class of compounds.

## RESULTS

### Design of a P3-P2 peptide mimetic MPro inhibitor

**Fig 1:**
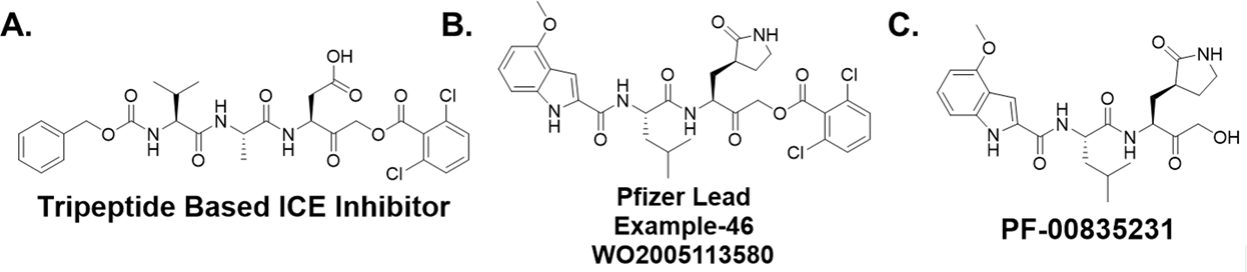
Design of cysteine protease inhibitors. (A) Tripeptide-based interleukin-1β converting enzyme inhibitor. (B) Pfizer coronavirus main protease lead compound reported in WO2005113580. (C) The hydroxymethyl ketone Pfizer lead compound, PF-00835231.

We took advantage of our previous experience in protease inhibitor development of interleukin-1β converting enzyme (ICE) inhibitors (21, 23, 24) to design Mpro inhibitors. This approach utilized an acyloxymethyl ketone electrophilic warhead (Fig. 1A). These electrophilic warheads were designed as clinically useful halomethyl ketone analogs to specifically react with the thiolate of an active site cysteine (24, 25). Notably, Pfizer utilized a similar approach resulting in their acyloxymethyl ketone lead compound reported in their 2005 patent application (WO2005113580) describing example-46 (Fig. 1B), which is similar to the Pfizer hydroxymethyl ketone PF-00835231 (Fig. 1C). The similarity between the ICE inhibitors and the Mpro inhibitors inspired the start of our work, before we were aware of the structure of nirmatrelvir.

**Fig 2:**
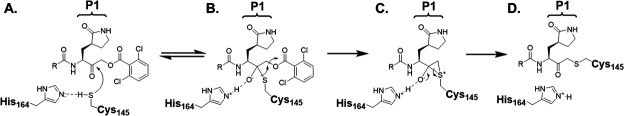
The mechanism of irreversible inhibition of cysteine proteases by acyloxymethyl ketones. (A) The active site His164 acts as a base to enhance the nucleophilicity of the Cys145 thiol. (B) Nucleophilic attack of the Cys145 thiol to the ketone carbonyl generates a reversible thiohemiketal complex. (C) Attack by the thiol leads to the thiiranium intermediate species, which collapses to form the irreversible covalent thiol adduct (D).

The acyloxymethyl ketone moiety is amenable to incorporation of various leaving groups, allowing for control of selectivity and reactivity toward cysteine proteases (25). Importantly, this class of inhibitors is selective toward cysteine proteases versus serine proteases, and is relatively inert toward bio-nucleophiles such as glutathione, making them suitable for *in vivo* studies (25). This class of inhibitor is not a pro-drug which would require hydrolysis of the ester linkage for biological activity. The mechanism (Fig. 2) of irreversible inhibition of cysteine proteases by acyloxymethyl ketones involves formation of a reversible E-I complex (Fig. 2A) where the active site residues His164 and Cys145 exist as a thiolate/imidazolium ion pair, and where the His residue polarizes the ketone carbonyl to initiate a thiolate attack. This results in a thiohemiketal complex (Fig. 2B), followed by rearrangement to a thiiranium species (Fig. 2C), which in turn collapses to form the covalent thiolate adduct (Fig. 2D).

**Fig 3:**
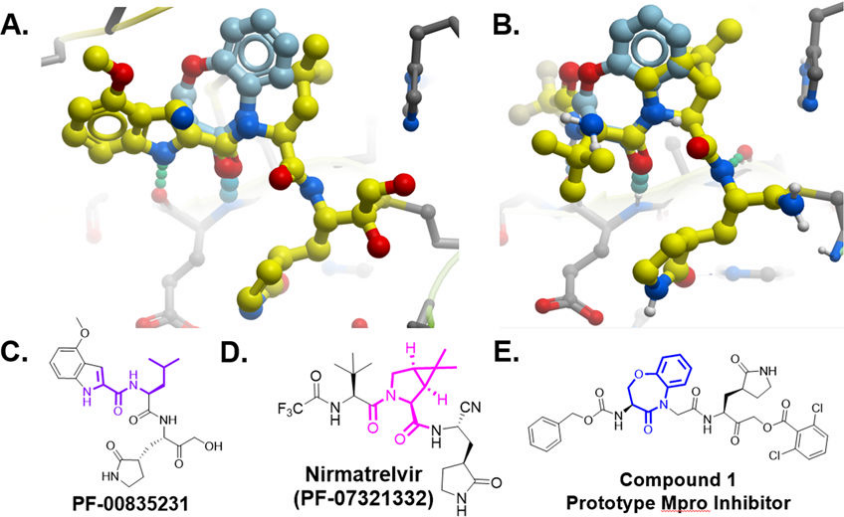
Manual overlap of the benzoxepine scaffold on Mpro inhibitor structures. (A) Manual overlay of the benzoxazepine scaffold (light blue) to PF-00835231 using PDB 6xhl showing SARS-CoV-2 in complex with PF-00835231, aligning the carbonyl H-bond showing the potential to fill the P2 Leu pocket. (B) Manual overlay of the benzoxazepine scaffold (light blue) to PF-07321332 using PDB 7si9 showing SARS-CoV-2 in complex with PF-07321332, aligning the carbonyl H-bond showing the potential to fill the P2 Leu pocket. (C) The 2-D structure of PF-00835231 is highlighted to show regions of overlap. (D) The 2-D structure of PF-07321332, nirmatrelvir, is highlighted to show regions of overlap. (E) 2-D structure of compound 1, the prototype Mpro inhibitor, is highlighted to show the benzoxazepine motif. Modeled structures were obtained using Mol Soft, LLC.

Thus, we focused on identifying a peptidomimetic to replace the Val-Leu (P3-P2) di-peptide portion of the -Val-Leu-Gln- recognition segment (26, 27). Using the numerous Mpro substrate or inhibitor-bound X-ray crystal structures available for guidance, we modeled various conformationally constrained peptide mimetics (21) which best accommodated the pockets occupied by the P3-P2 substituents but also retained key hydrogen bonds. We also retained the glutamine mimetic in the P1 position that was used in PF-00835231 (Fig. 1C). We hypothesized that a highly constrained peptide mimetic that best resembled the bioactive conformation (22) of the peptide-enzyme complex would provide exquisite selectivity. A benzoxazepine acetic acid (21, 28, 29) constrained P3-P2 mimetic, previously used to synthesize conformationally restricted angiotensin converting enzyme (ACE) and ICE inhibitors, was prioritized for synthesis since this appeared to closely represent the bioactive conformation of peptide or inhibitor-bound Mpro antagonists (30). We performed manual overlays of the benzoxazepine motif onto the Mpro inhibitors based on their X-ray crystal structures to suggest how this scaffold could lock the conformation between the P3 and P2 residues (Fig. 3), and can potentially fill the P2 lipophilic pocket with the aryl ring. Based on the structure overlays to the inhibitor complexes, this scaffold was predicted to provide a favorable conformationally locked mimic of the -Val (P3)-Leu (P2)- recognition segment.

### Synthesis of Mpro inhibitors

**Fig 4:**
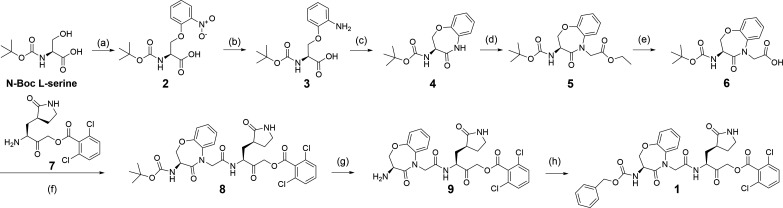
Synthesis of compound 1. Reagents and conditions: (a) NaH, DMF, 1-fluoro-2-nitrobenzene, 0°C–40°C, 2 h. (b) 10% Pd/C, H_2_(g), EtOH, room temperature, 16 h. (c) 50% T3P in CH_2_Cl_2_, DIPEA, −20°C to 0°C, 1 h. (d) LiHMDS, THF, ethyl 2-bromoacetate, −78°C to room temperature, 12 h. (e) NaOH, THF: MeOH: H_2_O, 0°C to room temperature, 12 h. (f) 50% T3P in CH_2_Cl_2_, DIPEA, −0°C to room temperature, 1 h. (S)-3-amino-2-oxo-4-((S)-2-oxopyrrolidin-3-yl)butyl 2,6-dichloro benzoate. (g) 20% TFA in CH_2_Cl_2_, 0°C to room temperature, 2 h. (h) Cbz chloride, Et_3_N, CH_2_Cl_2_, 0°C to room temperature, 24 h.

The prototype peptidomimetic Mpro inhibitor, 1, was synthesized using the following method shown in Fig. 4. The nucleophilic oxygen atom of N-Boc L-serine reacted with 2-fluoro-nitrobenzene under base-catalyzed conditions to form the aryl ether, 2, which was then treated using Pd-catalyzed reduction conditions in the presence of H_2_ gas to reduce the aryl nitro group to the aniline, 3. Compound 3 was then reacted under propane phosphonic acid anhydride (T3P) amide bond coupling-cyclization conditions to provide the P3-mimetic, 4. Compound 4 was then treated with the strong base, LiHMDS, followed by ethyl bromo-acetate under N-alkylation conditions, to provide the N-Boc protected P3-P2-mimetic as the ethyl ester, 5. Compound 5 was then treated under aqueous basic conditions to hydrolyze the ethyl ester to form the free carboxylic acid, 6, which was reacted with the acyloxymethyl ketone P1 glutamine mimetic, 7, in the presence of the amide bond coupling reagent T3P. The coupled product, 8, was reacted under acidic conditions to remove the N-Boc protecting group to provide the primary amine, 9, which in turn was reacted with Cbz-chloride in the presence of an organic base to form the N-Cbz derivative, to provide the fully elaborated peptide mimetic 1 (Fig. 4).

**Fig 5:**
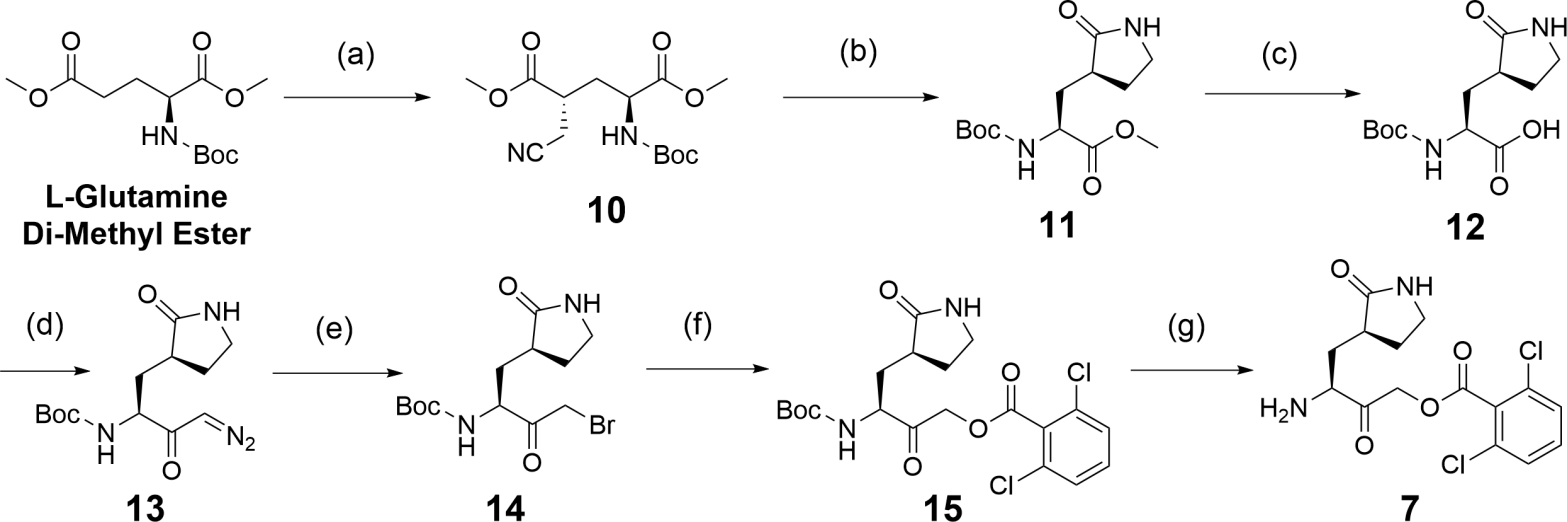
Synthesis of intermediate 7. Reagents and conditions: (a) 2-bromoacetonitrile, Lithium bis(trimethylsilyl)amide solution 1.0 M in THF, THF, −78°C to room temperature, 2 h. (b) (i) H_2_(g), Pt_2_O, MeOH, room temperature, 24 h. (ii) MeOH, room temperature, 24 h. (c) 20% aq NaOH in MeOH: THF: H_2_O (5:5:1), rt, 4 h. (d) Isobutyl chloroformate, THF, Et_3_N, −30°C to 10°C, 1 h then CH_2_N_2_ in Et_2_O, 0°C to room temperature, 16 h. (e) 33% HBr in H_2_O, THF, −20°C. (f) Cesium fluoride, 2,6-dichlorobenzoic acid, THF. (g) 20% TFA in CH_2_Cl_2_, 0°C to room temperature, 2 h.

**Fig 6:**
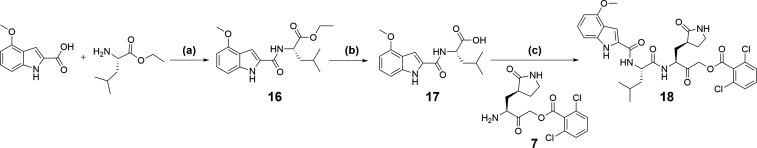
Synthesis of Pfizer compound 18. Reagents and conditions: (a) 50% T3P in DMF, DIPEA, 0°C to room temperature, 1 h. (b) LiOH in MeOH: THF: H_2_O (5:5:1), rt, 5 h. (c) 50% T3P in DMF, DIPEA, 0°C to room temperature, 1 h.

The synthesis of the important acyloxymethyl ketone P1 glutamine mimetic, Compound 7, was accomplished using the reported synthesis starting from N-Boc L-glutamine di-methyl ester as shown (Fig. 5) (21, 23, 31, 32). We synthesized the Pfizer reference compound 18 reported in in their 2005 patent application (WO2005113580; example-46; Fig. 1B) (33), analogous to PF-00835231 as shown (Fig. 6), and purchased GC-376 to use as control compounds for selectivity comparisons to our prototype inhibitor, 1. Synthesis of the amides 19 and 20 was accomplished starting from intermediate 8 (Fig. 4), which was deprotected under mild acidic conditions to provide the primary amine, 9, and then coupled with the corresponding carboxylic acids (Table 1). We also synthesized an analog of 1, compound 21, which replaced the di-chloro-benzoate leaving group with a 1-(4-fluorophenyl)-3-(trifluoromethyl)-1H-pyrazol-5-ol leaving group, that was successful in the design of interleukin-1β converting enzyme inhibitors (23) (Fig. S1).

**TABLE 1:** IC_50_ values^*a*^ of 1, analogs, and controls for enzyme activity against cysteine and serine proteases

Structure	Compound ID	MPro IC_50_, nM	CatB IC_50_, nM	CatL IC_50_, nM	Thrombin IC_50_, nM
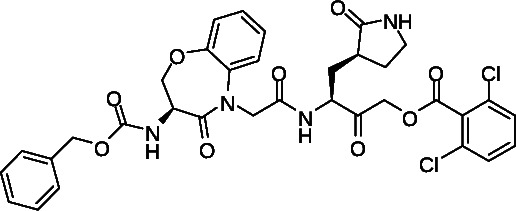	1	230 ± 18	18,000 ± 9,100	>32,000	>32,000
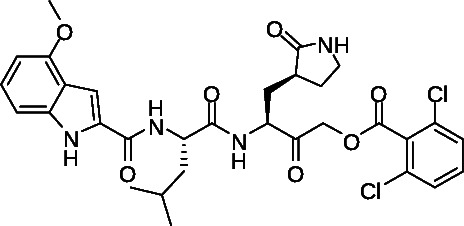	18	11 ± 0.7	24 ± 7.5	1.8	>10,000
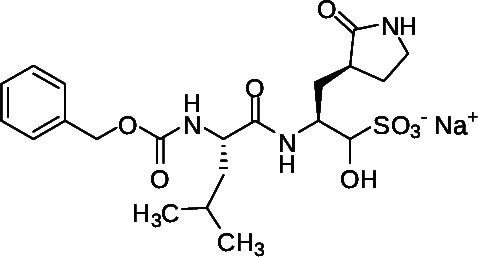	GC-376	18 ± 1.5	37 ± 15	0.05	>10,000
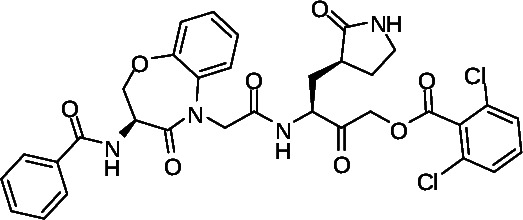	19	620 ± 48	13,000 ± 3,600	>32,000	> 32,000
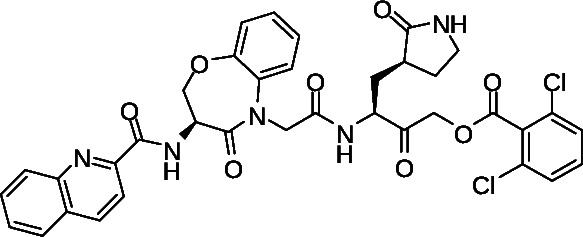	20	580 ± 40	1,800 ± 260	23,058	> 32,000
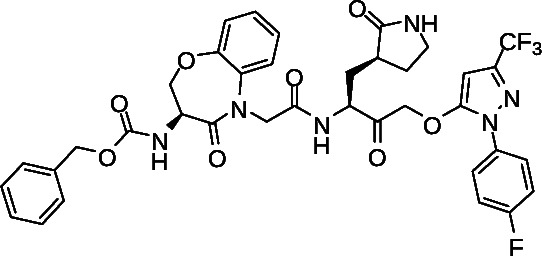	21	3,100 ± 770	27,000 ± 11,000	>32,000	>32,000

^*a*^ Enzyme assay. Values were calculated from at least 10-point dose-response curves performed in duplicate.

### Biological evaluation

To assess biological activity, we first developed a continuous, fluorescence-based Mpro enzymatic assay that monitors the cleavage of the fluorescently quenched substrate DABCYL-Lys-HCoV-SARS replicase polyprotein 1ab (3235–3246)-Glu-EDANS (34, 35). Upon cleavage of the substrate, an increase in fluorescence of EDANS is observed at 355/490 nm, which in turn can be inhibited with co-incubation with Mpro inhibitors. Limits of protein and time linearity, substrate *K*_*m*_ and *V*_max_, tolerance of 1% dimethyl sulfoxide (DMSO), reproducibility of screening, and titrations of reference compounds were conducted for assay validation (see Materials and Methods). The *K*_*m*_ for the substrate was observed to be 30 µM. At 6 µM substrate, the reaction was linear for 30 min with up to 100 nM Mpro. Based on these observations, we chose 5 µM substrate and 50 nM Mpro concentrations for compound assessment. We also tested the effect of 1% DMSO on Mpro activity and found no adverse effects on the assay. However, compounds with high fluorescence backgrounds interfere in the assay; therefore, background fluorescence was tested separately (Z’ score = 0.83). GC-376 was tested as a positive control and was shown to have a half-maximal inhibitory concentration (IC_50_) of 18 ± 1.5 nM, consistent with reported values (Table 1) (34, 35). Using this assay, we then assessed our inhibitors and confirmed that 1 exhibited a dose-dependent inhibition of Mpro with an IC_50_ of 230 ± 18 nM (Table 1). While derivatives 19 and 20 have comparable efficacies (IC_50_ = 620 ± 48 and 580 ± 40 nM, respectively), compound 21 was the weakest, with more than 10-fold less activity compared to 1. Notably, all derivatives exhibited at least 20-fold lower activities than control compound 18 (Pfizer lead example −46; Fig. 1) (IC_50_ = 11 ± 0.7 nM; Table 1). Interestingly, 1 and derivatives required a 1- to 2-h incubation time to achieve maximum inhibition, suggesting that the highly constrained conformation required longer incubation times for binding to the substrate pocket in contrast to 18 or GC-376 which achieved maximum inhibition within 10 min (36). Moreover, compound 18 also potently inhibited the enzymatic activity of cysteine proteases cathepsin B and L (IC_50_ = 24 ± 7.5 and 1.8 ± 0.27 nM, respectively). Importantly, no substantial inhibition of these proteases was observed by 1 (IC_50_ >15 µM; Table 1), confirming Mpro selectivity for our prototype inhibitor. We also observed no activity against the serine protease, thrombin (IC_50_ >32 µM; Table 1). These data strongly indicate high selectivity of 1 for Mpro, even though the prototype, 1, is ~20-fold less potent than the Pfizer lead, compound 18. As these host proteases are essential for processing host peptides (18) into their mature forms, our prototype compound 1 and derivatives are suggested to have a more attractive biological activity profile since they would have less effect on host protease activities, host protease-mediated cellular homeostasis, and overall safety.

**Fig 7:**
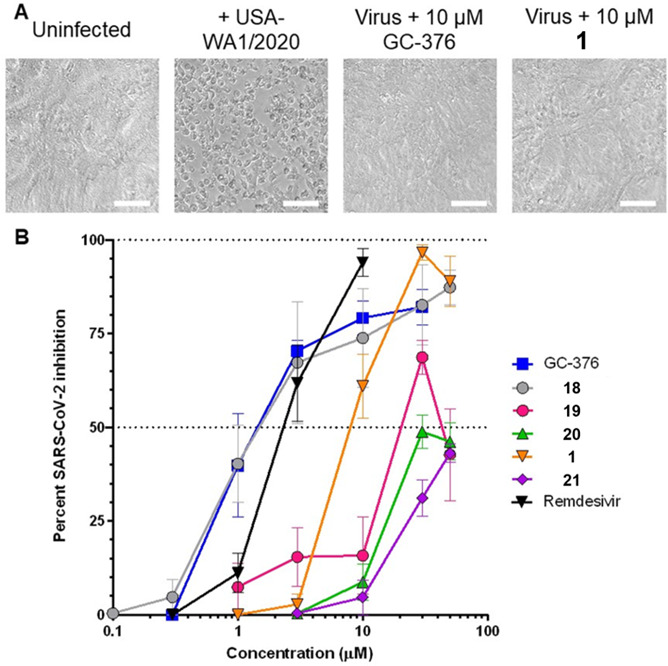
Effects of compounds on CPE in Vero-E6 cells following SARS-CoV-2 infection. (A) Representative brightfield images of uninfected and SARS-CoV-2 USA-WA1/2020 variant-infected Vero-E6 cells following 4-day incubation in the absence or presence of representative compounds. Scale bars = 100 µm. (B) Dose-response curves of Mpro inhibitors and remdesivir on viral replication in Vero-E6 cells after 4-day infection. Data are presented as percent virus inhibition, with 0% denoting the viability of infected cells without drug and 100% denoting the viability of uninfected cells.

To confirm cellular antiviral activity, we used a cytopathic effect (CPE)-based assay with infectious SARS-CoV-2 in Vero-E6 cells as described previously (35). Briefly, Vero-E6 cells were treated with compounds for 2 h in threefold replicates in 96-well format before infection with 150× median tissue culture infectious dose (TCID_50_) of SARS-CoV-2 (USA-WA1/2020 variant). Cells were then incubated for 4 days, at which point, cell viability was assessed using a resazurin stain before fixation with paraformaldehyde. Using this assay, we observed widespread CPE in cells following infection for 4 days corresponding to an average of 59.7 ± 7.4% reduced viability compared to uninfected cells (Fig. 7A). This loss of viability, in turn, was restored with dose-dependence using control SARS-CoV-2 inhibitors like GC-376 and compound 18, which respectively had calculated half-maximal effective concentrations (EC_50_) of 1.8 ± 0.4 and 3.4 ± 1.4 µM (Fig. 7B; Table 2). We also observed comparable activity with the nucleoside analog remdesivir (EC_50_ = 2.7 ± 0.5 µM; Fig. 7B; Table 2) (37), and observed good inhibition with all test compounds. For example, we obtained an EC_50_ of 7.5 ± 2.0 µM for our prototype compound 1 (Fig. 7B; Table 2), which is only 4.1-fold and 2.2-fold reduced in activity compared to GC-376 and 18, respectively (Fig. 7B; Table 2). No cytotoxicity was observed with up to 100 µM of any compound, as measured by resazurin staining following 4-day treatment in uninfected Vero-E6 cells.

**TABLE 2:** EC_50_ values of 1, analogs, and controls in viral CPE assays

EC_50_ (µM)	GC-376	18	19	20	1	21	Remdesivir
USA-WA1/2020	1.8 ± 0.4	3.4 ± 1.4	25.0 ± 5.0	44.5 ± 7.4	7.5 ± 2.0	73.5 ± 12.4	2.7 ± 0.5

**Fig 8:**
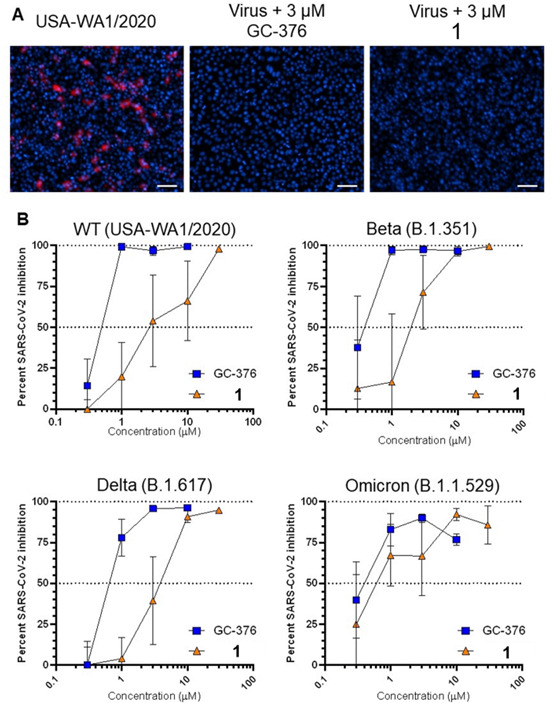
Effects of compounds on viral nucleocapsid protein expression in Vero-E6 cells infected with SARS-CoV-2 VOC. (A) Respective immunostaining images of Vero-E6 cells infected with USA-WA1/2020 virus following 2-day incubation in the absence or presence of compounds. Red denotes viral nucleocapsid protein expression, and blue denotes cell nuclei. Scale bar, 100 µm. (B) Dose-response curves of GC-376 and 1 on viral nucleocapsid expression in Vero-E6 cells after 48-h infection with SARS-CoV-2 VOC, as measured by high-content imaging. Data are presented as percent virus inhibition relative to infected cells without drug.

We next tested the ability of prototype 1 and control GC-376 to inhibit virus replication due to wild-type and SARS-CoV-2 VOC including Beta (B.1.351), Delta (B.1.617), and Omicron (B.1.1.529) using a previously described high-content imaging assay approach (Fig. 8A) (35). Briefly, Vero-E6 cells were treated with compounds for 2 h in threefold replicates in 384-well format before infection with 50× TCID of virus. After 48 h, cells were fixed and immunostained for cellular SARS-CoV-2 nucleocapsid expression. Cell nuclei were counterstained with Hoechst, and high-content imaging was used to count total live and infected cells across each well. This approach detected widespread nucleocapsid-positive (i.e., infected) cells inhibited by both GC-376 and 1 (Fig. 8A). Both compounds also maintained antiviral activities similar to those observed in cells infected with the USA-WA1/2020 variant (Fig. 8B; Table 3). For example, GC-376 inhibited Beta, Delta, and Omicron variants in this assay with EC_50_ of 0.3 ± 0.5, 0.6 ± 0.2, and 0.4 ± 0.5 µM, respectively, compared to 0.3 ± 0.5 µM against the initial USA-WA1/2020 variant (Fig. 8B; Table 3). Similarly, 1 exhibited EC_50_ of 3.1 ± 11.0, 3.0 ± 2.4, and 0.9 ± 0.9 µM against Beta, Delta, and Omicron variants, compared to 2.1 ± 2.1 µM vs USA-WA1/2020 (Fig. 8B; Table 3).

**TABLE 3:** EC_50_ values of GC-376 and 1 in viral high-content imaging assays

EC_50_ (µM)	GC-376	1
WT (WA1/2020)	0.3 ± 0.5	2.1 ± 2.1
Beta (B.1.351)	0.3 ± 0.5	3.1 ± 11.0
Delta (B.1.617)	0.6 ± 2.2	3.0 ± 2.4
Omicron (B.1.1.529)	0.4 ± 0.5	0.9 ± 0.9

### Combinations support synergistic effects

**Fig 9:**
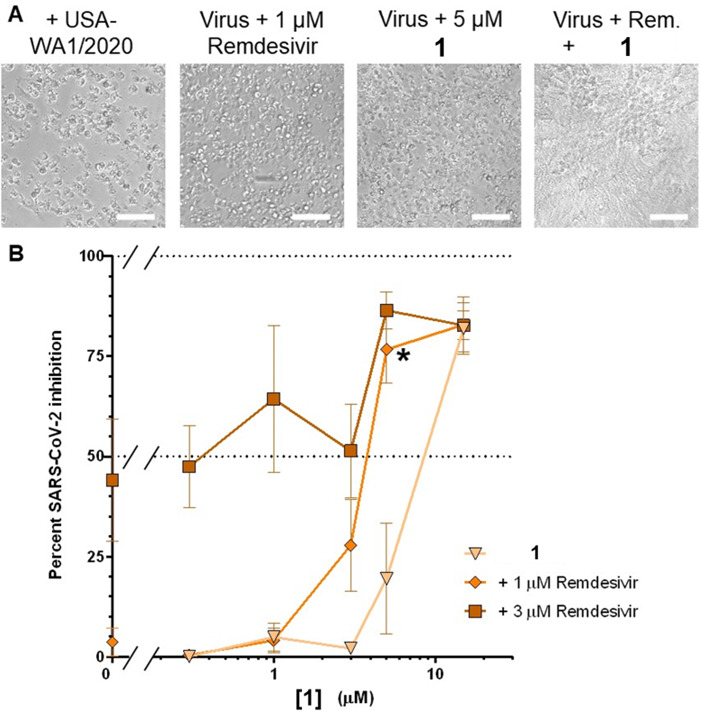
Effects of 1 in combination with remdesivir in Vero-E6 cells following 4-day infection with SARS-CoV-2 (USA-WA1/2020 variant). Assays were performed as described in Fig. 7.** (A)** Representative brightfield images of infected cells following 4-day incubation in the absence or presence of remdesivir and/or 1. Scale bars = 100 µm. (**B)** Dose-response curves of compound 1 (x-axis) in the presence of 1 or 3 µM remdesivir. Treatment with 5 µM of 1 plus 1 µM of remdesivir significantly inhibits SARS-CoV-2-induced CPE relative (asterisk), as measured using the Bliss independence model, when compared to cells treated with 1 alone (triangles) or remdesivir alone (left-most values). Data are presented as percent virus inhibition, with 0% denoting the viability of infected cells without drug and 100% denoting the viability of uninfected cells.

Mpro processes the viral polypeptide pp1ab which encodes for essential non-structural proteins important in viral replication and transcription, including the RNA-dependent RNA polymerase (15), which is the viral target of remdesivir. Therefore, we used the CPE assay to evaluate 1 at sub-optimal antiviral doses (0.1 to 5 µM) in combination with sub-optimal antiviral doses of remdesivir (0.1 to 3 µM) in USA-WA1/2020 variant SARS-CoV-2-infected Vero-E6 cells to evaluate potential synergistic effects when applied in combination (Fig. 9). Notably, enhanced CPE inhibition, as measured by resazurin stain after 96 h of infection, was observed when either 3 or 5 µM of 1 was combined with 1 or 3 µM of remdesivir, none of which were effective at inhibiting CPE alone (Fig. 9A). For example, when assessed across three independent experiments, single treatments of either 5 µM of **1** or 1 µM of remdesivir inhibited CPE in infected cells by only an average of 19.5 ± 13.7% and 3.6 ± 3.1%, respectively. In contrast, co-incubation with both 5 µM of 1 and 1 µM of remdesivir inhibited SARS-CoV-2 replication by 76.7 ± 8.4% (Fig. 9B; asterisk), resulting in a 3.3-fold increased inhibition relative to what would be expected if 1 and remdesivir acted by strictly additive effects (i.e., 23.1% inhibition). This level of synergism was statistically significant (*P* = 0.017; Student’s paired *t*-test) as measured by the Bliss independence model of evaluating drug combination activities (38). Similarly, enhancement was also observed when 1 µM remdesivir was paired with 1 µM 1 or when 3 µM of remdesivir and 1 were paired (Fig. 9B), although these did not reach statistical significance. These results indicate that low doses of 1 and remdesivir, which are ineffective on their own, can combine synergistically to inhibit SARS-CoV-2 replication, which may further potentially minimize potential off-target effects of individual compounds when applied as monotherapy. In contrast, no apparent changes in inhibition were observed when remdesivir was added to concentrations of 1 below 3 µM (Fig. 9B). Addition of less than 1 µM of remdesivir also did not improve the antiviral activity of 1 at any concentration (data not shown).

### Absorption, distribution, metabolism, and excretion (ADME)/pharmacokinetic (PK) evaluation

**Fig 10:**
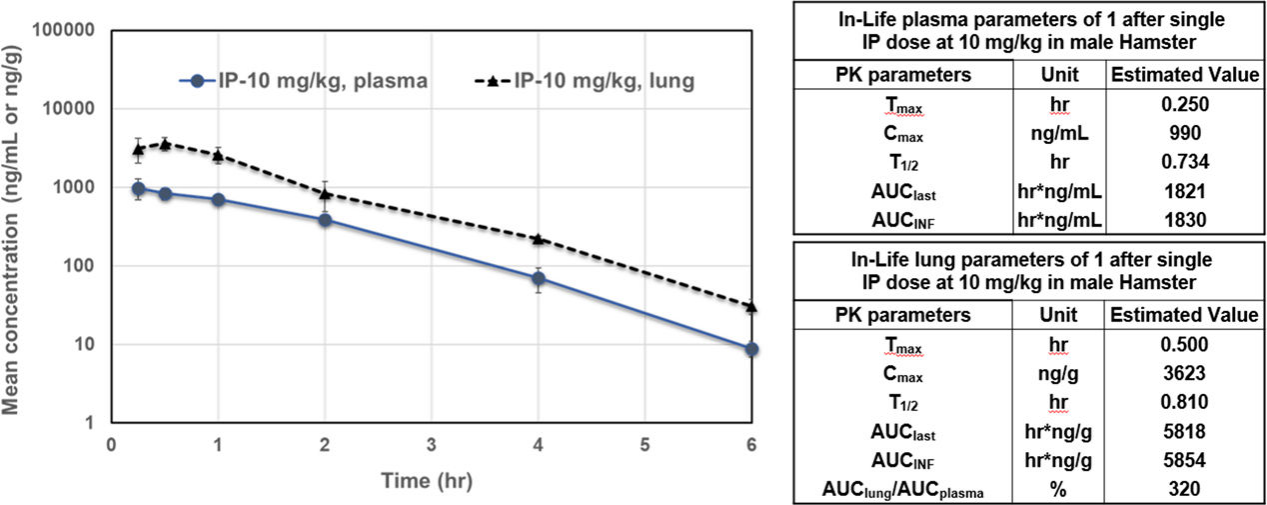
Mouse pharmacokinetics of 1. Mean plasma and lung concentration-time profiles of compound 1 after single i.p. dose at 10 mg/kg in male hamster (*N* = 3/time point). In-life parameters of compound 1 are provided for plasma and lung.

Compounds 1, 9, 18, 19, and 20 were next evaluated for metabolic stability (ChemPartner) by incubation in mouse liver microsomes. Unfortunately, all these analogs show poor stability in this assay with a half-life (T_1/2_) of less than 2 min. The electrophilic ketone is suspected to be the key metabolic liability. Compound 1 was also evaluated in a male hamster pharmacokinetic study (ChemPartner) to determine plasma and lung concentration levels over time. Compound 1 was administered as a single dose at 10 mg/kg via intraperitoneal (i.p.) injection formulated in 10% DMSO/10% Solutol HS15/phosphate buffered saline (PBS) at 2 mg/mL (Fig. 10). There were no abnormal clinical symptoms observed during the in-life phase. Interestingly, the concentration of compound 1 in the lung was 320% higher than plasma (AUC_lung_/AUC_plasma_), achieving a concentration of about 8 µM at 10 mg/kg, suggesting that doses 5× more, or at least 50 mg/kg, may provide compound levels close to those showing efficacy in the CPE assay. We used 100 mg/kg in this first study since it was tolerated and to increase our chances of showing efficacy with this early-stage compound with modest pharmacokinetic exposure.

### Compound **1** provides partial protection of SARS-CoV-2 infections in hamsters

**Fig 11:**
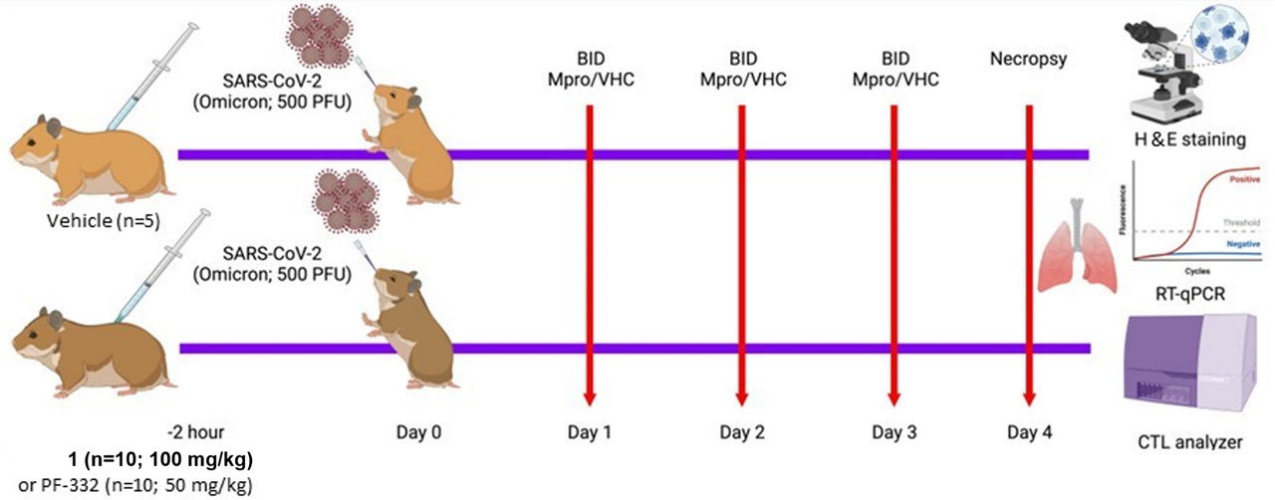
Experimental scheme outlining Mpro inhibitor treatment in SGH.

**Fig 12:**
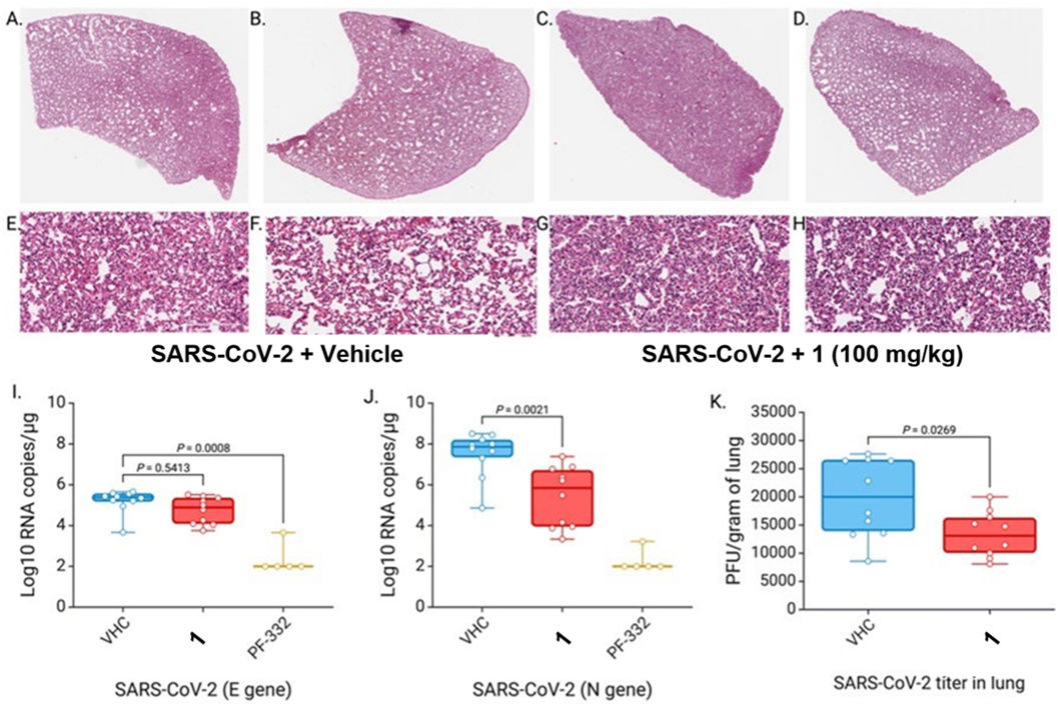
Compound 1 reduces SARS-CoV-2 viral load and live virus titer in SARS-CoV-2 Omicron-infected SGH. (A–H) Representative H&E staining of lung tissue of SARS-CoV-2 Omicron-infected SGH treated with vehicle (**A–B, E–F**) or compound 1 (**C–D, G–H**). Images are from two representative SGHs per condition, with magnification at 1× (**A–D**) and 20× (**E–H**). **(I**) RT-qPCR of genomic RNA (E gene) from lung tissue from vehicle (VHC), 1, and PF-332-treated SGH. (**J**) RT-qPCR of sub-genomic RNA (N gene) from VHC, 1, and PF-332-treated SGH. (**K**) SARS-CoV-2 viral titer in lung tissue of VHC and 1-treated SGH.

To evaluate the efficacy of **1** *in vivo*, we used the experimental schema shown in Fig. 11. Briefly, 10 Syrian golden hamsters (SGHs) were first treated prophylactically with an i.p. injection of compound 1 (100 mg/kg), positive control Mpro inhibitor PF-07321332 (nirmatrelvir; PF-332; 50 mg/kg), or vehicle control. After 2 h, SGHs were infected intranasally with 500 plaque forming units (PFU) of SARS-CoV-2 (B.1.1.529/Omicron). Additional i.p. injections of 1 (100 mg/kg), PF-332 (50 mg/kg), or vehicle control were performed twice daily for 4 days, after which SGHs were euthanized. Hamsters observed no obvious toxicities or behavioral abnormalities during dosing. The gross pathology of SGH lung tissue was evaluated by hematoxylin and eosin (H&E) staining. Lung tissue was also assessed for SARS-CoV-2 genomic RNA, as measured by real-time quantitative PCR (RT-qPCR), and for infectious viral titer. When assessed by H&E staining, we did not observe any significant difference in the histopathology scores of 1-treated SGH compared to vehicle-treated SGH (Fig. 12). H&E images from two representative animals treated with vehicle control and Mpro-treated animals are shown (Fig. 12A through H). However, we did observe a modest but clear reduction of SARS-CoV-2 genomic RNA from lung tissue of SGH treated with 1 when compared to controls, where a mean 0.5 log reduction in genomic RNA (E gene; *P* = 0.54; Fig. 12I) and a mean 2.0 log reduction in SARS-CoV-2 sub-genomic RNA (N gene; *P* = 0.002; Fig. 12J) were observed. These results indicate that compound 1 can limit SARS-CoV-2 replication in the lungs of infected SGH. In contrast, SGH treated with nirmatrelvir (PF-332) positive control exhibited 3.0 log and 5.2**-**fold reductions in E genomic RNA and N sub-genomic RNA, respectively (Fig. 12I and J; *P* values = 8 * 10^−4^ and 2 * 10^−3^, respectively; Fig. 12I and J). We also observed a statistically significant 32.6% reduction in SARS-CoV-2 viral titer from lung tissue from 1-treated SGH compared to controls (*P* = 0.03; Fig. 12K). These data indicate that 1 can reduce both viral load and live virus titer in the lungs of Omicron variant-infected SGH, although not to the level of positive control nirmatrelvir (PF-332). Presumably, optimized derivatives of 1 would have more efficacy while retaining selectivity against host proteases, *in vivo* stability, and no significant toxicities.

### Conclusion

In summary, we have identified a highly selective prototype Mpro inhibitor, compound 1, through the use of a conformationally restricted peptidomimetic. The prototype, compound 1, is highly selective over host cysteine and serine proteases compared to the Pfizer lead, compound 18 and GC-376. Nirmatrelvir was not evaluated in our selectivity assay due to compound availability when we started the study. However, it was subsequently made commercially available and was then used as a positive control for *in vivo* studies (13). Compound 1 also inhibits *in vitro* SARS-CoV-2 replication, including across three VOC, namely Beta, Delta, and Omicron, and can synergize with remdesivir to impede the virus at sub-optimal monotherapy concentrations. Compound 1 also limited SARS-CoV-2 production in the lungs of Omicron variant-infected hamsters, demonstrating proof of concept for further optimized compounds for *in vivo* use. We initially used a high dose of 100 mg/kg (i.p.) since it was predicted to provide *in vivo* exposures similar to effective doses in the CPE assay. We realize that compound 1 has modest pharmacokinetic properties, most likely due to its modest metabolic stability. This is probably due to the electrophilic acyloxymethyl ketone motif. Thus, as we optimize the series, we will focus on incorporation of electrophilic traps for the catalytic cysteine that improve on metabolic stability. Additionally, we will attempt to develop a competitive inhibitor so we can remove this suspected metabolic liability, as was done for S-217622 (39). The hamster PK study was done by i.p. administration (10 mg/kg with a dosing volume of 5 mL/kg) and a formulation of 10% DMSO, 10% Solutol HS 15, plus 80% PBS. For the *in vivo* efficacy study, we used similar dosing volumes but reduced the percentage of DMSO, since we were concerned it would have an effect, and compared it to a vehicle control. We chose to administer nirmatrelvir by the i.p. route of administration versus oral administration to save on drug costs and to avoid the complications due to oral dosing, and we found that the 50 mg/kg dose worked effectively as a positive control to reduce SARS-CoV-2 viral load and live virus titer in SARS-CoV-2 Omicron-infected SGH. As the active site of Mpro is highly conserved across coronaviruses, targeting this protease is also anticipated to provide a high barrier to resistance when administered either as a monotherapy or in combination with other SARS-CoV-2 antivirals, although this hypothesis requires further exploration. Our focus is to develop a highly selective Mpro inhibitor that does not have off-target effects on host proteases. The majority of SARS-CoV-2 Mpro inhibitors are peptidomimetic covalent inhibitors which utilize an electrophilic warhead to target the catalytic cysteine residue to provide potent Mpro inhibition, but they can also lead to off-target effects at other cysteine proteases, such as cathepsin L and others. However, it may be beneficial to inhibit the lysosomal cysteine protease, cathepsin L, or calpain under pathogenic conditions, and this has been investigated as a therapeutic approach (40, 41). In some instances, targeting host proteases not essential for normal functioning of healthy cells may be tolerated, but the potential risks and side effects due to prolonged inhibition of proteases involved in the turn-over of key cellular proteins should be carefully evaluated (42). Therefore, optimization of the lead compound 1 is expected to provide highly selective candidates with improved potency and suitable pharmacokinetic profiles for advancement as potential anti-coronaviral therapy.

## MATERIALS AND METHODS

### Compound synthesis

Detailed synthetic procedures and spectral characterization are provided in the supplemental material.

### *In vitro* biological evaluation studies

#### Cells, viruses, and reagents

Vero-E6 cells were obtained from the American Tissue Culture Collection and cultured in Dulbecco’s Modified Eagle Medium with 4.5 g/L glucose and L-glutamine (Gibco, Gaithersburg, MD), 10% fetal bovine serum (Gemini Bio Products, West Sacramento, CA, USA), 100 U of penicillin/mL, and 100 µg of streptomycin/mL (Sigma Aldrich, St. Louis, MO) (D10+ medium) at 37°C and 5% CO_2_. The following reagent was deposited by the Centers for Disease Control and Prevention and obtained through BEI Resources, NIAID, NIH: SARS-Related Coronavirus 2, Isolate USA-WA1/2020, NR-52281. The following reagents were obtained through BEI Resources, NIAID, NIH: SARS-Related Coronavirus 2, Isolate hCoV-19/England/204820464/2020, NR-54000, contributed by Bassam Hallis, and SARS-Related Coronavirus 2, Isolate hCoV-19/South Africa/KRISP-K005325/2020, NR-54009, contributed by Alex Sigal and Tulio de Oliveria. Remdesivir was purchased from Sigma-Aldrich. GC-376 was purchased from Selleckchem (Houston, TX, USA).

#### Expression of recombinant Mpro

A codon-optimized gene fragment of Orf1a (encoding residues 3255–3558) was synthesized by IDT DNA Technologies and cloned into a modified pETDuet vector to express a 6xHis-SUMO-Mpro fusion protein in bacteria. After expression using autoinduction, cells were resuspended and lysed in a buffer containing 50 mM Tris pH 8.5, 300 mM NaCl, 20 mM imidazole, and 5 mM 2-mercaptoethanol. In addition, 1 mM phenylmethysulfonyl fluoride, 0.5 mg/mL lysozyme, and 1% Tween-20 were added before sonication and cell lysate was clarified by centrifugation at 32,500 × *g* for 45 min. The lysate was applied to a Ni-NTA superflow column (QIAGEN), washed, and eluted using a buffer containing 25 mM Tris pH 8.5, 300 mM NaCl, 300 mM imidazole, and 5 mM 2-mercaptoethanol. The fusion protein was concentrated and applied to a Superdex 75 prep grade (Cytiva) size exclusion column equilibrated with lysis buffer. Fractions containing the fusion protein were pooled and digested overnight with ULP1 Sumo protease (purified in house). The following day, 6xHis-SUMO, ULP1, and undigested impurities were removed by reverse Ni-NTA chromatography. Mpro protein was then concentrated and applied to Superdex 75 prep grade equilibrated with 20 mM HEPES pH 7.5, 50 mM NaCl, and 2 mM DTT. The purity of the Mpro was confirmed using SDS-PAGE (>95%). Fractions were aliquoted, flash frozen, and stored at −80°C.

#### Mpro enzymatic assay

Recombinant Mpro was obtained and Mpro enzymatic assays were performed as previously described (34). Briefly, 5 µL of 25 nM recombinant Mpro protein was diluted in 25 mM HEPES (pH 7.4), 150 mM NaCl, 5 mM DTT, and 0.005% Tween was dispensed into black 384-well plates. Test compounds were serially diluted into 100% DMSO, and 100 nL was added to Mpro dilutions using a Janus MDT Nanohead (PerkinElmer). Wells were then treated with 5 µL of 5 µM fluorogenic substrate ([DABCYL]-Lys -Thr-Ser-Ala-Val-Leu-Gln-Ser-Gly-Phe-Arg-Lys-Met-Glu-(EDANS)-NH2; Bachem, Vista, CA, USA) and monitored for fluorescence at 355 nm excitation and 460 nm emission every 5 min for up to 120 min using an Envision plate reader (PerkinElmer). The rate of substrate cleavage was determined using linear regression of the raw data values obtained during the time course. Slopes of these progress curves were then normalized to percent inhibition, where 100% is equal to the rate in the absence of Mpro and 0% is equal to the cleavage rate in the presence of Mpro and 0.1% DMSO.

#### Cathepsin L enzymatic assay

The assays contained 25 pM cathepsin L (RD Systems: 952-CY-010), 5 µM LR-AMC, 100 nL of test compound in 100% DMSO, in a total of 10 µL of 20 mM potassium phosphate, pH 6.0, 150 mM NaCl, 0.005% Tween20, and 5 mM DTT in black low volume 384-well plates. The production of AMC was followed at 5-min intervals with excitation at 355 nm and emission at 460 nm using an Envision microplate reader (PerkinElmer). Reaction rates were determined by linear regression of the resulting progress curves. Rates were normalized to % inhibition, where 0% is equal to the rate in the presence enzyme, and 100% is equal to the rate in the absence of enzyme. Nonlinear regression fits of the data to a one-site dose-response curve were performed using XLFit (IDBS).

#### Cathepsin B enzymatic assay

Assays contained 0.6 nM cathepsin B (RD Systems: 953-CY-010), 25 µM Z-LR-AMC, and 100 nL of test compound in 100% DMSO in a total of 10 µL of 50 mM MES, pH 5.0, 150 mM NaCl, 0.05% CHAPS, and 5 mM DTT in black low volume 384-well plates. The production of AMC was followed at 5-min intervals at 355 nm excitation and 460 nm emission in an Envision microplate reader (PerkinElmer). Reaction rates were determined by linear regression of the resulting progress curves. Rates were normalized to percent inhibition, where 0% is equal to the rate in the presence enzyme, and 100% is equal to the rate in the absence of enzyme. Nonlinear regression fits of the data to a one-site dose-response curve were performed using XLFit (IDBS).

#### Thrombin enzymatic assay

Assays contained 25 pM thrombin (RD Systems: 1473-SE-010), 25 µM BOC-PVR-AMC, and 100 nL of test compound in 100% DMSO, in a total of 10 µL of 50 mM Tris, pH 7.0, 100 mM NaCl, 10 mM CaCl_2_, and 0.005% Tween20 in black low volume 384-well plates. The production of AMC was followed at 5-min intervals at 355 nm excitation and 460 nm emission in an Envision microplate reader (PerkinElmer). Reaction rates were determined by linear regression of the resulting progress curves. Rates were normalized to % inhibition, where 0% is equal to the rate in the presence of enzyme, and 100% is equal to the rate in the absence of enzyme. Nonlinear regression fits of the data to a one-site dose-response curve were performed using XLFit (IDBS).

#### Resazurin-based cell viability assay

2 * 10^4^ Vero-E6 cells were plated per well in 96-well plates and incubated before addition of compounds in duplicate, followed by further incubation for an additional 96 h. Resazurin (Sigma Aldrich) was then added to a final concentration of 20 µg/mL, and cells were incubated for an additional 4 h. Resazurin-induced fluorescence was then measured using a ClarioStar plate reader (BMG Labtech). Background fluorescence was subtracted from wells containing resazurin and media but no cells and normalized to cells treated with 0.1% DMSO.

#### Virus generation

3 * 10^6^ Vero-E6 cells were incubated in 15 mL of media for 24 h, replaced with 10 mL fresh media, and incubated with virus at a multiplicity of infection of 0.001. Cells were incubated for 5–7 days until clear CPE was observed throughout the flask. Media was harvested and stored at −80°C. To determine virus titers, Vero-E6 cells were plated in 96-well format at 20,000 cells per well, incubated for 24 h, and then washed and incubated in fresh media containing fivefold serial dilutions of thawed virus aliquot, followed by an additional 4-day incubation. Wells were then scored visually for the presence of CPE. TCID_50_ were then calculated using the Reed-Muench method.

#### Live virus cell viability assay

Vero-E6 cells were plated in D10+ at 2 * 10^4^ cells per well in 96-well format, and compounds were added to cells in triplicate at stated concentrations. After 2 h of incubation, cells were infected with 50× TCID_50_ of virus (USA-WA1/2020) and incubated for 96 h. Cells were then treated with resazurin to a final concentration of 20 µg/mL and incubated for an additional 4 h. Cells were fixed with paraformaldehyde to a final concentration of 4% and incubated at room temperature for at least 30 min to inactivate virus. Resazurin-induced fluorescence was then monitored as described above.

#### Live virus high-content imaging assay

Vero-E6 cells were plated in D10+ to 5,000 cells per well in 384-well format, and compounds were added to cells in triplicate at stated concentrations. After 2 h of incubation, cells were infected with 150× TCID_50_ of the virus and incubated for 48 h before being fixed with paraformaldehyde to a final concentration of 4% to inactivate the virus. Immunostaining was then performed using primary anti-SARS-CoV-2 nucleocapsid primary antibody (HL344; GeneTex, Irvine, CA) at 1:1,000 dilution and goat anti-rabbit IgG Alexa Fluor 555 secondary antibody at 1:2,000 dilution (Thermo Fisher, Waltham, MA). Cells were also counterstained with 1 µg/mL Hoechst. High-content imaging was performed across nine nonoverlapping images per well using a Nikon Eclipse Ti inverted microscope and Nikon NIS Elements AR software v.5.30.02 (Nikon Americas, Inc. Melville, NY). For each image, cell nuclei and nucleocapsid-positive cells were counted, with nucleocapsid-positive cells reported as the percentage of total nuclei in each image.

#### Data analysis

EC_50_ were calculated using nonlinear regression of a one-side binding model using GraphPad Prism v.9.1.2 (GraphPad, San Diego, CA, USA). All data are presented as the mean ± SEM from at least three independent experiments. Synergism from drug combinations was determined using the Bliss independence model as described previously (39). Statistical significance for synergy was determined using Student’s paired *t*-test, where a two-sided *P*-value of 0.05 was considered significant.

#### Mouse liver microsome stability assay

Test compounds (0.5 µM) were incubated with liver microsomes (0.5 µg/mL) and an NADPH-regenerating system (cofactor solution), and samples were taken at various time points, quenched with an acetonitrile solution containing an internal standard, and then analyzed by liquid chromatography with tandem mass spectrometry (LC-MS/MS). These data provide the half-life of parent remaining and intrinsic clearance (CLint) determined from the first-order elimination constant by nonlinear regression. Thirty microliters of 1.5 µM spiking solution containing 0.75 mg/mL microsomes solution was dispensed into the assay plates designated for different time points (0, 5, 15, 30, 45 min) on ice. For the 0-min time point, 135 µL of acetonitrile (ACN) containing internal standard (IS) was added to the wells of a 0-min plate, followed by the addition of 15 µL of NADPH stock solution (6 mM). All other plates were pre-incubated at 37°C for 5 min. Fifteen microliters of NADPH stock solution (6 mM) was then added to the plates to start the reaction and timing. At 5 min, 15 min, 30 min, and 45 min, 135 µL of ACN containing IS was added to the wells of corresponding plates, respectively, to stop the reaction. After quenching, plates were shaken with a vibrator (MTS 2/4 digital microtiter shaker, IKA, Wilmington, NC, USA) for 10 min (600 rpm/min) and then centrifuged at 5,594 *g* for 15 min. Fifty microliters of supernatant was then transferred from each well into a 96-well sample plate containing 50 µL of ultra-pure water for LC/MS analysis.

#### Hamster pharmacokinetics

To monitor pharmacokinetics of compound **1** following single intraperitoneal administration to male hamsters, compound 1 was prepared prior to use in 10% DMSO, 10% Solutol HS 15, plus 80% PBS in a concentration of 2 mg/mL by weighing 19.65 mg of compound 1 into a new vial and then adding 0.982 mL DMSO into the vial containing the compound and vortexing the vial for 2 min. In addition, 0.982 mL Solutol HS 15 was then added into the vial containing the compound, and the vial was vortexed for 2 min. Also, 7.860 mL of PBS was then added into the vial which was further vortexed for 3 min and sonicated for 0.5 min. Male hamsters weighing 89 g–104 g (*N* = 18) were purchased from Beijing Weitong Lihua Laboratory Animal Co. Ltd. Hamsters were fasted overnight and fed at 4 h post-dosing. The compound was administered 10 mg/kg (5 mL/kg) via intraperitoneal injection. Sampling was done at 0.25, 0.5, 1, 2, 4, and 6 h post dose, six time points, by terminal bleeding for plasma and lung at each time point. Approximately 150 µL blood/time point was collected into the K2EDTA tube via the jugular vein. The blood sample was put on wet ice and centrifuged to obtain a plasma sample (2,000 *g*, 5 min under 4°C) within 15 min. Lung collection was performed by making a mid-line incision in the animals’ chest and abdomen, the skin was retracted, and the lung was exposed after cutting off the ribs and removing other organs using surgical scissors and forceps. The lung was removed using surgical scissors and then rinsed with cold saline. The lung was placed in screw-top tubes and then stored under −70°C until analysis. Lung tissue was homogenized for 2 min with two volumes (vol/wt) of PBS (pH 7.4) immediately before analysis by LC-MS/MS.

### *In vivo* biological studies

#### SARS-CoV-2 viral stocks

SARS-related coronavirus 2, Isolate hCoV-19/USA/GA-EHC-2811C/2021 (Lineage B.1.1.529; Omicron variant; # NR-56481) was obtained through BEI Resources. The virus was propagated in Calu-3 cells and used to titer the viral stock on Vero cells, as described by us previously (43). The viral stocks used in animal studies were generated in passages 1–2 of the initial stock obtained from BEI Resources.

#### Drug and vehicle

Initially, 1.5 g of compound 1 was dissolved in 1.875 mL of DMSO. Using this solution, we prepared a working formulation of 28 mg/mL of compound 1 having a final concentration of 3.5% DMSO, 20% of Solutol, and 76.5% PBS respectively. We inject 0.5 mL of this drug formulation (14 mg of compound 1) twice daily to each hamster (140 g body weight).

#### Hamster study

Twenty-five male SGHs (*Mesocricetus auratus*) were obtained from Charles River Laboratories at 8–10 weeks of age. For 4 days, the animals were acclimated or quarantined at the UNMC Animal Facility, Comparative Medicine. After 4 days, the hamsters were moved to an animal biosafety level 3 (ABSL-3) facility. Hamsters were divided into three groups [group I (*n* = 10), group II (*n* = 10), and group III (*n* = 5)]. Group I was treated with the vehicle having 3.5% DMSO, 20% of Solutol, and 76.5% PBS (b.i.d.); group II was treated with compound 1 (100 mg/kg b.i.d.), and group III was treated with PF-332 (50 mg/kg b.i.d.) intraperitoneally. Two hours after the first treatment, animals were infected intranasally with SARS-CoV-2 Omicron at 0.5 * 10^3^ PFU (100 µL). Body weight and temperature were measured every day up to day 4 post-infection. All animals were necropsied on day 4 post-infection, and lung tissues were collected in 10% formalin, frozen on snap-frozen ice, and archived at −80°C for downstream experiments.

#### RNA isolation and qRT-PCR

Frozen lung tissues were homogenized in RLT tissue lysis buffer using TissueLyser LT (QIAGEN, USA), and RNA was extracted using the RNeasy Mini Kit (QIAGEN) according to manufacturer specifications. One-step qRT-PCR was performed to quantify viral (E gene and N gene) RNA from lung tissues using specific primer and probes and QuantStudio3 real-time PCR system (Applied Biosystems) per manufacturer’s specifications. SARS-CoV-2 E gene-specific primers and probe are as follows: E_Sarbeco_F1: 5′–ACAGGTACGTTAATAGTTAATAGCGT–3′, E_Sarbeco_R2: 5′–ATATTGCAGCAGTACGCACACA–3’, and E_Sarbeco_P1: 5′– FAM–ACACTAGCCATCCTTACTGCGCTTCG-BHQ1–3′. Viral RNA copies in oral swabs and lungs were quantitated using dilutions of SARS-CoV-2 standards with a known concentration of RNA copies.

#### H&E staining

Lung tissues were fixed in 10% neutral buffered formalin for at least 3 days. Tissues were placed in cassettes and processed in an STP 120 (Thermo Scientific) tissue processor using a graded series of ethanol, xylene, and paraffin wax. Formalin-fixed paraffin-embedded (FFPE) tissue blocks were cut into 5 µM sections and mounted on slides. The tissue sections were deparaffinized and stained with hematoxylin and eosin Y. Dehydrated tissues were mounted with coverslips, and images were captured under a microscope. The slides were blinded and were assessed by a qualified pathologist for gross pathological changes and clinical features.

#### Infectious virus titer estimation

Infectious virus titer estimation was performed as described previously (2). Frozen lung tissues were homogenized in 500 µL DMEM with bead disruption using TissueLyser LT (QIAGEN). The homogenized samples were clarified at low-speed centrifugation (6,000 *g* for 10 min), and the supernatant was collected and stored at −80°C. Vero-E6 cells were seeded in 96-well plates at 2.5 × 10^4^ cells per well and cultured in DMEM with 10% FBS overnight. The cells were treated with a dilution series of clarified lung tissue homogenates and incubated for an hour at 37°C with 5% CO_2_. Then, the virus inoculum was removed, and the cells were overlaid with 100 µL of prewarmed 0.85% methylcellulose (Sigma-Aldrich, #M0512-250G). The plates were incubated at 37°C for 72 h, and the overlaid methylcellulose was removed and washed five times with PBS. The cells were fixed with 4% paraformaldehyde in PBS for 30 min, and washed three times with PBS, and 100 µL of permeabilization buffer containing 0.1% bovine serum albumin (BSA) (VWR, #0332) and 0.1% of TritonX-100 (Sigma-Aldrich) in PBS was added to permeabilize cells for 20 min at room temperature. After permeabilization, cells were incubated with 1:1,000 dilution of rabbit anti–SARS-CoV-2 spike (S1) primary antibody (Sino Biological) overnight at 4°C on a shaker. The next day, cells were washed three times with PBS, and 50 µL of 1:2,000 horseradish peroxidase (HRP)-conjugated goat anti-rabbit secondary antibody (Jackson ImmunoResearch) was added and incubated for 2 h at room temperature. After incubation, the cells were washed three times with PBS, and 100 µL of 3,3′,5,5′-tetramethylbenzidine substrate solution was added and incubated for 15–30 min. The plates were washed with PBS, and foci were visualized on a CTL Analyzer-ImmunoSpot for virus foci and counted in drug-treated vs untreated lung samples, and virus titers were calculated using the following formula: PFU/gram of lung tissue = (average number of foci / volumes of virus added) × the dilution factor.

#### Statistics

Statistical analysis was performed in GraphPad prism 8. The difference in the infectious virus titer between the study groups was assessed by the Mann-Whitney *t*-test. *P*-values of less than 0.05 were considered statistically significant.

## SUPPLEMENTAL MATERIAL

Supplemental material aac.00562-24-s0001.pdf Synthesis details for compounds.

ASM does not own the copyrights to Supplemental Material that may be linked to, or accessed through, an article. The authors have granted ASM a non-exclusive, world-wide license to publish the Supplemental Material files. Please contact the corresponding author directly for reuse.

## References

[1] Olliaro P, Torreele E, Vaillant M. 2021. COVID-19 vaccine efficacy and effectiveness-the elephant (not) in the room. Lancet Microbe 2:e279–e280. doi:10.1016/S2666-5247(21)00069-0 33899038 PMC8057721

[2] Lopez Bernal J, Andrews N, Gower C, Gallagher E, Simmons R, Thelwall S, Stowe J, Tessier E, Groves N, Dabrera G, Myers R, Campbell CNJ, Amirthalingam G, Edmunds M, Zambon M, Brown KE, Hopkins S, Chand M, Ramsay M. 2021. Effectiveness of covid-19 vaccines against the B.1.617.2 (delta) variant. N Engl J Med 385:585–594. doi:10.1056/NEJMoa2108891 34289274 PMC8314739

[3] Planas D, Veyer D, Baidaliuk A, Staropoli I, Guivel-Benhassine F, Rajah MM, Planchais C, Porrot F, Robillard N, Puech J, et al.. 2021. Reduced sensitivity of SARS-CoV-2 variant delta to antibody neutralization. Nature 596:276–280. doi:10.1038/s41586-021-03777-9 34237773

[4] Wang P, Nair MS, Liu L, Iketani S, Luo Y, Guo Y, Wang M, Yu J, Zhang B, Kwong PD, Graham BS, Mascola JR, Chang JY, Yin MT, Sobieszczyk M, Kyratsous CA, Shapiro L, Sheng Z, Huang Y, Ho DD. 2021. Antibody resistance of SARS-CoV-2 variants B.1.351 and B.1.1.7. Nature 593:130–135. doi:10.1038/s41586-021-03398-2 33684923

[5] Garcia-Beltran WF, Lam EC, St Denis K, Nitido AD, Garcia ZH, Hauser BM, Feldman J, Pavlovic MN, Gregory DJ, Poznansky MC, Sigal A, Schmidt AG, Iafrate AJ, Naranbhai V, Balazs AB. 2021. Multiple SARS-CoV-2 variants escape neutralization by vaccine-induced humoral immunity. Cell 184:2372–2383. doi:10.1016/j.cell.2021.03.013 33743213 PMC7953441

[6] Daniloski Z, Jordan TX, Ilmain JK, Guo X, Bhabha G, tenOever BR, Sanjana NE. 2021. The spike D614G mutation increases SARS-CoV-2 infection of multiple human cell types. Elife 10:e65365. doi:10.7554/eLife.65365 33570490 PMC7891930

[7] Zhou D, Dejnirattisai W, Supasa P, Liu C, Mentzer AJ, Ginn HM, Zhao Y, Duyvesteyn HME, Tuekprakhon A, Nutalai R, et al.. 2021. Evidence of escape of SARS-CoV-2 variant B.1.351 from natural and vaccine-induced sera. Cell 184:2348–2361. doi:10.1016/j.cell.2021.02.037 33730597 PMC7901269

[8] Li Q, Nie J, Wu J, Zhang L, Ding R, Wang H, Zhang Y, Li T, Liu S, Zhang M, et al.. 2021. SARS-CoV-2 501Y.V2 variants lack higher infectivity but do have immune escape. Cell 184:2362–2371. doi:10.1016/j.cell.2021.02.042 33735608 PMC7901273

[9] Kokic G, Hillen HS, Tegunov D, Dienemann C, Seitz F, Schmitzova J, Farnung L, Siewert A, Höbartner C, Cramer P. 2021. Mechanism of SARS-CoV-2 polymerase stalling by remdesivir. Nat Commun 12:279. doi:10.1038/s41467-020-20542-0 33436624 PMC7804290

[10] Cox RM, Wolf JD, Plemper RK. 2021. Therapeutically administered ribonucleoside analogue MK-4482/EIDD-2801 blocks SARS-CoV-2 transmission in ferrets. Nat Microbiol 6:11–18. doi:10.1038/s41564-020-00835-2 33273742 PMC7755744

[11] Agostini ML, Pruijssers AJ, Chappell JD, Gribble J, Lu X, Andres EL, Bluemling GR, Lockwood MA, Sheahan TP, Sims AC, Natchus MG, Saindane M, Kolykhalov AA, Painter GR, Baric RS, Denison MR. 2019. Small-molecule antiviral β-d-N^4^-hydroxycytidine inhibits a proofreading-intact coronavirus with a high genetic barrier to resistance. J Virol 93:e01348-19. doi:10.1128/JVI.01348-19 31578288 PMC6880162

[12] Mei M, Tan X. 2021. Current strategies of antiviral drug discovery for COVID-19. Front Mol Biosci 8:671263. doi:10.3389/fmolb.2021.671263 34055887 PMC8155633

[13] Owen DR, Allerton CMN, Anderson AS, Aschenbrenner L, Avery M, Berritt S, Boras B, Cardin RD, Carlo A, Coffman KJ, et al.. 2021. An oral SARS-CoV-2 M^pro^ inhibitor clinical candidate for the treatment of COVID-19. Sci 374:1586–1593. doi:10.1126/science.abl4784 34726479

[14] Marzolini C, Kuritzkes DR, Marra F, Boyle A, Gibbons S, Flexner C, Pozniak A, Boffito M, Waters L, Burger D, Back DJ, Khoo S. 2022. Recommendations for the management of drug-drug interactions between the COVID-19 antiviral nirmatrelvir/ritonavir (Paxlovid) and comedications. Clin Pharmacol Ther 112:1191–1200. doi:10.1002/cpt.2646 35567754 PMC9348462

[15] Thiel V, Ivanov KA, Putics Á, Hertzig T, Schelle B, Bayer S, Weißbrich B, Snijder EJ, Rabenau H, Doerr HW, Gorbalenya AE, Ziebuhr J. 2003. Mechanisms and enzymes involved in SARS coronavirus genome expression. J Gen Virol 84:2305–2315. doi:10.1099/vir.0.19424-0 12917450

[16] Fan K, Wei P, Feng Q, Chen S, Huang C, Ma L, Lai B, Pei J, Liu Y, Chen J, Lai L. 2004. Biosynthesis, purification, and substrate specificity of severe acute respiratory syndrome coronavirus 3C-like proteinase. J Biol Chem 279:1637–1642. doi:10.1074/jbc.M310875200 14561748 PMC7980035

[17] Zhang L, Lin D, Sun X, Curth U, Drosten C, Sauerhering L, Becker S, Rox K, Hilgenfeld R. 2020. Crystal structure of SARS-CoV-2 main protease provides a basis for design of improved α-ketoamide inhibitors. Science 368:409–412. doi:10.1126/science.abb3405 32198291 PMC7164518

[18] Turk V, Stoka V, Vasiljeva O, Renko M, Sun T, Turk B, Turk D. 2012. Cysteine cathepsins: from structure, function and regulation to new frontiers. Biochim Biophys Acta 1824:68–88. doi:10.1016/j.bbapap.2011.10.002 22024571 PMC7105208

[19] Vandyck K, Deval J. 2021. Considerations for the discovery and development of 3-chymotrypsin-like cysteine protease inhibitors targeting SARS-CoV-2 infection. Curr Opin Virol 49:36–40. doi:10.1016/j.coviro.2021.04.006 34029993 PMC8075814

[20] Ma C, Xia Z, Sacco MD, Hu Y, Townsend JA, Meng X, Choza J, Tan H, Jang J, Gongora MV, Zhang X, Zhang F, Xiang Y, Marty MT, Chen Y, Wang J. 2021. Discovery of Di- and trihaloacetamides as covalent SARS-CoV-2 main protease inhibitors with high target specificity. J Am Chem Soc 143:20697–20709. doi:10.1021/jacs.1c08060 34860011 PMC8672434

[21] Dolle RE, Prasad CV, Prouty CP, Salvino JM, Awad MM, Schmidt SJ, Hoyer D, Ross TM, Graybill TL, Speier GJ, Uhl J, Miller BE, Helaszek CT, Ator MA. 1997. Pyridazinodiazepines as a high-affinity, P2-P3 peptidomimetic class of interleukin-1 beta-converting enzyme inhibitor. J Med Chem 40:1941–1946. doi:10.1021/jm9701637 9207934

[22] LaPlante SR, Gillard JR, Jakalian A, Aubry N, Coulombe R, Brochu C, Tsantrizos YS, Poirier M, Kukolj G, Beaulieu PL. 2010. Importance of ligand bioactive conformation in the discovery of potent indole-diamide inhibitors of the hepatitis C virus NS5B. J Am Chem Soc 132:15204–15212. doi:10.1021/ja101358s 20942454

[23] Dolle RE, Singh J, Rinker J, Hoyer D, Prasad CV, Graybill TL, Salvino JM, Helaszek CT, Miller RE, Ator MA. 1994. Aspartyl alpha-((1-phenyl-3-(trifluoromethyl)-pyrazol-5-yl)oxy)methyl ketones as interleukin-1 beta converting enzyme inhibitors. Significance of the P1 and P3 amido nitrogens for enzyme-peptide inhibitor binding. J Med Chem 37:3863–3866. doi:10.1021/jm00049a001 7966144

[24] Dolle RE, Prouty CP, Prasad CV, Cook E, Saha A, Ross TM, Salvino JM, Helaszek CT, Ator MA. 1996. First examples of peptidomimetic inhibitors of interleukin-1 beta converting enzyme. J Med Chem 39:2438–2440. doi:10.1021/jm9601516 8691439

[25] Powers JC, Asgian JL, Ekici OD, James KE. 2002. Irreversible inhibitors of serine, cysteine, and threonine proteases. Chem Rev 102:4639–4750. doi:10.1021/cr010182v 12475205

[26] Lee J, Worrall LJ, Vuckovic M, Rosell FI, Gentile F, Ton AT, Caveney NA, Ban F, Cherkasov A, Paetzel M, Strynadka NCJ. 2020. Crystallographic structure of wild-type SARS-CoV-2 main protease acyl-enzyme intermediate with physiological C-terminal autoprocessing site. Nat Commun 11:5877. doi:10.1038/s41467-020-19662-4 33208735 PMC7674412

[27] Ullrich S, Nitsche C. 2020. The SARS-CoV-2 main protease as drug target. Bioorg Med Chem Lett 30:127377. doi:10.1016/j.bmcl.2020.127377 32738988 PMC7331567

[28] Itoh K, Kori M, Inada Y, Nishikawa K, Kawamatsu Y, Sugihara H. 1986. Synthesis and angiotensin converting enzyme inhibitory activity of 1,5-benzothiazepine and 1,5-benzoxazepine derivatives. I. Chem Pharm Bull (Tokyo) 34:1128–1147. doi:10.1248/cpb.34.1128 3015433

[29] Itoh K, Kori M, Inada Y, Nishikawa K, Kawamatsu Y, Sugihara H. 1986. Synthesis and angiotensin converting enzyme-inhibitory activity of 1,5-benzothiazepine and 1,5-benzoxazepine derivatives. III. Chem Pharm Bull (Tokyo) 34:3747–3761. doi:10.1248/cpb.34.3747 3028653

[30] Wang YC, Yang WH, Yang CS, Hou MH, Tsai CL, Chou YZ, Hung MC, Chen Y. 2020. Structural basis of SARS-CoV-2 main protease inhibition by a broad-spectrum anti-coronaviral drug. Am J Cancer Res 10:2535–2545. 32905393 PMC7471349

[31] Krantz A, Copp LJ, Coles PJ, Smith RA, Heard SB. 1991. Peptidyl (acyloxy)methyl ketones and the quiescent affinity label concept: the departing group as a variable structural element in the design of inactivators of cysteine proteinases. Biochemistry 30:4678–4687. doi:10.1021/bi00233a007 2029515

[32] Jain RP, Vederas JC. 2004. Structural variations in keto-glutamines for improved inhibition against hepatitis A virus 3C proteinase. Bioorg Med Chem Lett 14:3655–3658. doi:10.1016/j.bmcl.2004.05.021 15203137

[33] Hoffman RL, Kania RS, Brothers MA, Davies JF, Ferre RA, Gajiwala KS, He M, Hogan RJ, Kozminski K, Li LY, Lockner JW, Lou J, Marra MT, Mitchell LJ, Murray BW, Nieman JA, Noell S, Planken SP, Rowe T, Ryan K, Smith GJ, Solowiej JE, Steppan CM, Taggart B. 2020. Discovery of ketone-based covalent inhibitors of coronavirus 3CL proteases for the potential therapeutic treatment of COVID-19. J Med Chem 63:12725–12747. doi:10.1021/acs.jmedchem.0c01063 33054210

[34] Ma C, Sacco MD, Hurst B, Townsend JA, Hu Y, Szeto T, Zhang X, Tarbet B, Marty MT, Chen Y, Wang J. 2020. Boceprevir, GC-376, and calpain inhibitors II, XII inhibit SARS-CoV-2 viral replication by targeting the viral main protease. Cell Res 30:678–692. doi:10.1038/s41422-020-0356-z 32541865 PMC7294525

[35] Tietjen I, Cassel J, Register ET, Zhou XY, Messick TE, Keeney F, Lu LD, Beattie KD, Rali T, Tebas P, Ertl HCJ, Salvino JM, Davis RA, Montaner LJ. 2021. The natural stilbenoid (-)-hopeaphenol inhibits cellular entry of SARS-CoV-2 USA-WA1/2020, B.1.1.7, and B.1.351 variants. Antimicrob Agents Chemother 65:e0077221. doi:10.1128/AAC.00772-21 34543092 PMC8597786

[36] Strelow JM. 2017. A perspective on the kinetics of covalent and irreversible inhibition. SLAS Discov 22:3–20. doi:10.1177/1087057116671509 27703080

[37] Pruijssers AJ, George AS, Schäfer A, Leist SR, Gralinksi LE, Dinnon KH, Yount BL, Agostini ML, Stevens LJ, Chappell JD, et al.. 2020. Remdesivir inhibits SARS-CoV-2 in human lung cells and chimeric SARS-CoV expressing the SARS-CoV-2 RNA polymerase in mice. Cell Rep 32:107940. doi:10.1016/j.celrep.2020.107940 32668216 PMC7340027

[38] Richard K, Schonhofer C, Giron LB, Rivera-Ortiz J, Read S, Kannan T, Kinloch NN, Shahid A, Feilcke R, Wappler S, Imming P, Harris M, Brumme ZL, Brockman MA, Mounzer K, Kossenkov AV, Abdel-Mohsen M, Andrae-Marobela K, Montaner LJ, Tietjen I. 2020. The African natural product knipholone anthrone and its analogue anthralin (dithranol) enhance HIV-1 latency reversal. J Biol Chem 295:14084–14099. doi:10.1074/jbc.RA120.013031 32788215 PMC7549037

[39] Unoh Y, Uehara S, Nakahara K, Nobori H, Yamatsu Y, Yamamoto S, Maruyama Y, Taoda Y, Kasamatsu K, Suto T, Kouki K, Nakahashi A, Kawashima S, Sanaki T, Toba S, Uemura K, Mizutare T, Ando S, Sasaki M, Orba Y, Sawa H, Sato A, Sato T, Kato T, Tachibana Y. 2022. Discovery of S-217622, a noncovalent oral SARS-CoV-2 3CL protease inhibitor clinical candidate for treating COVID-19. J Med Chem 65:6499–6512. doi:10.1021/acs.jmedchem.2c00117 35352927 PMC8982737

[40] Mondal S, Chen Y, Lockbaum GJ, Sen S, Chaudhuri S, Reyes AC, Lee JM, Kaur AN, Sultana N, Cameron MD, Shaffer SA, Schiffer CA, Fitzgerald KA, Thompson PR. 2022. Dual inhibitors of main protease (M^Pro^) and cathepsin L as potent antivirals against SARS-CoV2. J Am Chem Soc 144:21035–21045. doi:10.1021/jacs.2c04626 36356199 PMC9662648

[41] Xie X, Lan Q, Zhao J, Zhang S, Liu L, Zhang Y, Xu W, Shao M, Peng J, Xia S, et al.. 2024. Structure-based design of pan-coronavirus inhibitors targeting host cathepsin L and calpain-1. Signal Transduct Target Ther 9:54. doi:10.1038/s41392-024-01758-8 38443334 PMC10914734

[42] Monti B, Sparapani M, Contestabile A. 1998. Differential toxicity of protease inhibitors in cultures of cerebellar granule neurons. Exp Neurol 153:335–341. doi:10.1006/exnr.1998.6858 9784292

[43] Acharya A, Pathania AS, Pandey K, Thurman M, Vann KR, Kutateladze TG, Challagundala KB, Durden DL, Byrareddy SN. 2022. PI3K-α/mTOR/BRD4 inhibitor alone or in combination with other anti-virals blocks replication of SARS-CoV-2 and its variants of concern including Delta and Omicron. Clin Transl Med 12:e806. doi:10.1002/ctm2.806 35390226 PMC8989379

