# Supplementary material for: Design of novel and highly selective SARS-CoV-2 main protease inhibitors
Source: Antimicrob Agents Chemother. 2024 Sep 3;68(10):e00562-24. doi: 10.1128/aac.00562-24 (PMC11459967; doi:10.1128/aac.00562-24)
Supplement: Supplemental material — Synthesis details for compounds. [file aac.00562-24-s0001.pdf]

SUPPORTING INFORMATION
DESIGN OF NOVEL AND HIGHLY SELECTIVE SARS-COV-2 MAIN PROTEASE INHIBITORS

Adi N.R. Poli,[a] Ian Tietjen,[d]* Nitesh K. Nandwana,[a] Joel Cassel,[c] Troy E. Messick,[e] Emery T. Register,[d] Frederick Keeney,[d] Rajesh Rajaiah,[e] Atul K.Verma,[e] Kabita Pandey,[e] Arpan Acharya,[e] Siddappa N. Byrareddy,[e] Luis J. Montaner,[d] Joseph M. Salvino[a,b,c,*]

[a]Medicinal Chemistry, [b]Molecular and Cellular Oncogenesis (MCO) Program, [c]The Wistar Cancer Center Molecular Screening, [d]HIV-1 Program in the Vaccine and Immunotherapy Center, [e]The Wistar Institute, Philadelphia, Pennsylvania

[e]Department of Pharmacology & Experimental Neuroscience, University of Nebraska Medical Center, Omaha, NE 68182, USA.

*To whom correspondence should be addressed.

*Joseph M. Salvino, E-mail: jsalvino@wistar.org; ORCID: 0000-0002-2184-5980.

* Ian Tietjen, E-mail:itietjen@wistar.org; ORCID: 0000-0002-8991-6490

Table of contents

**Content of Supporting Information**	Page No.
Figure 4. Synthesis Details for Compound 1, 2, 3, 4, 5, 6, 8, and 9.	
Figure 5. Synthesis of Compound 7 - Compound Spectra for 10, 11, 12, 13, 14, 15, and 7.	
Figure 6. Synthesis of Pfizer compound 18 - Compound Spectra for 16, 17, and 18.	
Figure S1: Synthesis of 21 Experimental procedures and Spectra Compounds 22, 23, 24, 25, 26, and 21.	
Table 1 compound spectra for 19 and 20.	

General Methods for Chemistry. All solvents and chemicals were used as purchased without further purification. The progress of all reactions was monitored on Merck precoated silica gel plates (with fluorescence indicator UV254) using the solvent system indicated. Column chromatography was performed with silica gel 60 (230−400 mesh ASTM) or using an automated Biotage Isolera one automated flash purification system with the solvent mixtures specified in the corresponding experiment. TLC plates were visualized by irradiation with ultraviolet light (254 nm). Proton (^1H) and carbon (^{13}C) NMR spectra were recorded on a Bruker AVANCE III 400

High-Performance Digital NMR Spectrometer using DMSO-d6 as solvent. Chemical shifts are reported in parts per million (ppm, δ) using the residual solvent line as a reference. Splitting patterns are designated using the following abbreviations: s, singlet; d, doublet; t, triplet; dd, doublet of doublet; m, multiplet; br, broad. Coupling constants (J) are reported in hertz (Hz). Compound purity was determined by LCMS and NMR. LCMS was obtained on a Waters Acquity QDa UPLC/MS mass spectrometer with an electrospray ionization (ESI) source and a PDA detector (210−400 nm). The purity of all final compounds was 95% or higher. High-resolution accurate mass LC-MS/MS data were acquired on a Thermo Q Exactive Plus mass spectrometer coupled with a Waters Nano-ACQUITY UPLC system.

Figure 4. Synthesis of Compound 1 - Compound Spectra

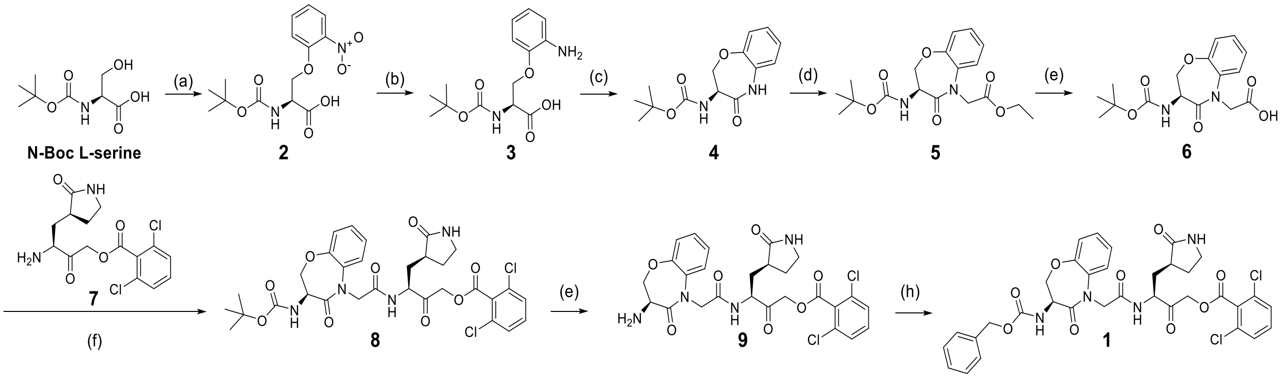

[a]**Reagents and conditions:** (a) NaH, DMF, 1-fluoro-2-nitrobenzene, 0 ºC - 40 ºC, 2 h. (b) 10 % Pd/C, H_2(g) , EtOH, rt, 16 h. (c) 50 % T_3P in CH_2Cl_2, DIPEA, -20 ºC - 0 ºC, 1 h. (d) LiHMDS, THF, ethyl 2-bromoacetate, -78 ºC - rt, 12 h. (e) NaOH, THF: MeOH: H_2O, 0 ºC - rt, 12 h. (f) 50% T_3P in CH_2Cl_2, DIPEA, -0 ºC to rt, 1 h. (S)-3-amino-2-oxo-4-((S)-2-oxopyrrolidin-3-yl)butyl 2,6-dichloro benzoate. (g) 20% TFA in CH_2Cl_2, 0 ºC - rt, 2 h (h) Cbz chloride, Et_3N, CH_2Cl_2, 0 ºC - rt, 24 h.

(*S*)-2-(Tert-butoxycarbonylamino)-3-(2-nitrophenoxy)propanoic acid (2, AP-8-248); To a 0°C suspension of NaH (60% w/w in mineral oil, 4.74 g, 118.71 mmol) in DMF (50 mL) was added a solution of (*S*)-2-(tert-butoxycarbonylamino)-3-hydroxypropanoic acid (11.6 g, 56.52 mmol) in DMF (50 mL). After 2 hours, a solution of 1-fluoro-2-nitrobenzene (8.77g, 62.12 mmol) in DMF (25 mL) was added and the resulting mixture was stirred at 0°C for 4 hours. The mixture was poured into 0°C H_2O (200 mL), acidified to pH 5.0 with 1 N aq HCl, and extracted with EtOAc. The combined organic layers were dried over Na_2SO_4, filtered, and concentrated *in vacuo*. The crude material was purified by flash chromatography to afford the title compound (15.76 g, 48.13 mmol, 85%) as thick liquid. ^{1}H NMR (400 MHz, $CDCl_3$) δ 9.88 (s, 1H), 7.83 (dd, J = 8.1, 1.3 Hz, 1H), 7.56 – 7.51 (m, 1H), 7.10 – 7.03 (m, 2H), 5.71 (d, J = 8.4 Hz, 1H), 4.78 – 4.76 (m, 1H), 4.65 – 4.62 (m, 1H), 4.39 – 4.36 (m, 1H), 1.45 (s, 9H). ^{13}C NMR (100 MHz, $CDCl_3$) δ 173.7, 155.8, 151.7, 139.6, 134.5, 125.8, 121.3, 115.1, 80.8, 69.9, 53.2, 28.3.
ESI-HRMS (m/z): calculated for $C_{14}H_{19}N_2O_7$ $(M+H)^+$ = 327.1192 found: 327.1184

(*S*)-2-(Tert-butoxycarbonylamino)-3-(2-nitrophenoxy)propanoic acid (2, AP-8-248);

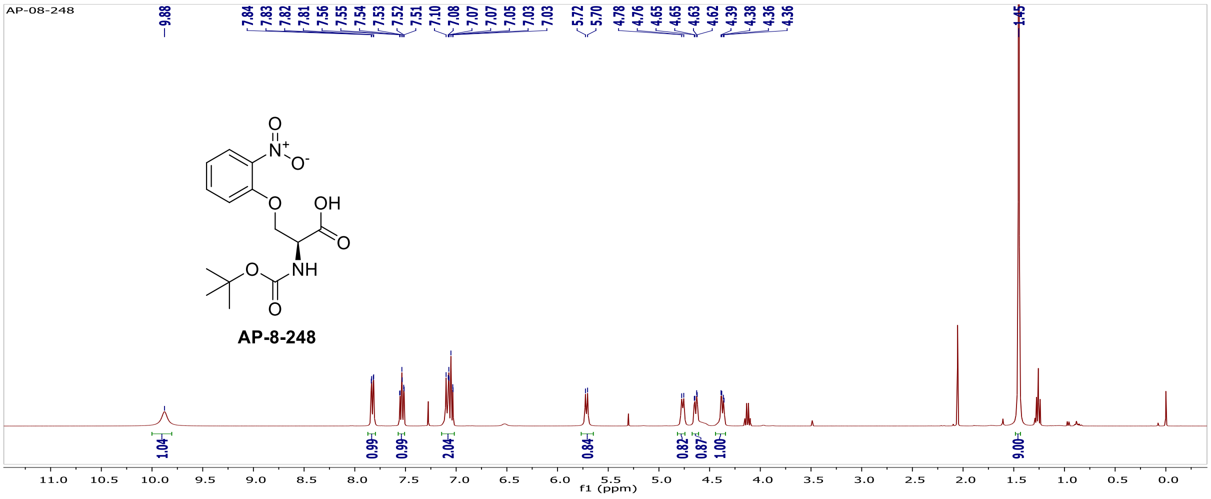

^{1}H NMR of Compound 2, AP-8-248

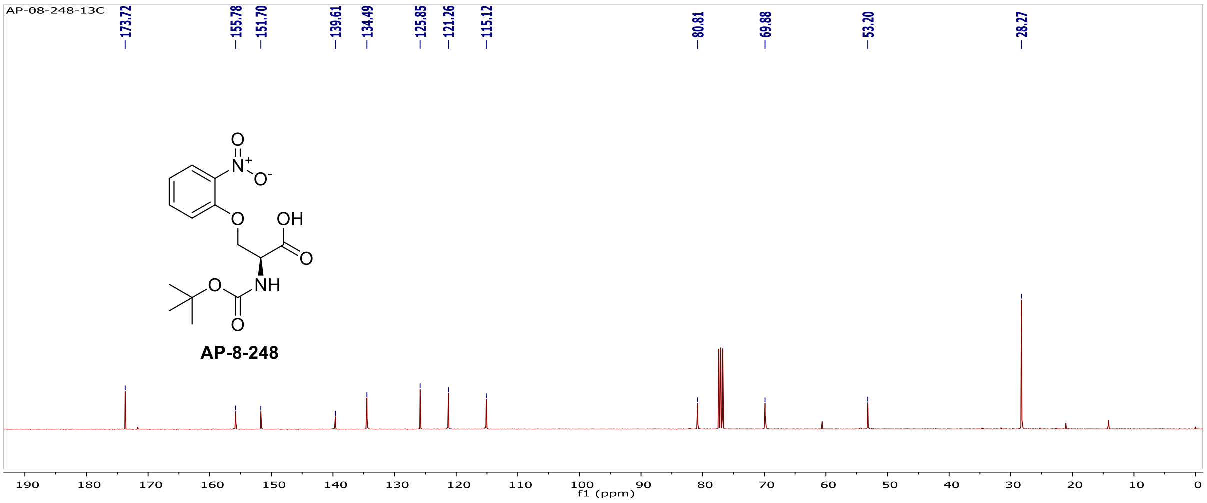

^{13}C NMR of Compound 2, AP-8-248

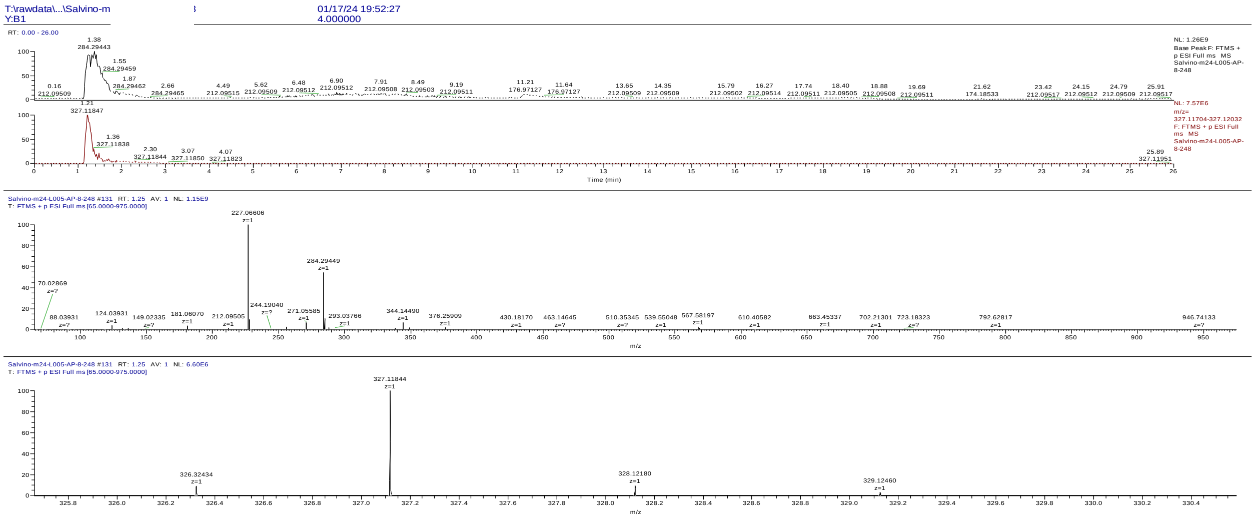

HRMS of Compound 2, AP-8-24

(*S*)-3-(2-Aminophenoxy)-2-(tert-butoxycarbonylamino) propanoic acid (3, AP-8-250); To a stirred solution of (*S*)-2-(tert-butoxycarbonylamino)-3-(2-nitrophenoxy)propanoic acid (14 g; 42.9 mmol) in 200 mL of EtOH,10% Pd/C was added (1.36 g 1.29 mmol) under nitrogen atmosphere. Nitrogen gas was switched to hydrogen gas (Balloon pressure) and stirred for 16 hours at room temperature. Completion of the reaction was confirmed by LC-MS. Catalyst was removed by passing through a small pad of celite. Filtrate was concentrated under reduced pressure to afford the title compound (12.08 g; 40.75 mmol, 95%) as a thick liquid. ^{1}H NMR (400 MHz, DMSO) δ 7.49 (d, J = 8.8 Hz, 1H), 6.74 (d, J = 7.8 Hz, 1H), 6.68 (t, J = 7.3 Hz, 1H), 6.61 – 6.59 (m, 1H), 6.47 (t, J = 7.2 Hz, 1H), 4.45 – 4.43 (m, 1H), 4.28 – 4.24 (m, 1H), 4.01 – 3.99 (m, 1H), 1.41 (s, 9H). ^{13}C NMR (100 MHz, DMSO) δ 172.1, 156.1, 145.6, 138.5, 121.9, 116.4, 114.3, 112.2, 78.7, 68.7, 53.8, 28.7.

ESI-HRMS (m/z): calculated for $C_{14}H_{21}N_2O_5$ $(M+H)^+$ = 297.1450 found: 297.1443

(S)-3-(2-Aminophenoxy)-2-(tert-butoxycarbonylamino) propanoic acid (3, AP-8-250);

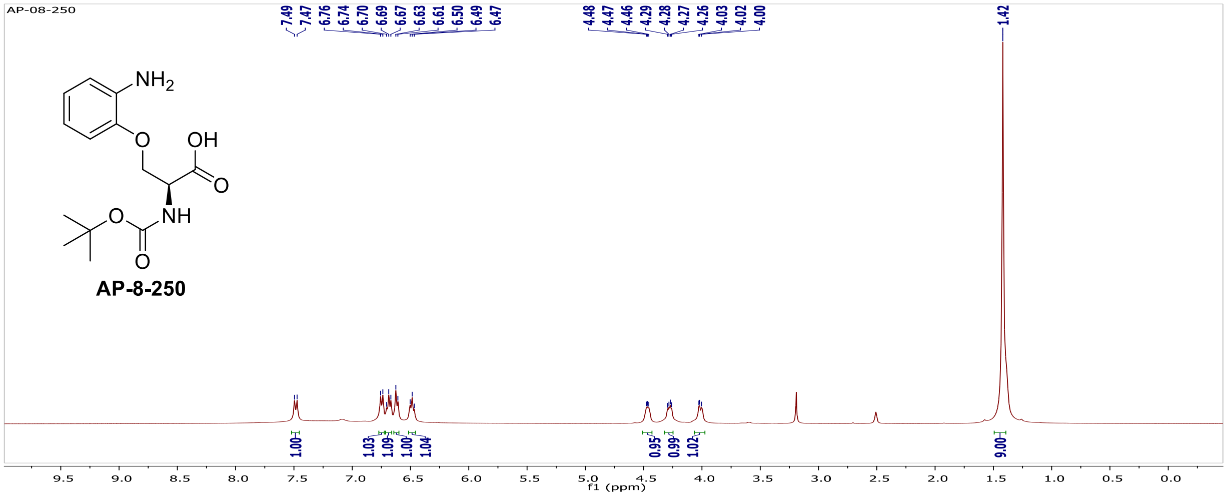

^{1}H NMR of Compound 3, AP-8-250

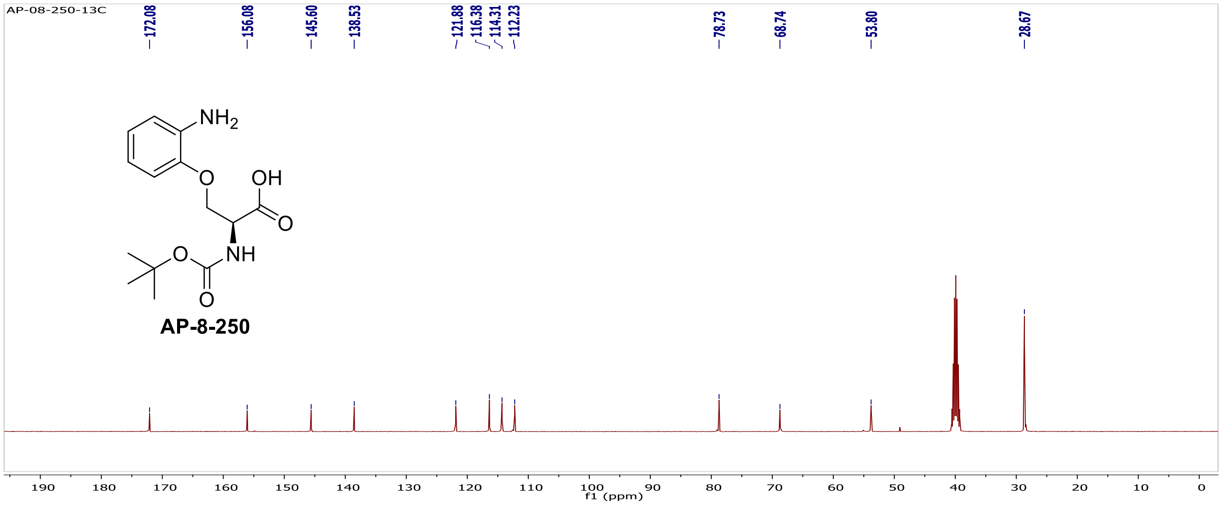

^{13}C NMR of Compound 3, AP-8-250

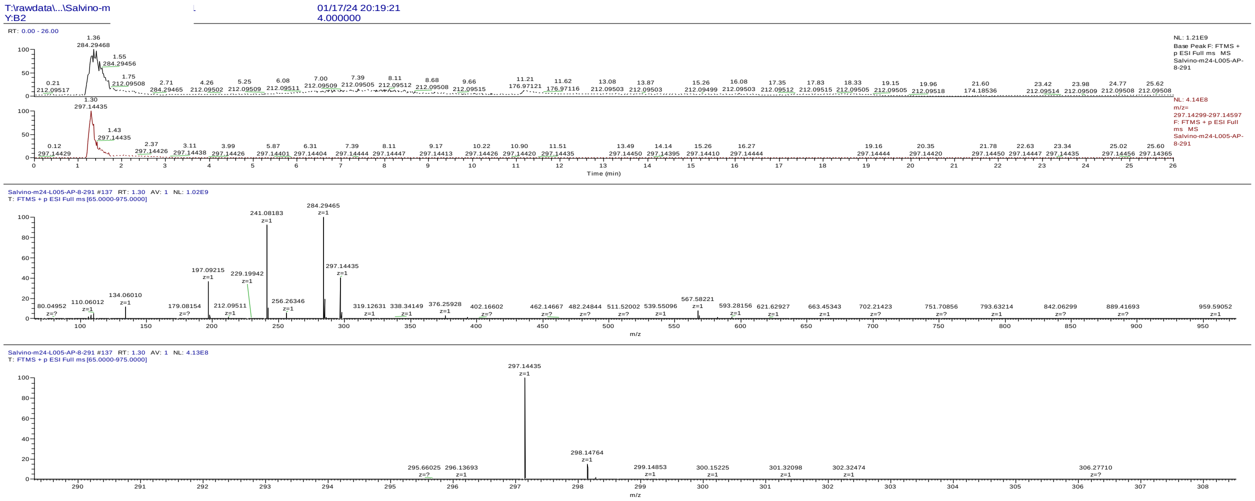

HRMS of Compound 3, AP-8-250

Tert-butyl (*S*)-(4-oxo-2,3,4,5-tetrahydrobenzo[*b*][1,4]oxazepin-3-yl)carbamate (4, AP-8-291); To a stirred solution of (*S*)-3-(2-aminophenoxy)-2-(tert-butoxycarbonylamino)propanoic acid (10.0 g; 33.75 mmol) in 200 mL of dry CH_2Cl_2 at -20 °C was added diisopropyl ethyl amine (24.12 mL; 135.0 mmol) and T3P 50% solution in CH_2Cl_2 by weight (23.6 g; 37.12 mmol) dropwise simultaneously. The reaction mixture was allowed to stir for 1hour at 0 °C. Completion of the reaction was confirmed by LC−MS. The reaction mixture was quenched with cold water and the product was extracted with CH_2Cl_2 and dried over anhydrous Na_2SO_4. The solvent was evaporated under reduced pressure and the crude product was purified by flash column chromatography to afford the title compound (8.45 g; 30.37 mmol, 90%). ^{1}H NMR (400 MHz, DMSO) δ 9.94 (s, 1H), 7.16 – 7.07 (m, 5H), 4.36 – 4.27 (m, 3H), 1.36 (s, 9H). ^{13}C NMR (100 MHz, DMSO) δ 170.8, 155.6, 149.2, 132.0, 126.0, 125.2, 122.8, 122.6, 79.0, 75.3, 51.5, 28.5.
ESI-HRMS (m/z): calculated for $C_{14}H_{19}N_2O_4$ $(M+H)^+$ = 279.1345 found: 279.1334

Tert-butyl (S)-(4-oxo-2,3,4,5-tetrahydrobenzo[b][1,4]oxazepin-3-yl)carbamate (4, AP-8-291);

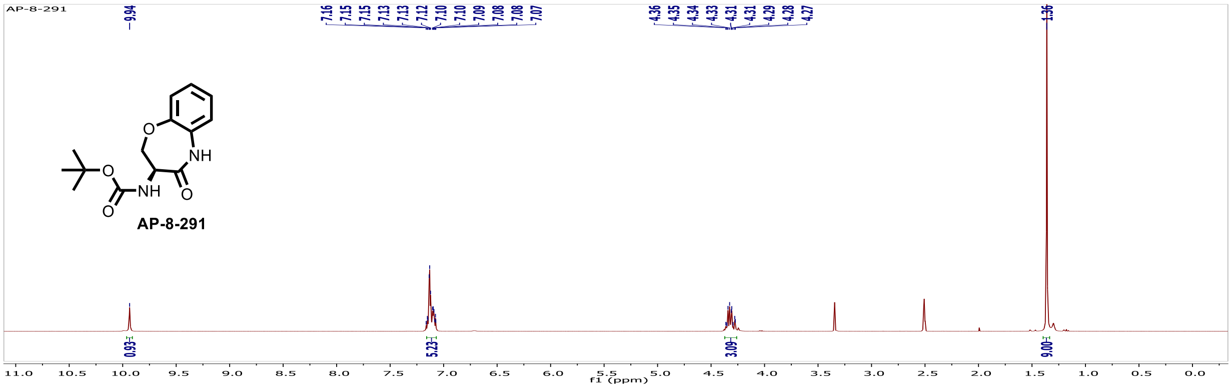

^{1}H NMR of Compound 4, AP-8-291

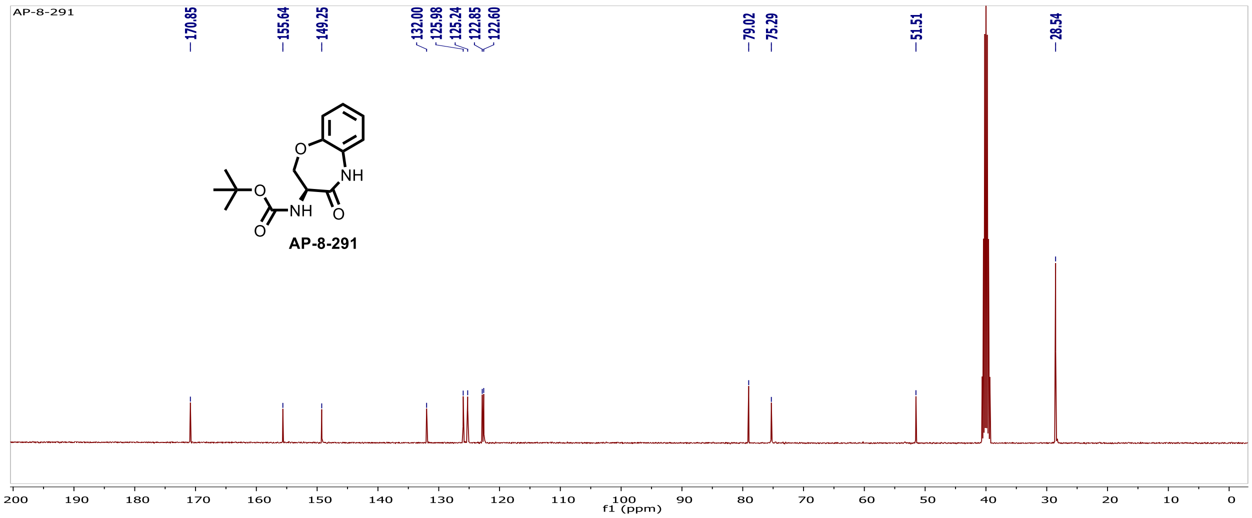

^{13}C NMR of Compound 4, AP-8-291

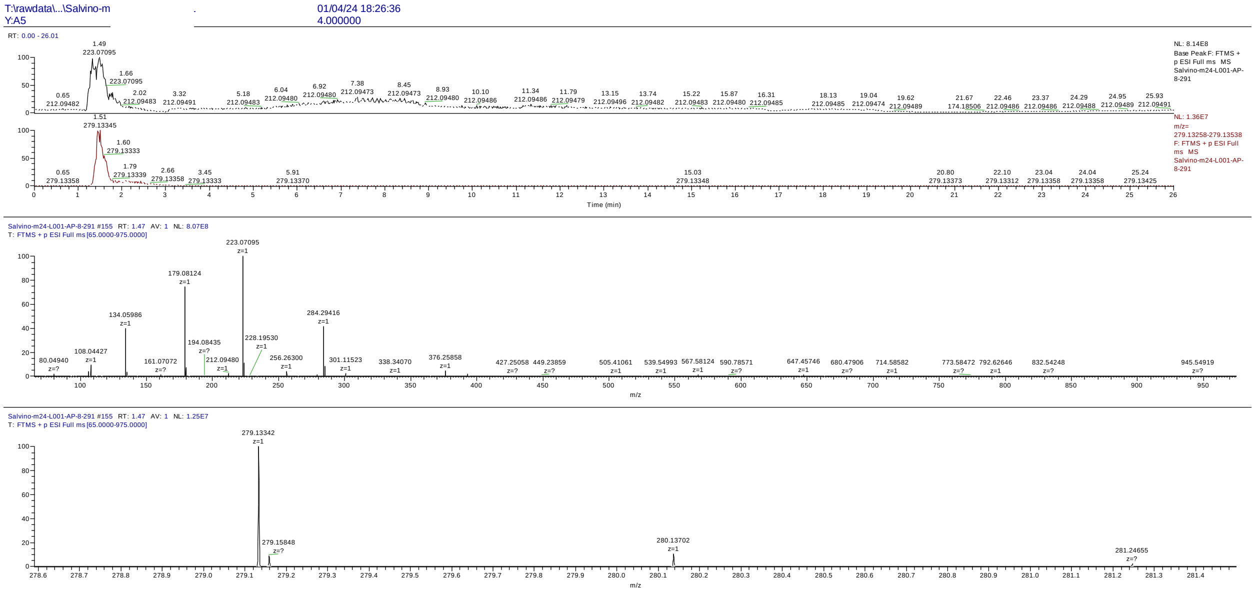

HRMS of Compound 4, AP-8-291

Ethyl (*S*)-2-(3-((tert-butoxycarbonyl)amino)-4-oxo-3,4-dihydrobenzo[*b*][1,4]oxazepin-5(2*H*)-yl)acetate (5, AP-8-293); To a stirred solution of (*S*)-tert-butyl 4-oxo-2,3,4,5-tetrahydrobenzo[*b*][1,4]oxazepin-3-ylcarbamate (4.0 g; 14.37 mmol) in 100 mL of dry THF at -70 °C, 1M in hexane LiHMDS (15.84 mL; 15.81 mmol) was added dropwise. The reaction mixture was stirred for 30 minutes at the same temperature, and ethyl 2-bromoacetate (2.64 g, 15.81 mmol) in 10 mL dry THF was added dropwise. The reaction mixture was slowly brought to room temperature and stirred for 12 hours. Completion of the reaction was confirmed by LC−MS. The reaction mixture was quenched with aq. ammonium chloride at -20 ^{o}C. The product was extracted with ethyl acetate and then the organic layer was washed with brine solution and dried over anhydrous Na_2SO_4. The solvent was evaporated under reduced pressure and the crude product was purified by flash column chromatography to afford the title compound (4.19 g; 11.48 mmol, 80%)

as a white solid. ^{1}H NMR (400 MHz, DMSO) δ 7.45 – 7.43 (m, 1H), 7.35 – 7.31 (m, 2H), 7.28 – 7.25 (m, 2H), 4.67 – 4.46 (m, 3H), 4.36 (d, J = 9.6 Hz, 2H), 4.17 – 4.08 (m, 2H), 1.40 (s, 9H), 1.22 (t, J = 6.8 Hz, 3H). ^{13}C NMR (100 MHz, DMSO) δ 170.4, 169.0, 155.4, 150.2, 136.1, 127.8, 126.2, 123.6, 123.0, 79.1, 76.3, 61.2, 50.7, 50.0, 28.5, 14.4.
ESI-HRMS (m/z): calculated for $C_{18}H_{25}N_2O_6$ $(M+H)^+$ = 365.1713 found: 365.1705.

Ethyl (S)-2-(3-((tert-butoxycarbonyl)amino)-4-oxo-3,4-dihydrobenzo[b][1,4]oxazepin-5(2H)-yl)acetate (5, AP-8-293 (NN-02-209));

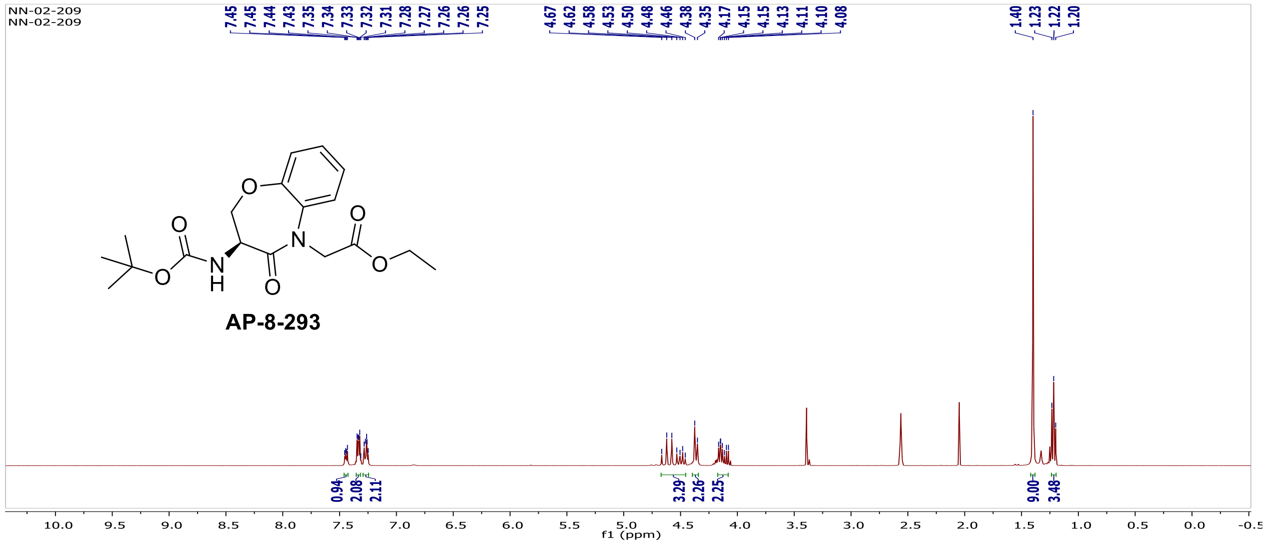

^{1}H NMR of Compound 5, AP-8-293 (NN-02-209)

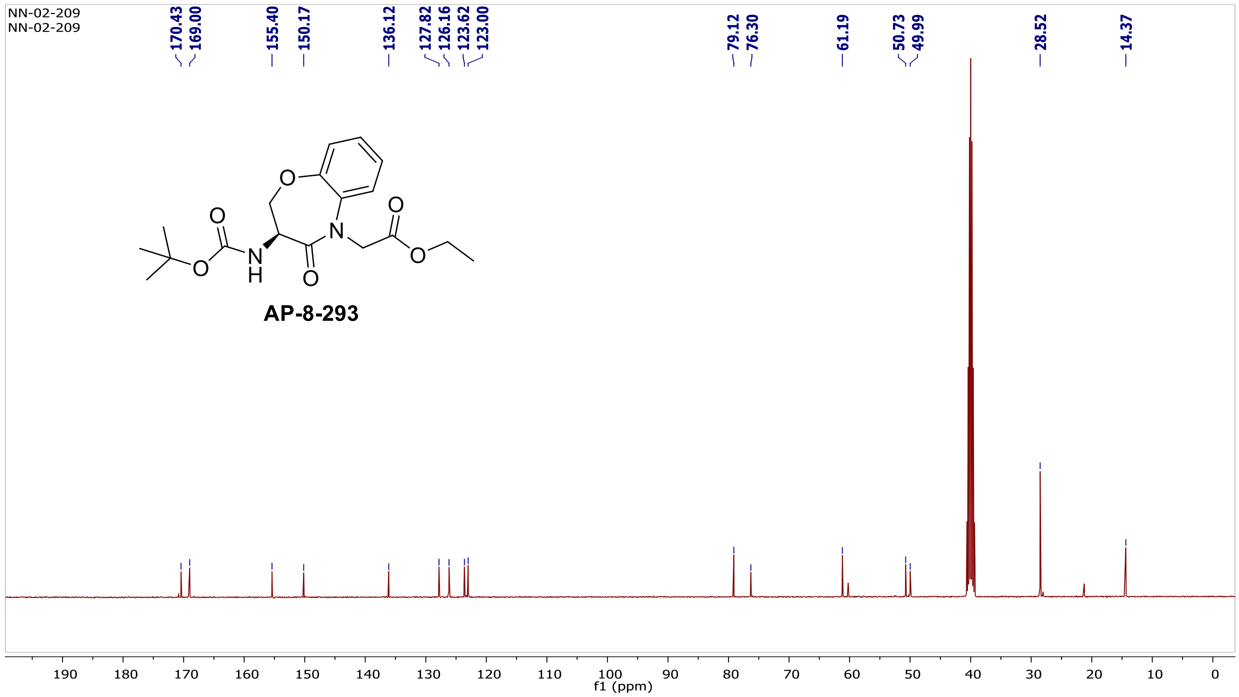

^{13}C NMR of Compound 5, AP-8-293 (NN-02-209)

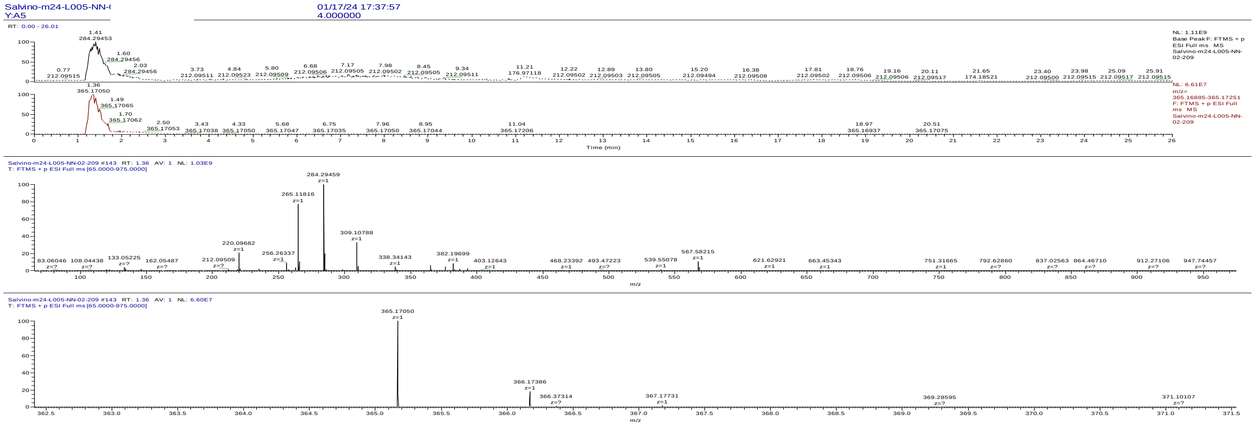

HRMS of Compound 5, AP-8-293 (NN-02-209)

(*S*)-2-(3-((Tert-butoxycarbonyl)amino)-4-oxo-3,4-dihydrobenzo[*b*][1,4]oxazepin-5(2*H*)-yl)acetic acid (6, AP-8-293B); To a solution of (*S*)-ethyl 2-(3-(benzyloxycarbonylamino)-4-oxo-3,4-dihydrobenzo [*b*][1,4] oxazepin-5(2*H*)-yl)acetate (3.0 g; 8.23 mmol) in 60 mL of MeOH, H_2O and THF (4:2:1) at room temperature was added LiOH·H_2O (1.09 mg; 24.70 mmol). The reaction mixture was stirred for 6 hours. Completion of the reaction was confirmed by thin-layer chromatography. Volatiles were evaporated under reduced pressure, and the crude product was acidified with 1 N HCl at 0 °C to obtain a white precipitate which was filtered and dried under a high vacuum to afford the title compound (2.66 g; 7.91 mmol, 96%) as a white solid. ^{1}H NMR (400 MHz, DMSO) δ 12.02 (s, 1H), 7.38 – 7.37 (m, 1H), 7.28 – 7.26 (m, 2H), 7.2 – 7.17 (m, 2H), 4.55 – 4.51 (m, 1H), 4.44 – 4.35 (m, 2H), 4.35 – 4.28 (m, 2H), 1.35 (s, 9H). ^{13}C NMR (100 MHz, DMSO) δ 170.5, 170.2, 155.3, 150.1, 136.3, 127.7, 126.1, 123.6, 122.9, 79.1, 76.4, 50.7, 49.9, 28.5.

ESI-HRMS (m/z): calculated for $C_{16}H_{21}N_2O_6$ $(M+H)^+$ = 337.1400 found: 337.1387

(S)-2-(3-((Tert-butoxycarbonyl)amino)-4-oxo-3,4-dihydrobenzo[b][1,4]oxazepin-5(2H)-yl)acetic acid (6, AP-8-293B);

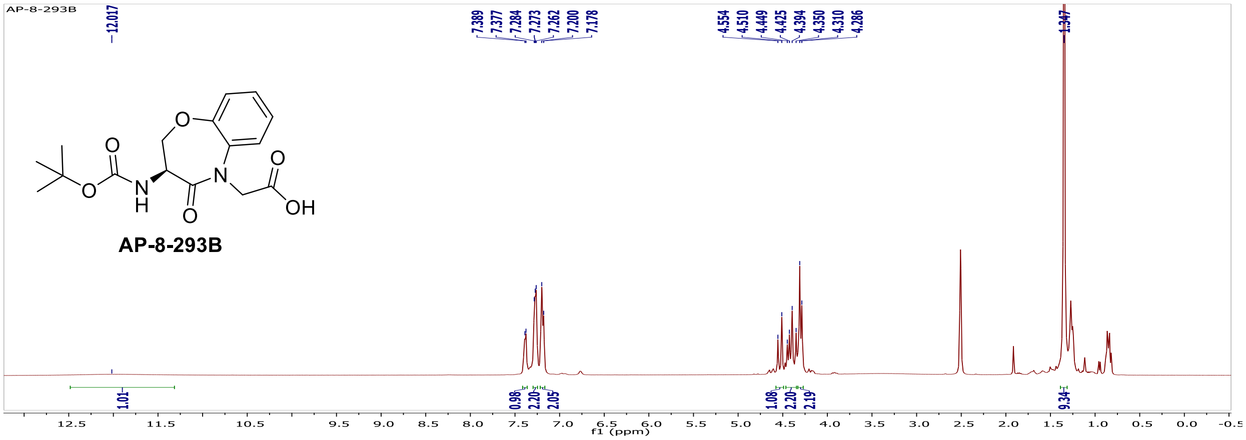

^{1}H NMR of Compound 6, AP-8-293B

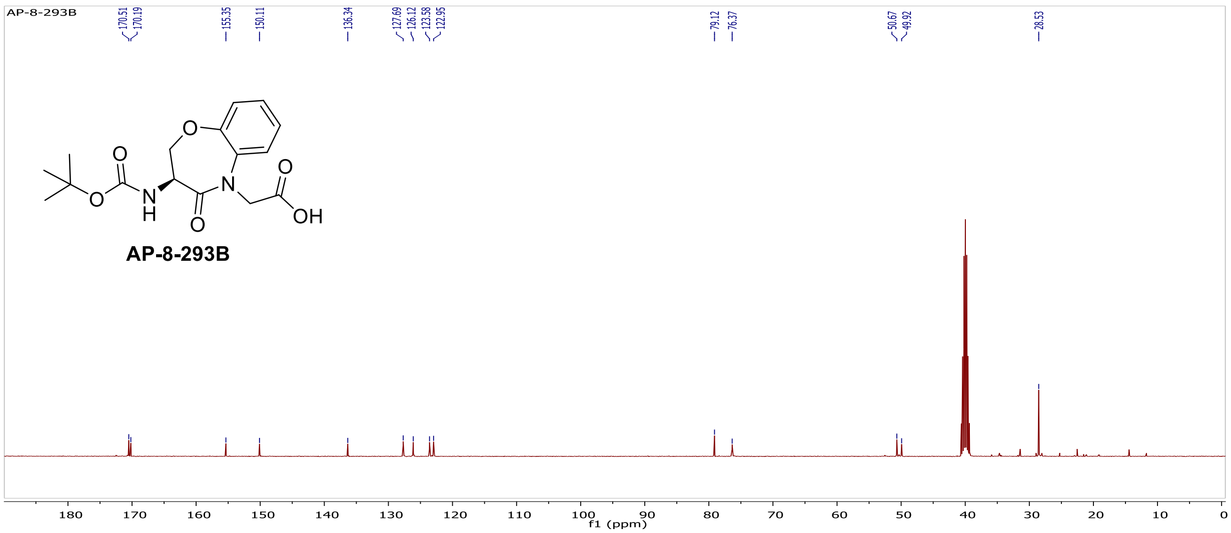

^{13}C NMR of Compound 6, AP-8-293B

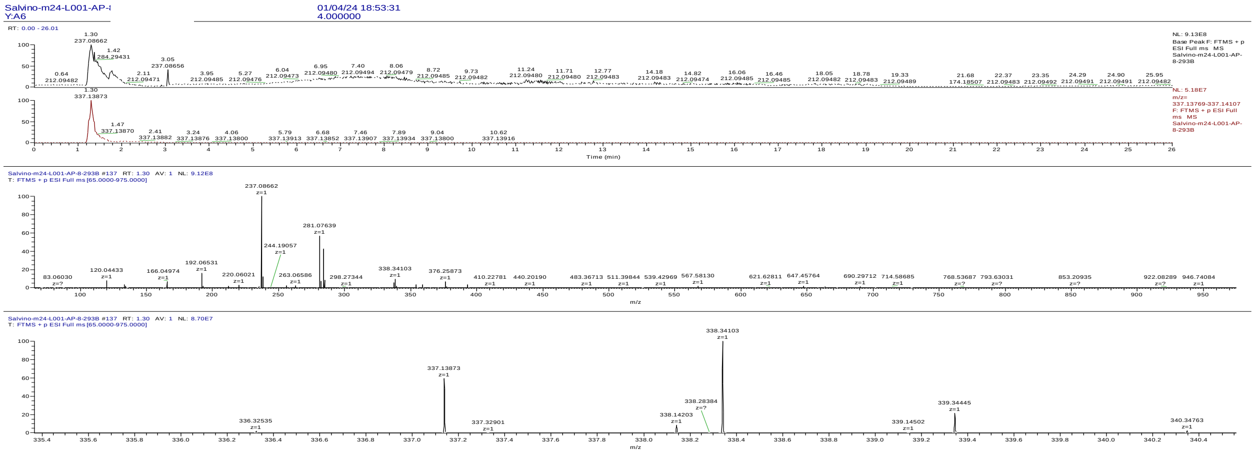

HRMS of Compound AP-8-293B

(*S*)-3-(2-((*S*)-3-(Tert-butoxycarbonylamino)-4-oxo-3,4-dihydrobenzo[*b*][1,4]oxazepin-5(2*H*)-yl)acetamido)-2-oxo-4-((*S*)-2-oxopyrrolidin-3-yl)butyl 2,6-dichlorobenzoate (8, AP-9-22B); To a stirred solution of (*S*)-2-(3-(tert-butoxycarbonylamino)-4-oxo-3,4-dihydrobenzo [*b*][1,4]oxazepin-5(2*H*)-yl)acetic acid (500 mg; 1.48 mmol) and (*S*)-3-amino-2-oxo-4-((*S*)-2-oxopyrrolidin-3-yl)butyl 2,6-dichlorobenzoate 2,2,2-trifluoroacetate (588 mg; 1.48 mmol) in 20 mL dry CH_2Cl_2 at 0 °C was added diisopropyl ethyl amine (1.03 mL, 5.9 mmol) and T3P 50% solution in CH_2Cl_2 by weight (1.42 g; 2.23 mmol) dropwise simultaneously. The reaction mixture was stirred for an additional 1 hour at 0 °C, and completion of the reaction was confirmed by LC−MS. The reaction mixture was quenched with cold water and the product was extracted with CH_2Cl_2 and dried over anhydrous Na_2SO_4. The solvent was removed under reduced pressure to provide the crude reaction product, which was purified by flash column chromatography to afford the title compound as a white solid (0.802 g, 1.18 mmol, 80%). ^{1}H NMR (400 MHz, DMSO) δ 8.72 (d, *J* = 7.7 Hz, 1H), 7.68 (s, 1H), 7.64 – 7.60 (m, 2H), 7.58 – 7.56 (m, 1H), 7.44 – 7.42 (m, 1H), 7.27 – 7.26 (m, 2H), 7.20 – 7.18 (m, 1H), 7.13 (d, *J* = 8.7 Hz, 1H), 5.22 – 5.03 (m, 2H), 4.53

– 4.41 (m, 2H), 3.22 – 3.13 (m, 2H), 2.89 (s, 2H), 2.74 (s, 2H), 2.31 – 2.24 (m, 1H), 2.15 – 2.08 (m, 1H), 2.05 – 1.98 (m, 1H), 1.72 – 1.63 (m, 2H), 1.32 (s, 9H). ^{13}C NMR (100 MHz, DMSO) δ 201.7, 178.6, 170.2, 168.9, 163.7, 162.8, 155.3, 150.1, 136.5, 133.0, 132.5, 131.3, 129.0, 127.7, 126.0, 123.9, 122.9, 79.1, 76.4, 68.2, 54.7, 51.2, 50.7, 37.6, 36.2, 31.5, 31.2, 28.5.
ESI-HRMS (m/z): calculated for $C_{31}H_{35}Cl_2N_4O_9$ $(M+H)^+$ = 677.1781 found: 677.1766

(S)-3-(2-((S)-3-(Tert-butoxycarbonylamino)-4-oxo-3,4-dihydrobenzo[b][1,4]oxazepin-5(2H)-yl)acetamido)-2-oxo-4-((S)-2-oxopyrrolidin-3-yl)butyl 2,6-dichlorobenzoate (8, AP-9-22B);

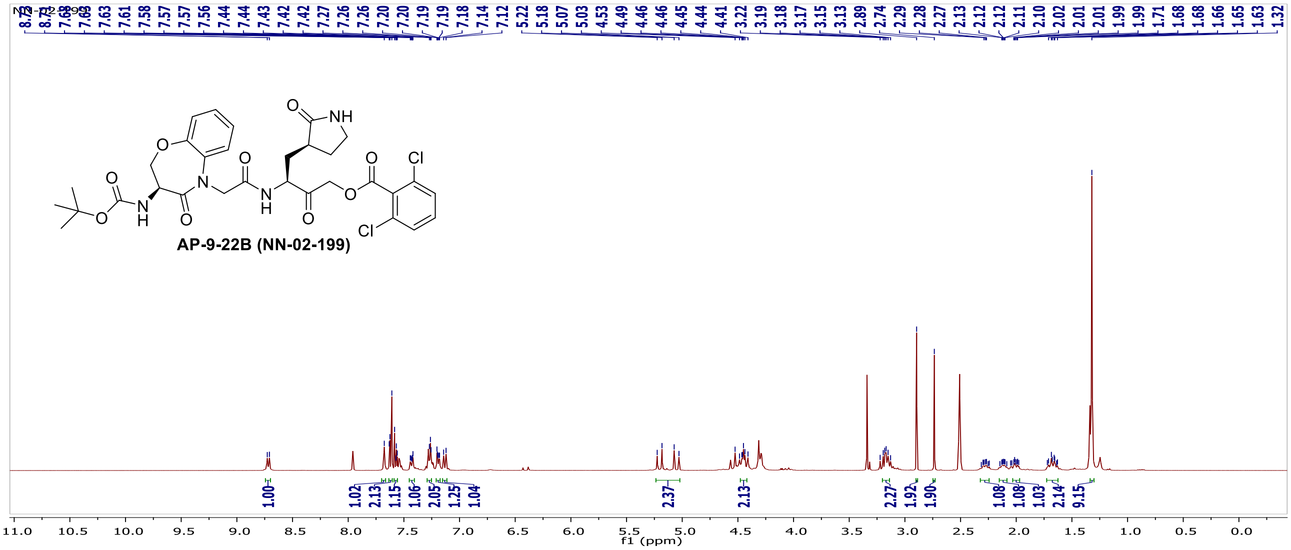

^{1}H NMR of Compound 8, AP-9-22B

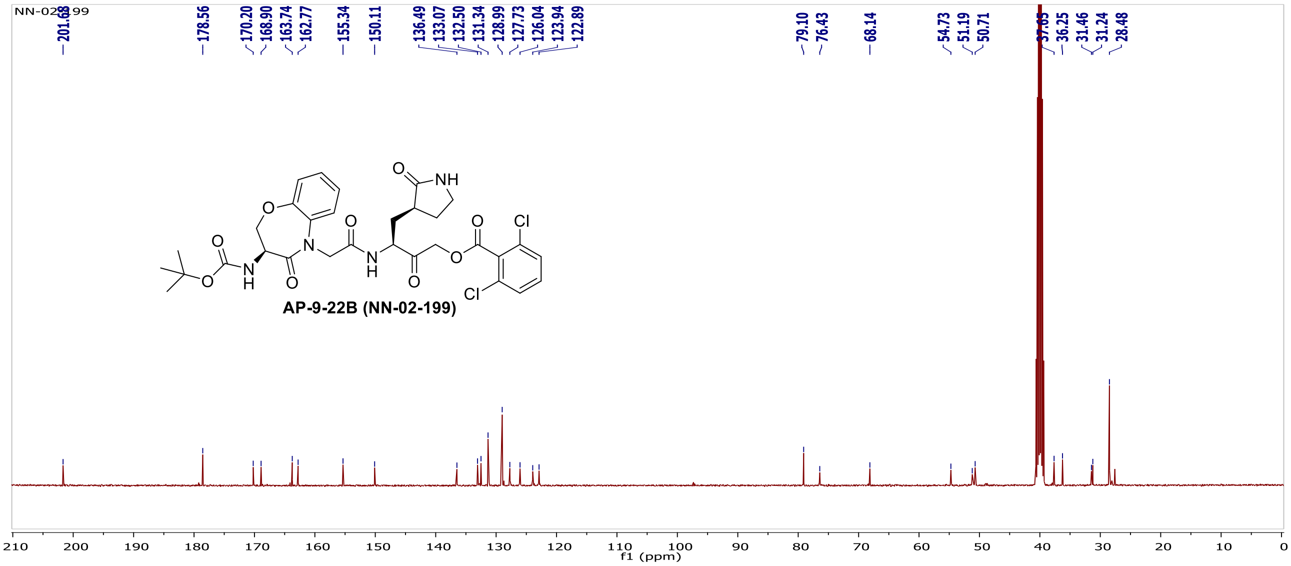

^{13}C NMR of Compound 8, AP-9-22B

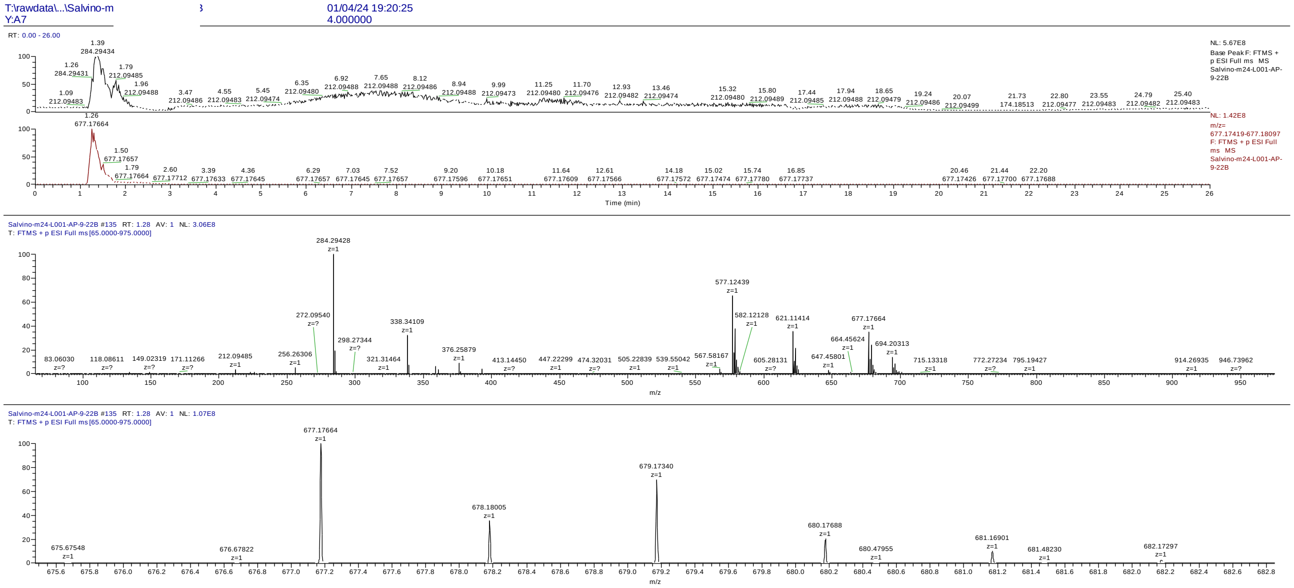

HRMS of Compound 8, AP-9-22B

(*S*)-3-(2-((*S*)-3-Amino-4-oxo-3,4-dihydrobenzo[*b*][1,4]oxazepin-5(2*H*)-yl)acetamido)-2-oxo-4-((*S*)-2-oxopyrrolidin-3-yl)butyl 2,6-dichlorobenzoate (9, NN-02-200); To a stirred solution of (*S*)-3-(2-((*S*)-3-(tert-butoxycarbonylamino)-4-oxo-3,4-dihydrobenzo [*b*][1,4]oxazepin-5(2*H*)-yl)acetamido)-2-oxo-4-((*S*)-2-oxopyrrolidin-3-yl)butyl 2,6-dichloro benzoate (300 mg, 0.44 mmol) in 8 mL of CH_2Cl_2 at 0 °C was added 2 mL of TFA. The reaction mixture was stirred at room temperature for 2 hours. Completion of the reaction was confirmed by LC−MS. The solvent was removed under reduced pressure to provide the crude reaction product, which was purified by flash column chromatography to afford the title compound as a white solid (298 mg, 0.44 mmol, 100%). ^{1}H NMR (400 MHz, DMSO) δ 8.88 (d, J = 7.5 Hz, 1H), 8.46 (s, 2H), 7.74 (s, 1H), 7.64 – 7.62 (m, 2H), 7.59 – 7.57 (m, 1H), 7.44 – 7.42 (m 1H), 7.34 – 7.31 (m, 2H), 7.29 – 7.26 (m, 1H), 5.28 - 5.14 (m, 2H), 4.74 – 4.69 (m, 1H), 4.46 – 4.37 (m, 3H), 3.20 – 3.15 (m, 2H), 2.89 (s, 1H), 2.74 (s, 1H), 2.38 – 2.31 (m, 1H), 2.20 – 2.14 (m, 1H), 2.06 – 1.99 (m, 1H), 1.77 – 1.68 (m, 2H). ^{13}C NMR (100 MHz, DMSO) δ 201.7, 178.7, 168.5, 167.4, 163.8, 149.8, 135.5, 133.1, 132.5, 131.3, 129.0, 128.3, 126.6, 124.2, 123.0, 118.1, 115.2, 75.1, 68.2, 54.9, 51.50, 49.8, 37.8, 31.5, 27.6.

ESI-HRMS (m/z): calculated for $C_{26}H_{27}Cl_2N_4O_7$ $(M+H)^+$ = 577.1257 found: 577.1242

(S)-3-(2-((S)-3-Amino-4-oxo-3,4-dihydrobenzo[b][1,4]oxazepin-5(2H)-yl)acetamido)-2-oxo-4-((S)-2-oxopyrrolidin-3-yl)butyl 2,6-dichlorobenzoate (9, AP-9-055 (NN-02-200);

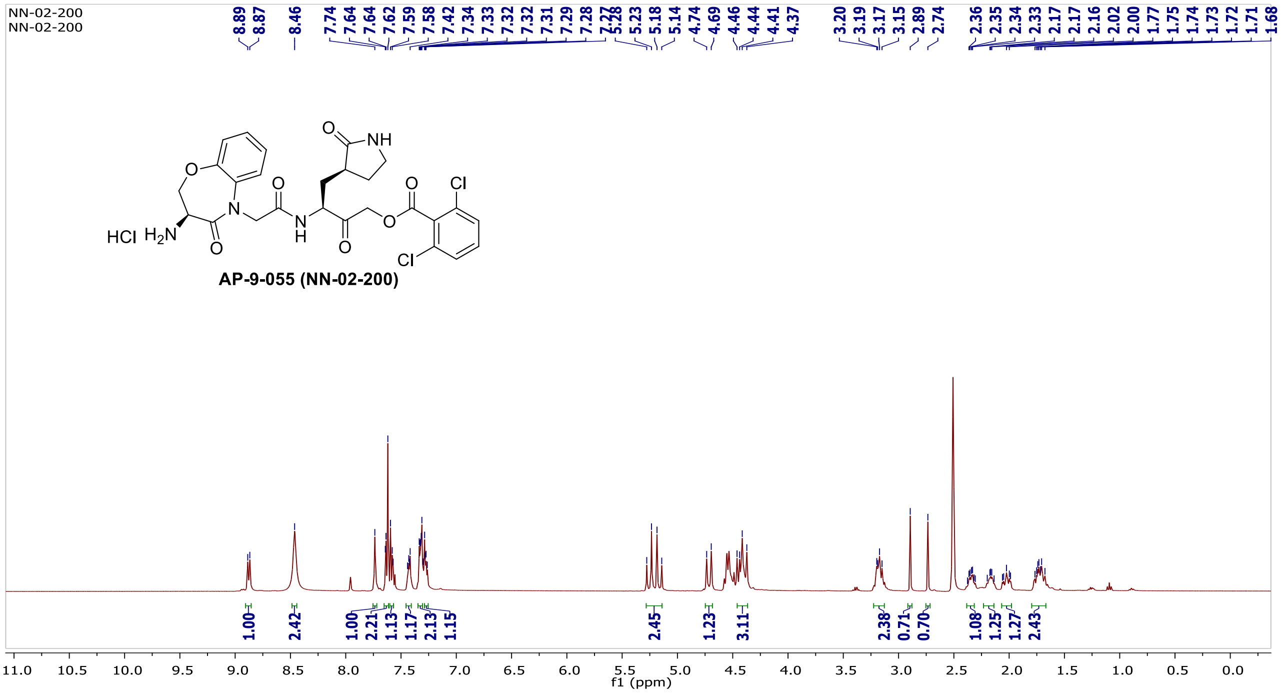

^{1}H NMR of Compound 9, AP-9-055 (NN-02-200)

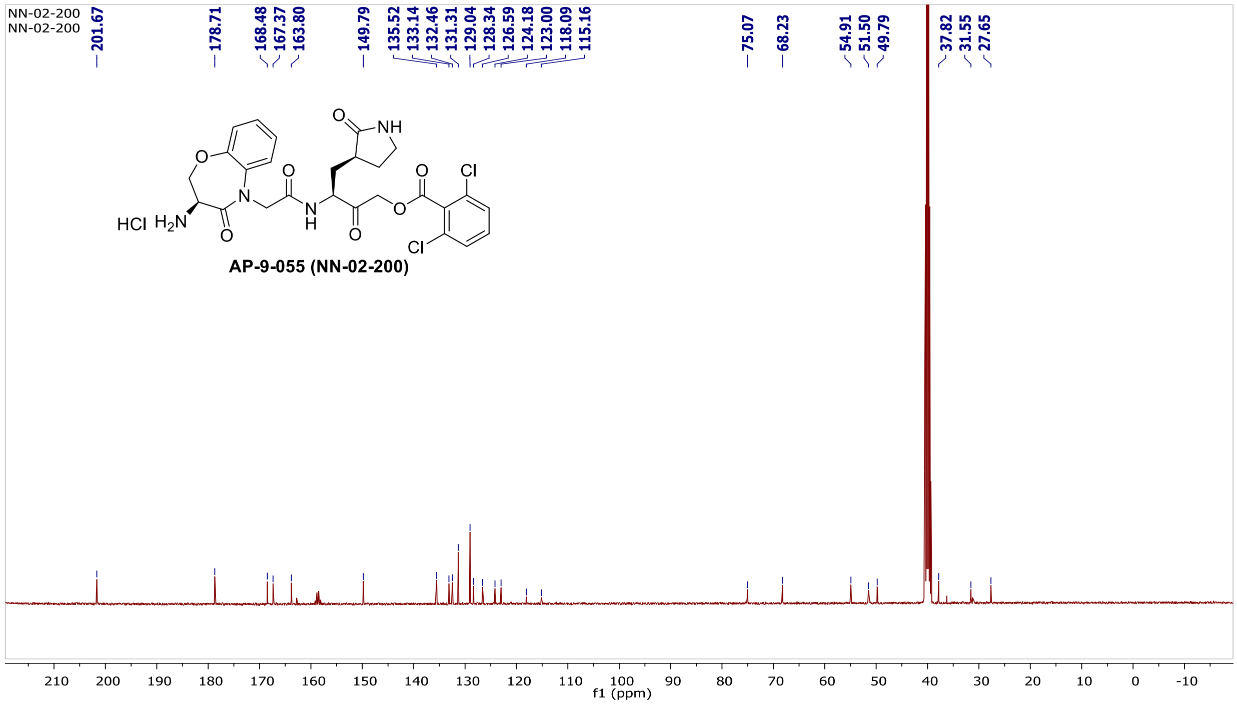

^{13}C NMR of Compound 9, AP-9-055 (NN-02-200)

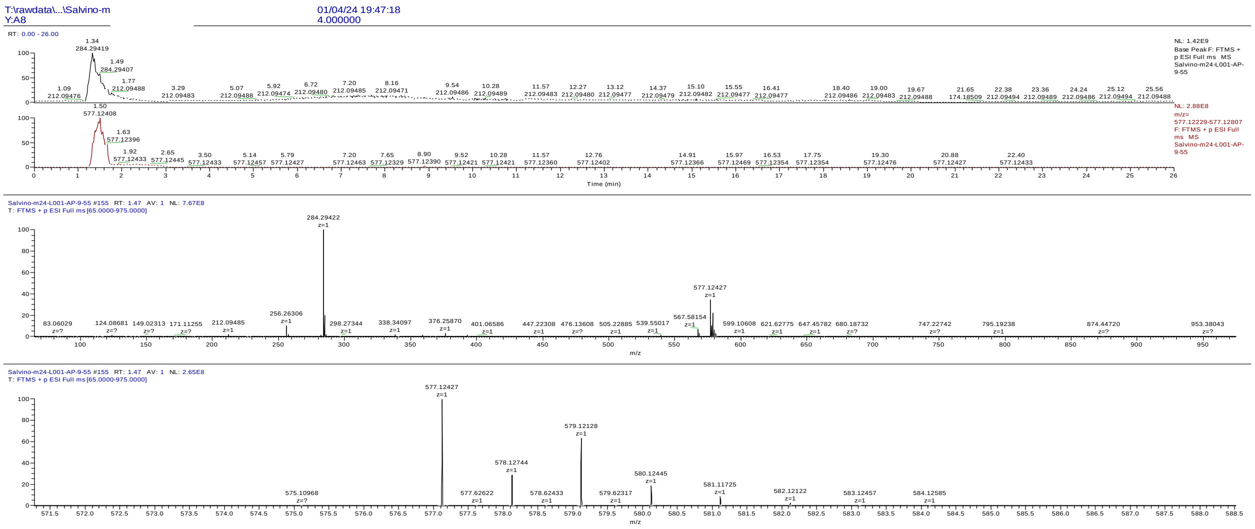

HRMS of Compound 9, AP-9-055 (NN-02-200)

(*S*)-3-(2-((*S*)-3-(Benzyloxycarbonylamino)-4-oxo-3,4-dihydrobenzo[*b*][1,4]oxazepin-5(2*H*)-yl)acetamido)-2-oxo-4-((*S*)-2-oxopyrrolidin-3-yl)butyl 2,6-dichlorobenzoate (1, AP-8-013); To a stirred solution of (*S*)-3-(2-((*S*)-3-amino-4-oxo-3,4-dihydrobenzo[*b*][1,4]oxazepin-5(2*H*)-yl) acetamido)-2-oxo-4-((*S*)-2-oxopyrrolidin-3-yl)butyl 2,6-dichlorobenzoate 2,2,2-trifluoroacetate (323 mg; 0.47 mmol) in 10 mL of dry CH_2Cl_2 at 0 °C was added triethylamine (0.24 mL; 2.3 mmol) and benzyl chloroformate (88 mg, 0.51 mmol) dropwise simultaneously. The reaction mixture was allowed to stir for 12 hours at 0 °C. Completion of the reaction was confirmed by LC−MS. The reaction mixture was quenched with cold water, extracted with CH_2Cl_2, and dried over anhydrous Na_2SO_4. The solvent was evaporated under reduced pressure, and the crude product was purified by reverse phase HPLC to afford the title compound (150 mg, 0.31 mmol, 65%) as a white solid. ^{1}H NMR (400 MHz, DMSO) δ 8.71 (d, J = 7.7 Hz, 1H), 7.69 – 7.67 (m, 2H), 7.62 – 7.54 (m, 3H), 7.45 – 7.42 (m, 1H), 7.34 – 7.26 (m, 7H), 7.22 – 7.19 (m, 1H), 5.24 – 5.06 (m, 2H), 4.99 – 4.97 (m, 2H), 4.56 – 4.49 (m, 3H), 4.44 – 4.31 (m, 3H), 3.20 – 3.10 (m, 2H), 2.34 – 2.24 (m, 1H), 2.16 – 1.98 (m, 2H), 1.74 – 1.63 (m, 2H). ^{13}C NMR (100 MHz, DMSO) δ 201.7, 178.6, 170.0, 168.8, 163.8, 156.1, 150.1, 137.1, 136.4, 133.1, 132.5, 131.3, 129.0, 128.8, 128.3, 128.2, 127.8, 126.1, 123.9, 122.9, 76.4, 68.2, 66.3, 54.7, 51.3, 51.2, 37.7, 31.5, 27.6.
ESI-HRMS (m/z): calculated for $C_{34}H_{33}Cl_2N_4O_9$ $(M+H)^+$ = 711.1625 found: 711.1608.
ESI-LCMS (m/z): 711.36 $(M+H)^+$.

(S)-3-(2-((S)-3-(Benzyloxycarbonylamino)-4-oxo-3,4-dihydrobenzo[b][1,4]oxazepin-5(2H)-yl)acetamido)-2-oxo-4-((S)-2-oxopyrrolidin-3-yl)butyl 2,6-dichlorobenzoate (1, AP-8-013);

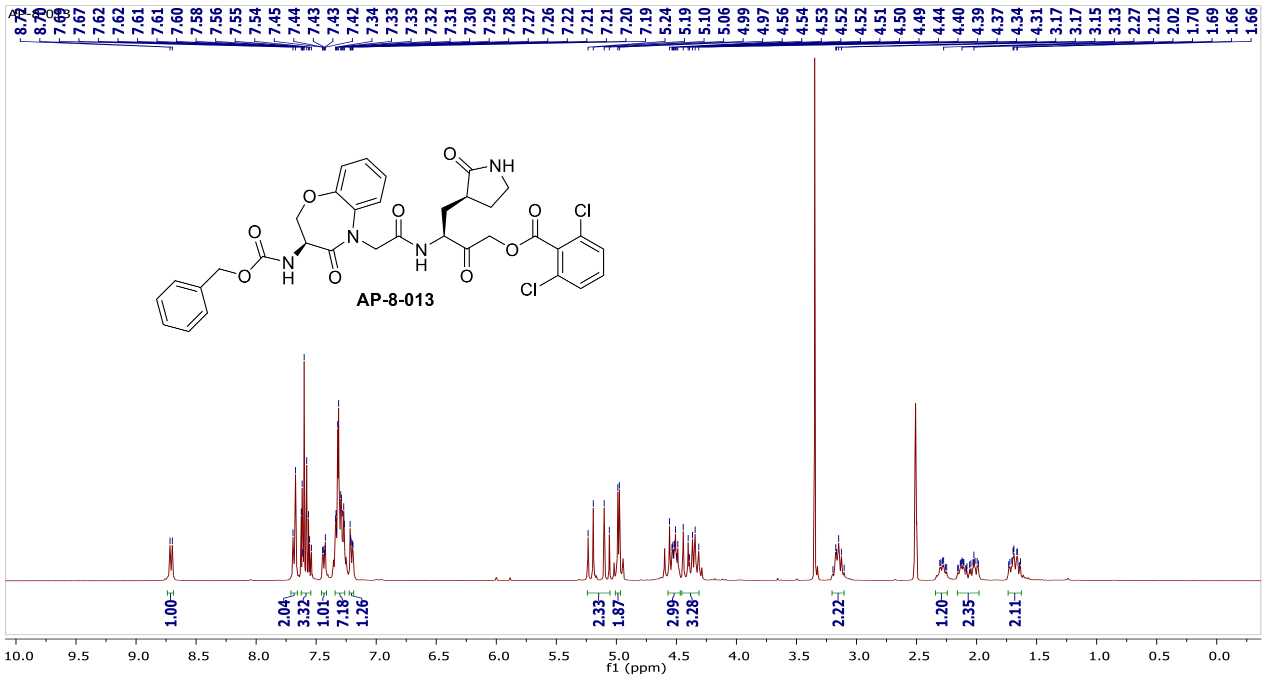

^{1}H NMR of Compound 1, AP-8-013

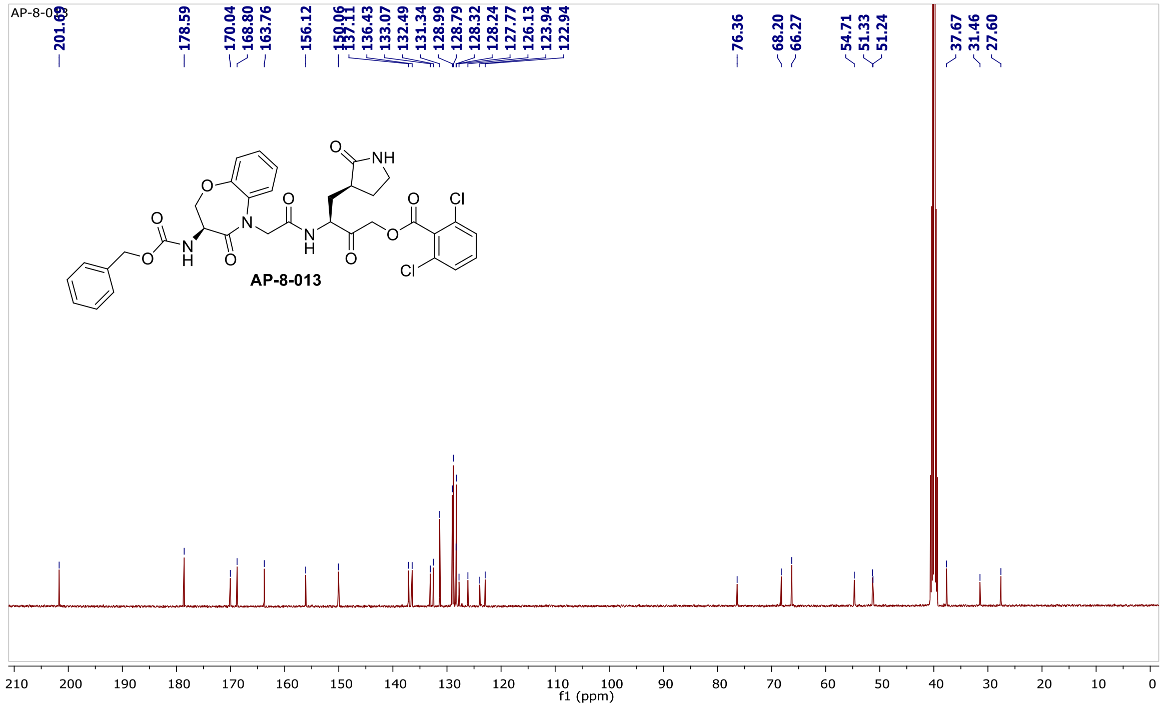

^{13}C NMR of Compound 1, AP-8-013

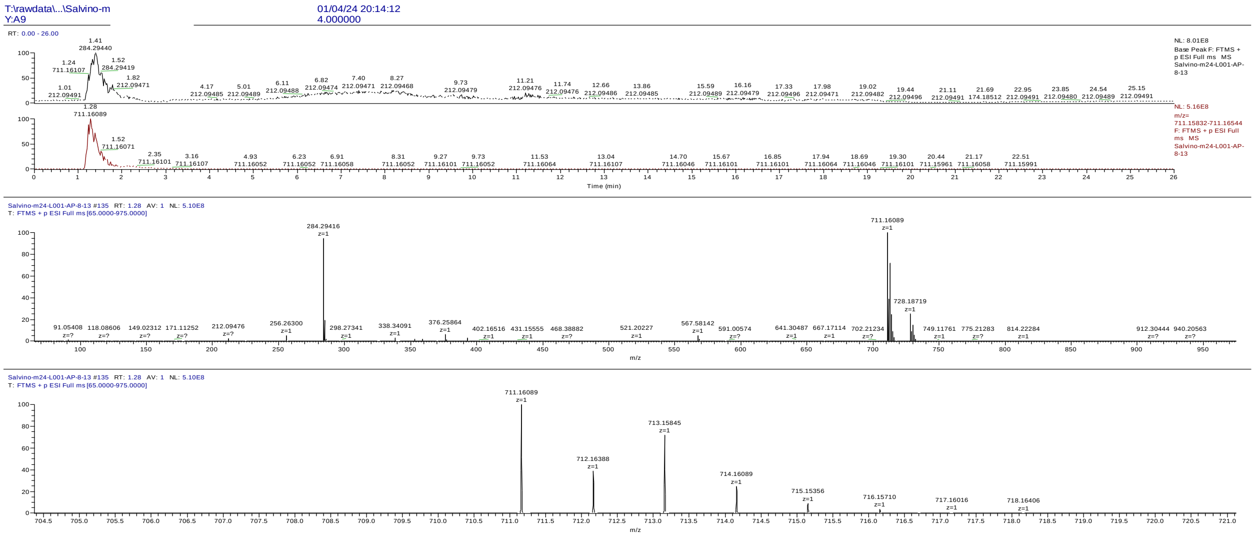

HRMS of Compound 1, AP-8-013

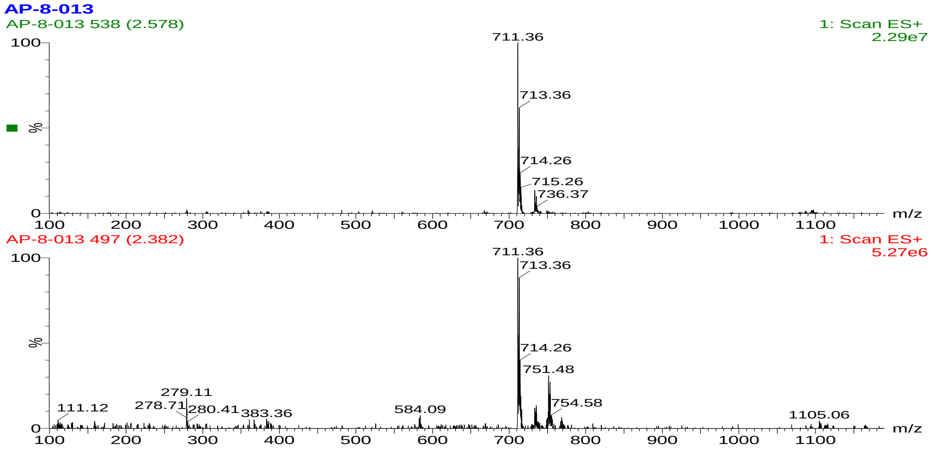

HRMS of Compound 1, AP-8-013

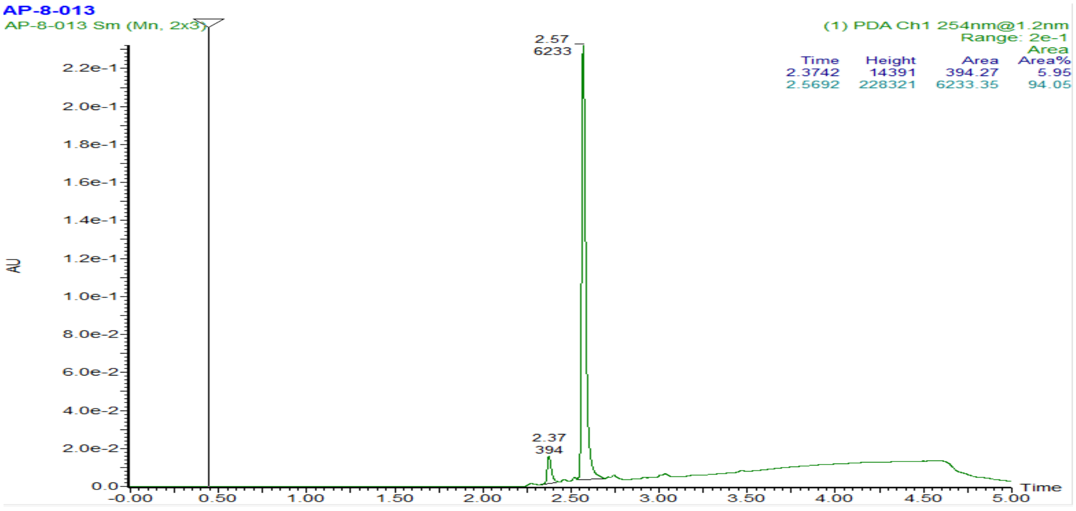

LCMS of Compound 1, AP-8-013

Figure 5. Synthesis of Compound 7

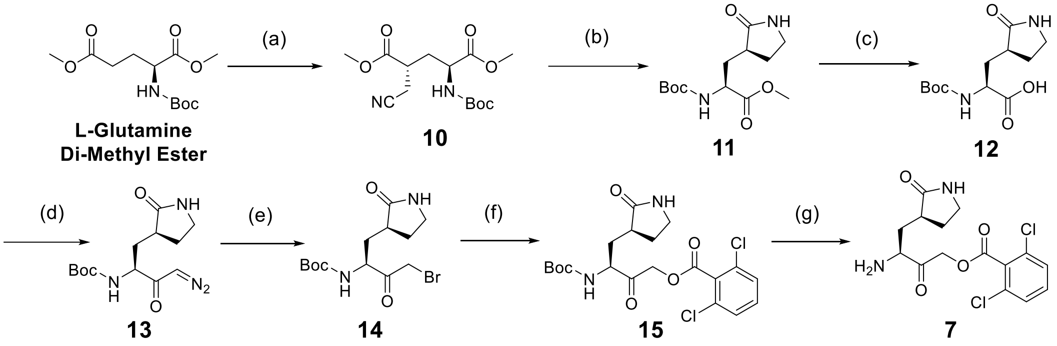

(2*S*,4*R*)-Dimethyl 2-(tert-butoxycarbonylamino)-4-(cyanomethyl)pentanedioate (10, AP-7-267); To a stirred solution of *N*-Boc-L-glutamic acid dimethyl ester (20 g, 72.64 mmol) in THF (200 mL) was added dropwise a solution of lithium bis(trimethylsilyl)amide (LHMDS) in THF (160 mL, 159.82 mmol, 1 M) at −78°C under a nitrogen atmosphere. The resulting reaction mixture was stirred at −78°C. Then bromo acetonitrile (13.06 g, 108.96 mmol) was added dropwise over a period of 1 hour at −78°C. The reaction mixture was stirred at −78°C for an additional 2 hours. After the consumption of the reactant was confirmed by TLC analysis, the reaction was quenched by methanol (10 mL), and acetic acid (10 mL) in pre-cooled THF (30 mL) was added. After stirring for 30 min, the cooling bath was removed. The reaction mixture was allowed to warm up to room temperature and then poured into brine (80 mL). The organic layer was concentrated under reduced pressure and purified by flash column chromatography to give the desired product (16.44 g, 52.3 mmol, 72%) as a colorless oil. ^{1}H NMR (400 MHz, $CDCl_3$) δ 5.11 (d, J = 7.7 Hz, 1H), 4.39 (d, J = 4.6 Hz, 1H), 3.77 – 3.76 (m, 6H), 2.88 – 2.79 (m, 3H), 2.19 – 2.13 (m, 2H), 1.45 (s, 9H).
ESI-MS (m/z): calculated for $C_{14}H_{22}N_2NaO_6$ $(M+Na)^+$ = 337.13; found: 337.00.

(2S,4R)-Dimethyl 2-(tert-butoxycarbonylamino)-4-(cyanomethyl)pentanedioate (10, AP-7-267);

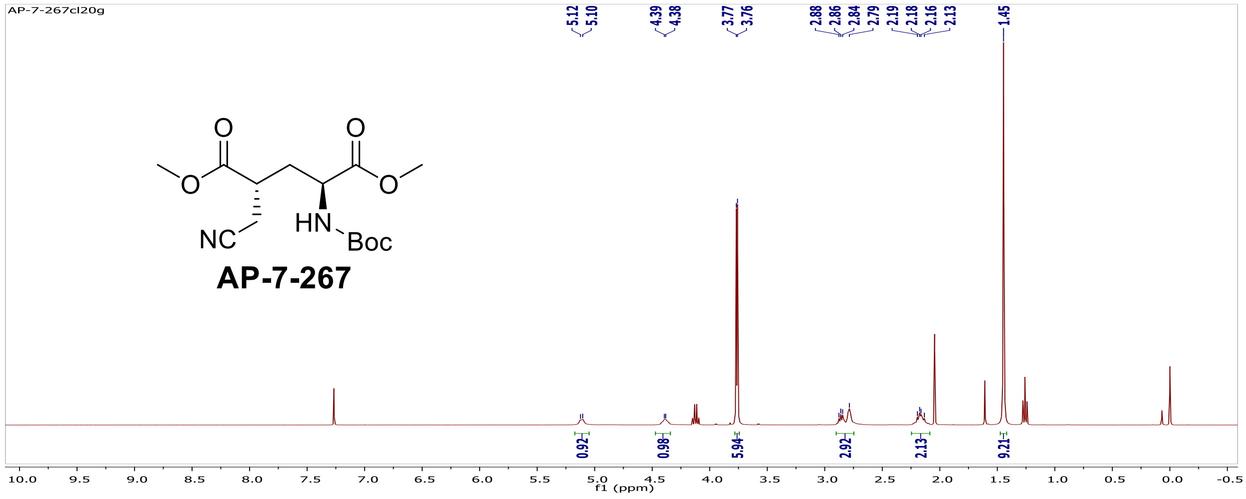

^{1}H NMR of Compound AP-7-267

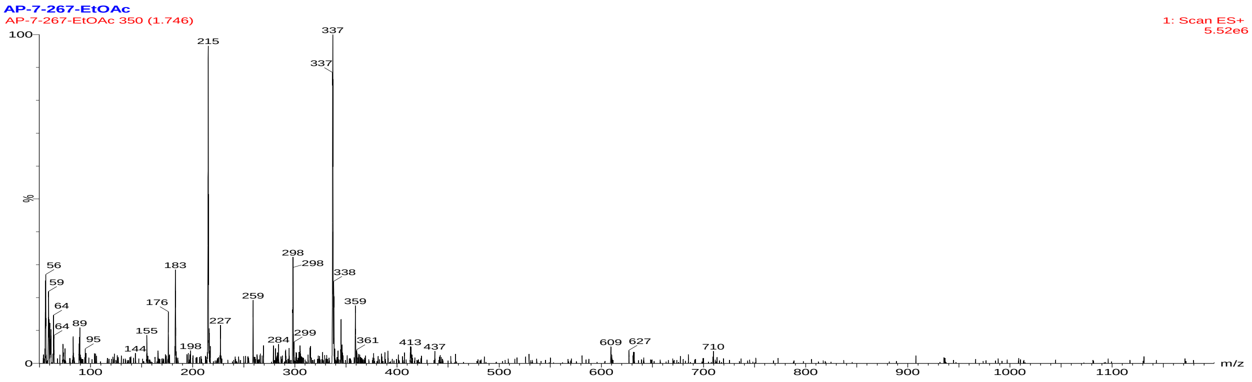

LCMS of Compound AP-7-267

(*S*)-Methyl 2-(tert-butoxycarbonylamino)-3-((*S*)-2-oxopyrrolidin-3-yl)propanoate (11, AP-7-268); In a hydrogenation flask was placed compound **AP-7-267** (12 g, 38.17 mmol), 60 mL of chloroform, and 240 mL of methanol before the addition of PtO_2. The resulting mixture was stirred under hydrogen at 20 °C for 12 hours. Then, the mixture was filtered over celite to remove the catalyst. NaOAc (6.77 g, 25.5mmol) was added to the filtrate before the mixture was stirred at 60°C for 12 hours. The reaction was quenched with water (30 mL). The suspension was extracted with ethyl acetate. The organic layers were combined, dried (MgSO4), and filtered. The light-brown filtrate was concentrated and purified by silica gel column chromatography to give the target product **AP-7-268** (6.67 g, 23.28 mmol, 61%) as a white solid. ^{1}H NMR (400 MHz, DMSO) δ 7.65 (s, 1H), 7.42 (d, J = 8.0 Hz, 1H), 4.06 – 4.00 (m, 1H), 3.63 (s, 3H), 3.16 – 3.12 (m, 2H), 2.31 – 2.23 (m, 1H), 2.17 – 2.13 (m, 1H), 2.02 – 1.95 (m, 1H), 1.65 – 1.53 (m, 2H), 1.38 (s, 9H). ^{13}C NMR (100 MHz, DMSO) δ 178.5, 173.4, 156.0, 78.7, 52.4, 52.3, 38.2, 32.7, 28.6, 27.7.
ESI-HRMS (m/z): calculated for $C_{13}H_{23}N_2O_5$ $(M+H)^+$ = 287.1607; found: 287.1595.

(S)-Methyl 2-(tert-butoxycarbonylamino)-3-((S)-2-oxopyrrolidin-3-yl)propanoate (11, AP-7-268);

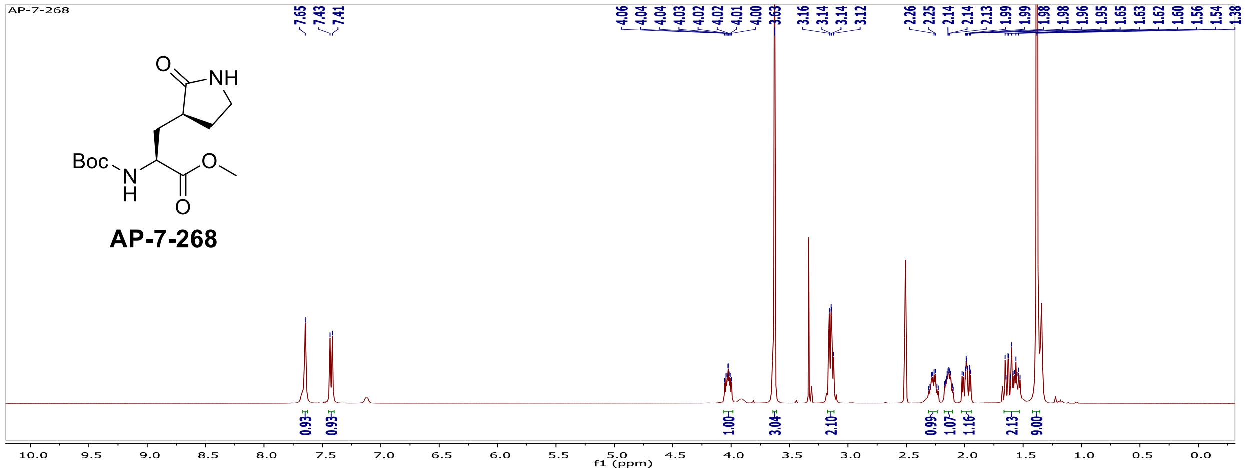

^{1}H NMR of Compound 11, AP-7-268

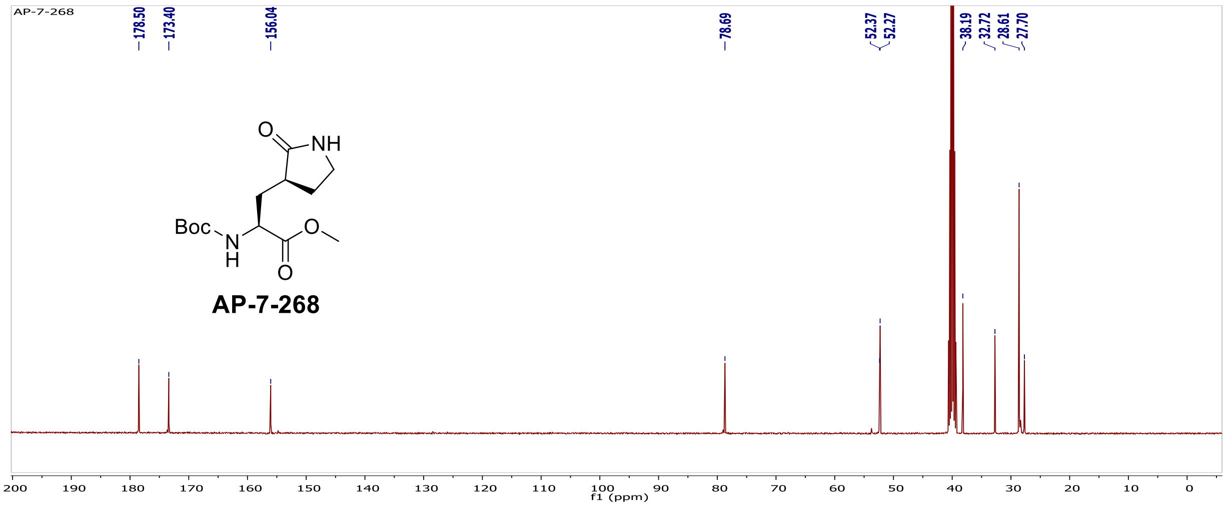

^{13}C NMR of Compound 11, AP-7-268

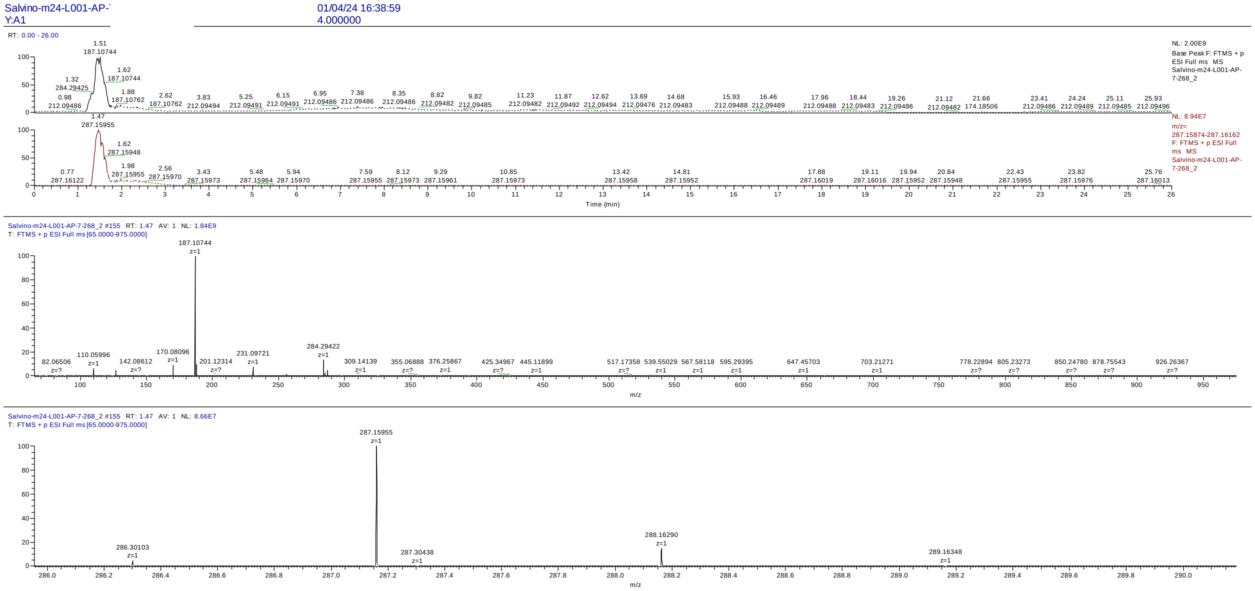

HRMS of Compound 11, AP-7-268

(*S*)-2-(Tert-butoxycarbonylamino)-3-((*S*)-2-oxopyrrolidin-3-yl)propanoic acid (12, AP-7-270 (NN-02-202)); To a solution (*S*)-methyl 2-(tert-butoxycarbonylamino)-3-((*S*)-2-oxopyrrolidin-3-yl) propanoate (**AP-7-268**) purchased from Aaron chemicals (10 g; 34.92 mmol) in 180 mL of MeOH, H_2O and THF (5:5:1) at room temperature was added LiOH·H_2O (4.6 mg; 104.76 mmol). The reaction mixture was stirred for 6 hours. Completion of the reaction was confirmed by thin-layer chromatography. Volatiles were evaporated by reduced pressure, and the crude product was acidified with 1 N HCl at 0 °C and saturated with NaCl. Then the product extracted with 20% IPA in CH_2Cl_2 (100 mL x 4), dried over Na_2SO_4, filtered, and concentrated to afford the title compound (9.03 g; 33.17 mmol, 95%) as a white solid. ^{1}H NMR (400 MHz, DMSO) δ 12.50 (s, 1H), 7.64 (s, 1H), 7.25 (d, J = 8.2 Hz, 1H), 3.96 – 3.90 (m, 1H), 3.16 – 3.12 (m, 2H), 2.30 – 2.22 (m, 1H), 2.19

– 2.11 (m, 1H), 1.97 – 1.93 (m, 1H), 1.68 – 1.52 (m, 2H), 1.38 (s, 9H). ^{13}C NMR (100 MHz, DMSO) δ 178.7, 174.5, 156.1, 78.5, 52.3, 38.3, 32.8, 28.6, 27.8.
ESI-HRMS (m/z): calculated for $C_{12}H_{21}N_2O_5$ $(M+H)^+$ = 273.1450; found: 273.1440.

(*S*)-2-(Tert-butoxycarbonylamino)-3-((*S*)-2-oxopyrrolidin-3-yl)propanoic acid (12, AP-7-270 (NN-02-202));

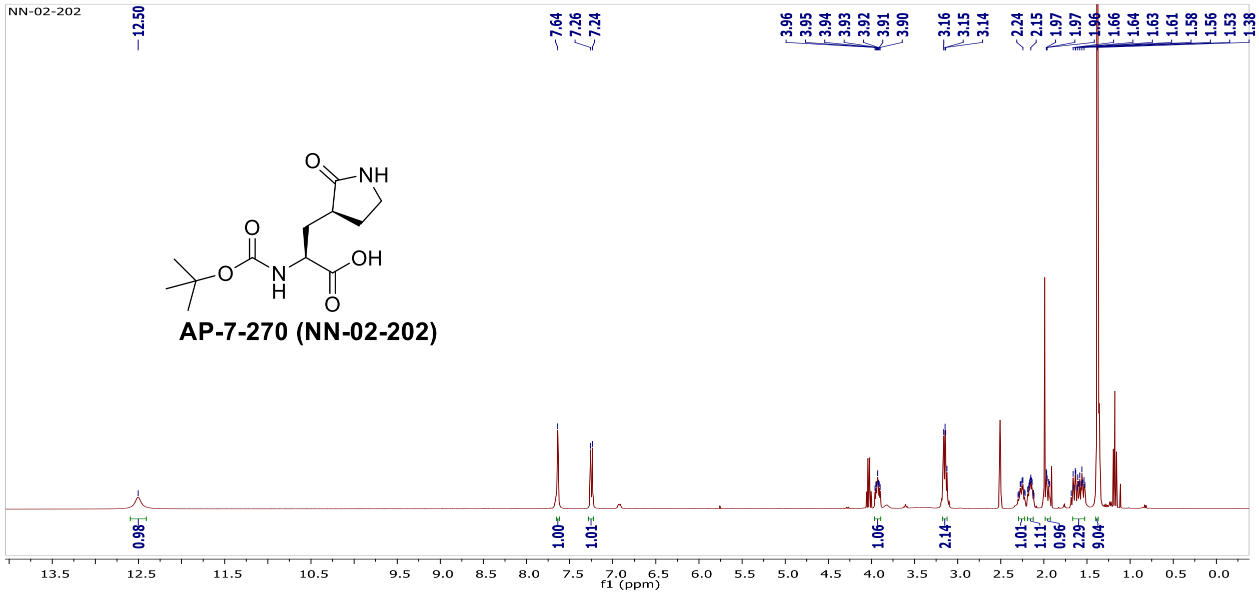

^{1}H NMR of Compound 12, AP-7-270 (NN-02-202)

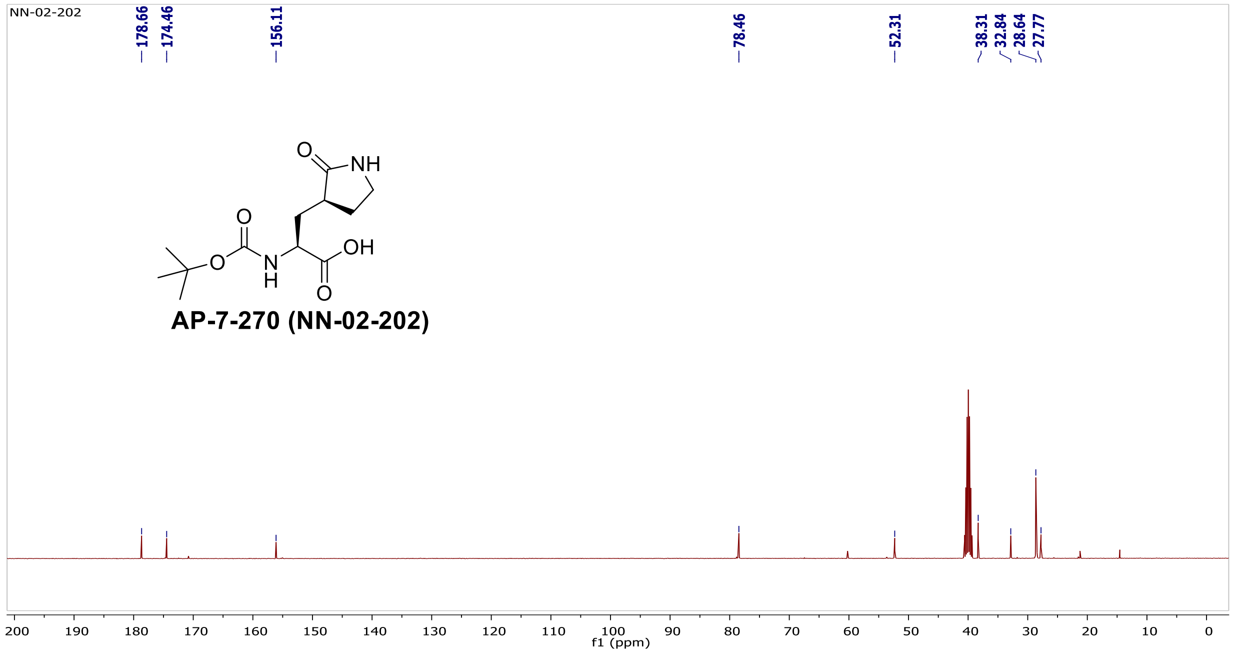

^{13}C NMR of Compound AP-7-270

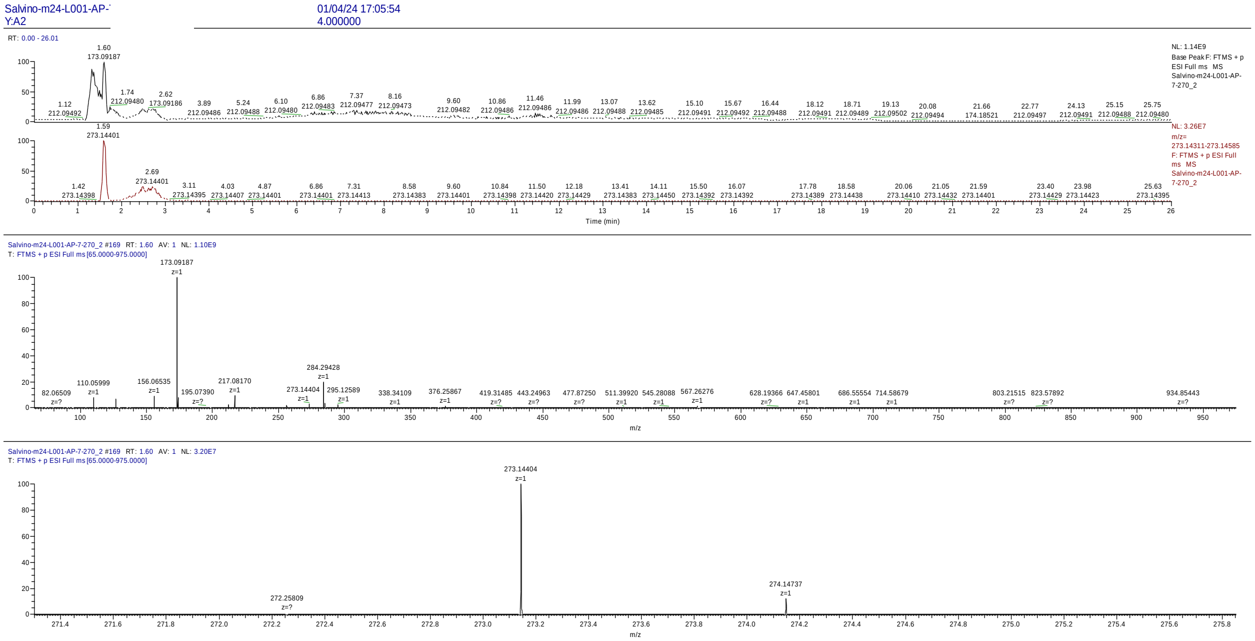

HRMS of Compound AP-7-270

Tert-butyl (*S*)-4-diazo-3-oxo-1-((*S*)-2-oxopyrrolidin-3-yl)butan-2-ylcarbamate (13, AP-8-260 (AP-7-273)); To a stirred solution of *N*-(tert-butoxycarbonyl)-3-[(3*S*)-2-oxopyrrolidin-3-yl]-L-alanine (7.833 g, 28.76 mmol) in THF (200 mL) was placed under an atmosphere of N_2 and cooled to −20 °C. The resulting clear colorless solution was successively treated with triethylamine (6.1 mL, 34.51 mmol) followed by isobutylchloroformate (4.5 mL, 12.0 mmol). The reaction mixture gradually became opaque with a fine white precipitate and after 1 hour was filtered. The colorless filtrate was transferred to a nonground joint flask, cooled to 0 °C, and slowly treated with a solution of diazomethane (~35 mL, ~16.6 mmol) in diethyl ether. Note: The diazomethane was generated employing a Diazald kit according to the procedure described in the Aldrich Technical Bulletin AL-180. The yellow clear solution was gradually warmed to room temperature over 16 hours. At this time, N_2 gas was bubbled through the reaction to remove excess diazomethane followed by *in vacuo* concentration. The resulting residue was diluted with ethyl acetate (200 mL), washed once with saturated $NaHCO_3$ (100 mL), once with brine (100 mL), dried over Na_2SO_4, filtered, and concentrated under reduced pressure and the crude product was purified by flash column chromatography to afford the title compound (7.67 g; 25.9 mmol, 90%). ^{1}H NMR (400 MHz, $CDCl_3$) δ 6.13 (s, 1H), 5.99 (s, 1H), 5.63 (s, 1H), 4.23 (s, 1H), 3.37 – 3.33 (m, 2H), 2.47 – 2.43 (m, 2H), 2.03 – 2.01 (m, 1H), 1.90 – 1.82 (m, 2H), 1.45 (s, 9H).

ESI - LCMS (m/z): calculated for $C_{13}H_{20}N_4NaO_4$ $(M+Na)^+$ = 319.13; found: 319.26.

Tert-butyl (S)-4-diazo-3-oxo-1-((S)-2-oxopyrrolidin-3-yl)butan-2-ylcarbamate (13, AP-8-260 (AP-7-273));

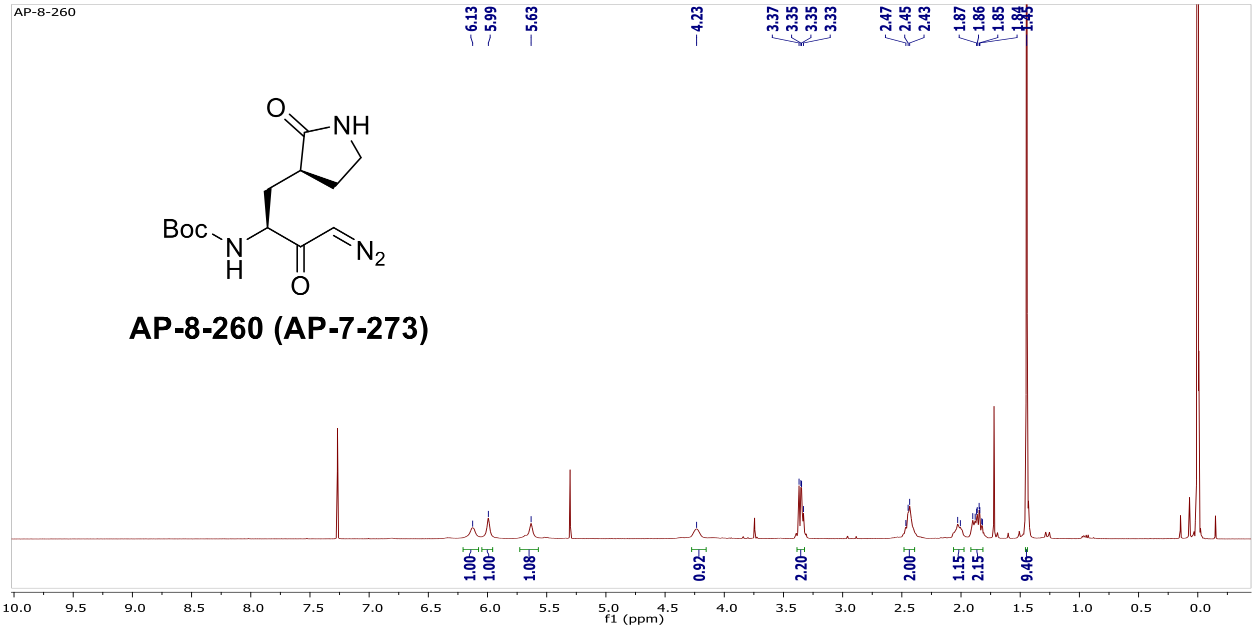

^{1}H NMR of Compound 13, AP-8-260 (AP-7-273)

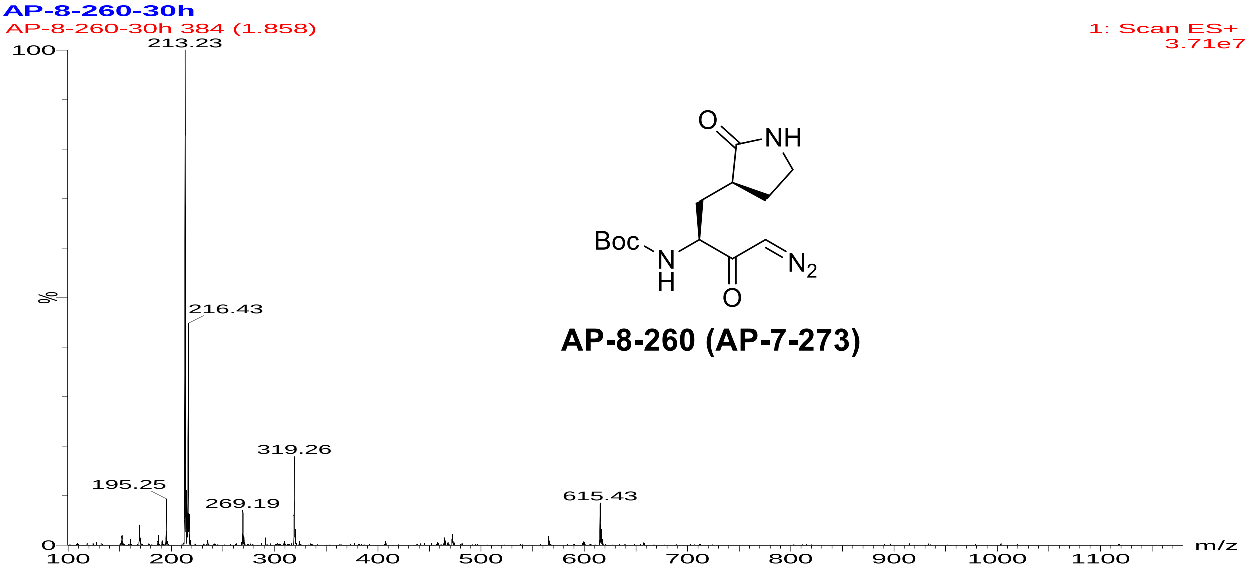

ESI-MS of Compound AP-8-260

Tert-butyl (S)-4-bromo-3-oxo-1-((S)-2-oxopyrrolidin-3-yl)butan-2-ylcarbamate (14, AP-7-276); To a stirred solution of tert-butyl ((1S)-3- chloro-2-oxo-1-[[(3S)-2-oxopyrrolidin-3-yl]methyl]propyl)carbamate (3.5 g, 11.81 mmol) in THF (100 mL) at -20 °C under nitrogen was treated with 48% hydrobromic acid (2.2 mL, 13.0 mmol) with effervescence observed. The reaction was stirred at 0 °C for 1 hour, washed once with water (50 mL), dried over Na_2SO_4, filtered, and concentrated to afford the title compound (3.69 g, 10.51 mmol, 89%) of the title

compound as a white solid. The product was confirmed by MS. The product was used for the next step without further purification.

ESI - LCMS (m/z): calculated for $C_{13}H_{21}BrN_2NaO_4$ $(M+Na)^+$ = 371.06; found: 371.23.

Tert-butyl (S)-4-bromo-3-oxo-1-((S)-2-oxopyrrolidin-3-yl)butan-2-ylcarbamate (14, AP-7-276 (AP-08-277));

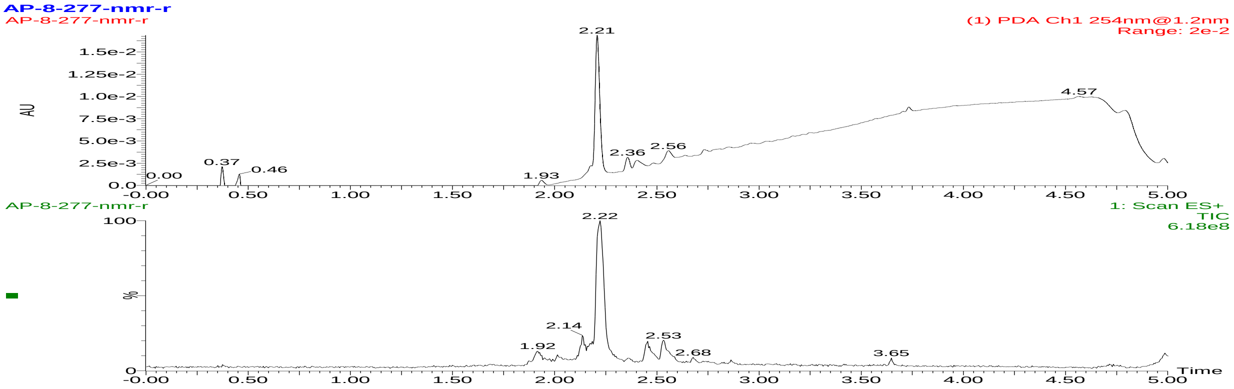

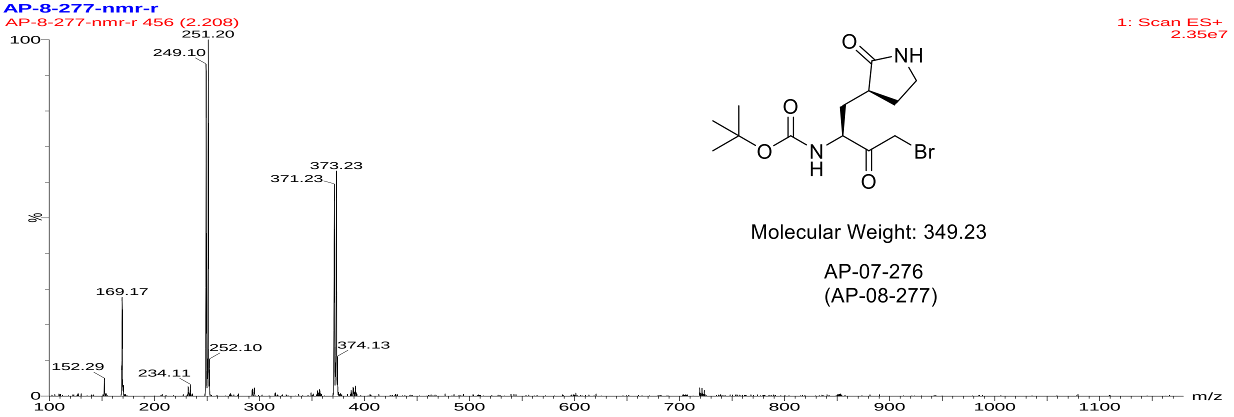

ESI-MS of Compound 14, AP-7-276 (AP-08-277)

(S)-3-((tert-butoxycarbonyl)amino)-2-oxo-4-((S)-2-oxopyrrolidin-3-yl)butyl 2,6-dichlorobenzoate (15, AP-9-21); To a stirred solution of tert-butyl (*S*)-4-bromo-3-oxo-1-((*S*)-2-oxopyrrolidin-3-yl)butan-2-ylcarbamate (3.69 g, 10.57 mmol) and 2,6-dichlorobenzoic acid (3.13 g, 16.38 mmol) in 60 mL anhydrous DMF at room temperature was added anhydrous cesium fluoride (4.81 g, 31.7 mmol) and reaction temperature was raised to 65 °C and stirred for 2 hours under a nitrogen atmosphere. Completion of the reaction was confirmed by LC-MS. Then the reaction was cooled to room temperature, diluted with water (100 mL) and with ethyl acetate (200 mL), washed once with brine (100 mL), dried over Na_2SO_4, filtered, and concentrated under reduced pressure. The crude product was purified by flash column chromatography to afford the title compound (3.98 g; 3.02 mmol, 82%). ^{1}H NMR (400 MHz, DMSO) δ 7.68 (s, 1H), 7.64 – 7.61 (m, 2H), 7.59 – 7.54 (m, 2H), 5.22 (d, *J* = 2.6 Hz, 2H), 4.24 – 4.17 (m, 1H), 3.17 – 3.13 (m, 2H), 2.30 – 2.12 (m, 2H), 1.98 – 1.90 (m, 1H), 1.73 – 1.64 (m, 2H), 1.41 (s, 9H). ^{13}C NMR (100 MHz, DMSO) δ 202.3, 178.7, 163.8, 156.2, 133.1, 132.5, 131.3, 129.0, 68.2, 56.1, 38.0, 31.4, 28.6, 27.6.

ESI-HRMS (m/z): calculated for $C_{20}H_{25}Cl_2N_2O_6$ $(M+H)^+$ = 459.1090 found: 459.1077

(S)-3-((tert-butoxycarbonyl)amino)-2-oxo-4-((S)-2-oxopyrrolidin-3-yl)butyl 2,6-dichlorobenzoate (15, AP-9-21);

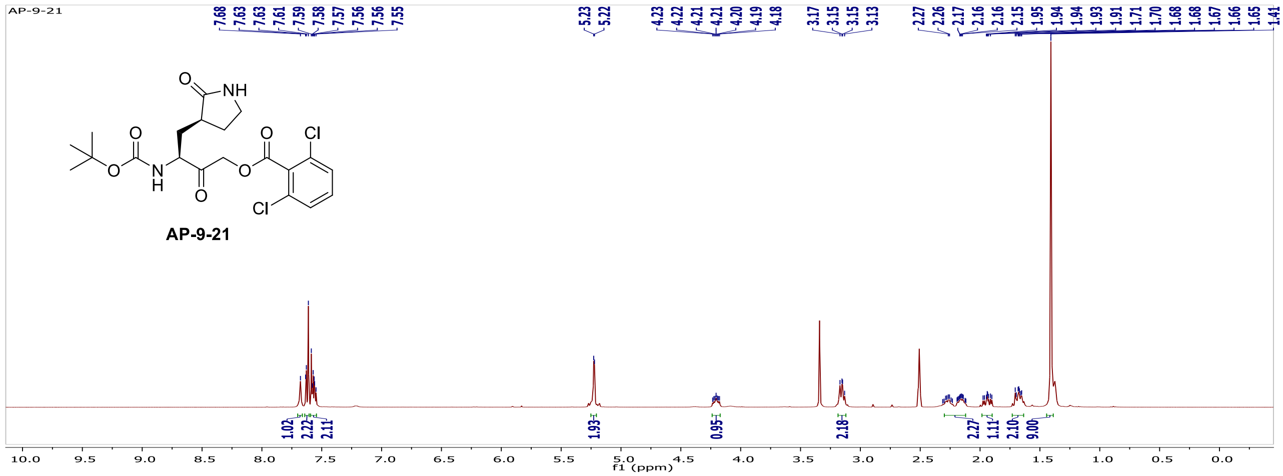

^{1}H NMR of Compound 15, AP-9-21

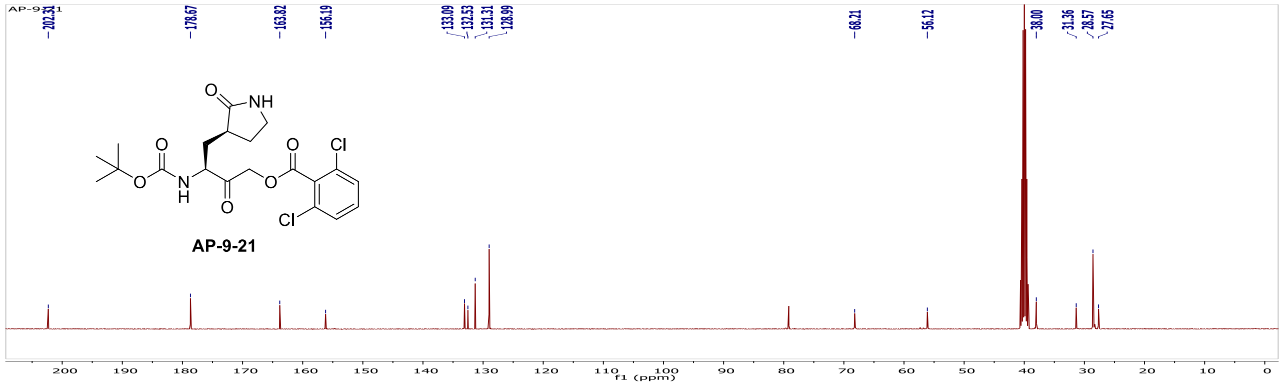

^{13}C NMR of Compound 15, AP-9-21

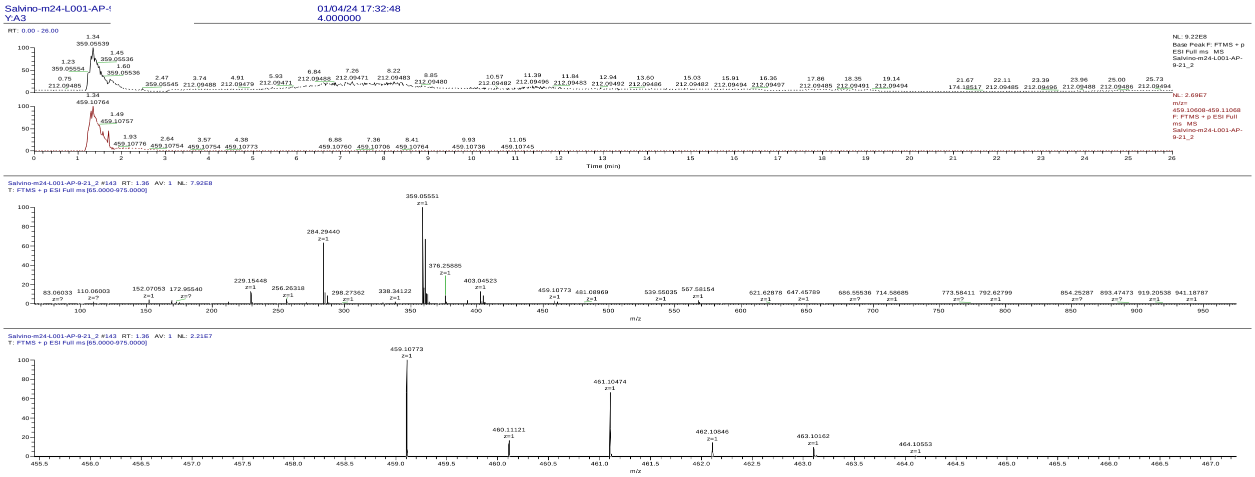

HRMS of Compound 15, AP-9-21

(*S*)-3-Amino-2-oxo-4-((*S*)-2-oxopyrrolidin-3-yl) butyl 2,6-dichlorobenzoate 2,2,2-trifluoroacetate (7, AP-7-297B (AP-9-022); To a stirred solution of (*S*)-ethyl 2-(3-(tert-butoxycarbonylamino)-4-oxo-3,4-dihydrobenzo[*b*][1,4] oxazepin-5(2*H*)-yl)acetate (500 mg, 1.09 mmol) in 16 mL of CH_2Cl_2 at 0 °C was added 4 mL of TFA. The reaction mixture was then warmed to room temperature and stirred for 3 hours. Completion of the reaction was confirmed by LC−MS. Volatiles were evaporated under reduced pressure and the crude product co-distilled with dry toluene, then dried under high vacuum to afford the title compound (515 mg; 1.09 mmol, 100%). ^{1}H NMR (400 MHz, DMSO) δ 8.68 (s, 1H), 8.03 (s, 1H), 7.66 – 7.60 (m, 2H), 5.57 – 5.53 (m, 2H), 4.38 – 4.36 (m, 1H), 3.37 (s, 2H), 3.23 – 3.19 (m, 2H), 2.70 – 2.59 (m, 1H), 2.39 – 2.28 (m, 1H), 2.12 – 2.06 (m, 1H), 1.99 – 1.91 (m, 1H), 1.81 – 1.71 (m, 1H). ^{13}C NMR (100 MHz, DMSO) δ 199.4, 178.6, 163.8, 133.3, 132.3, 131.3, 129.1, 68.2, 66.8, 55.1, 38.8, 31.3, 28.0.
ESI-HRMS (m/z): calculated for $C_{15}H_{17}Cl_2N_2O_4$ $(M+H)^+$ = 359.0565 found: 359.0555.

(*S*)-3-Amino-2-oxo-4-((*S*)-2-oxopyrrolidin-3-yl) butyl 2,6-dichlorobenzoate 2,2,2-trifluoroacetate (7, AP-7-297B (AP-9-022);

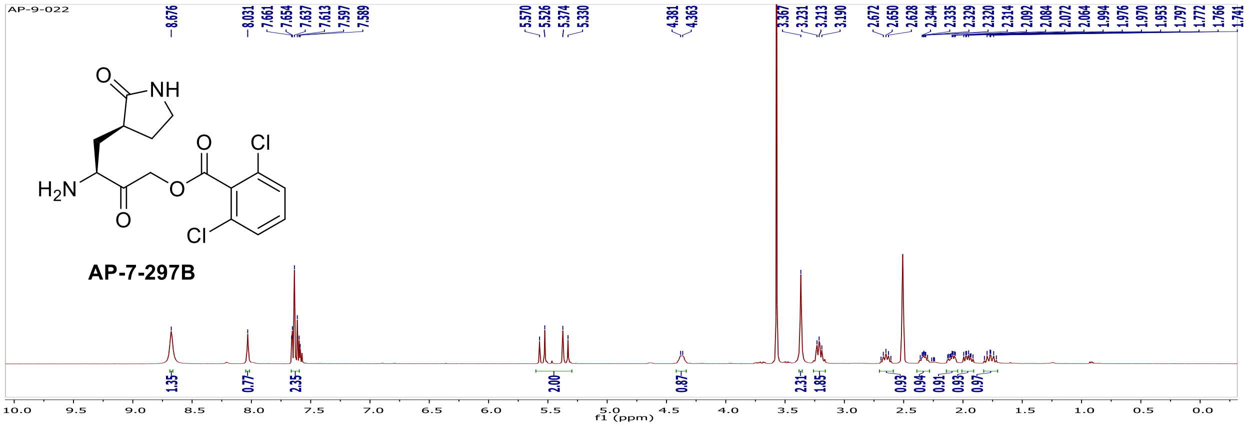

^{1}H NMR of Compound 7, AP-7-297B (AP-9-022)

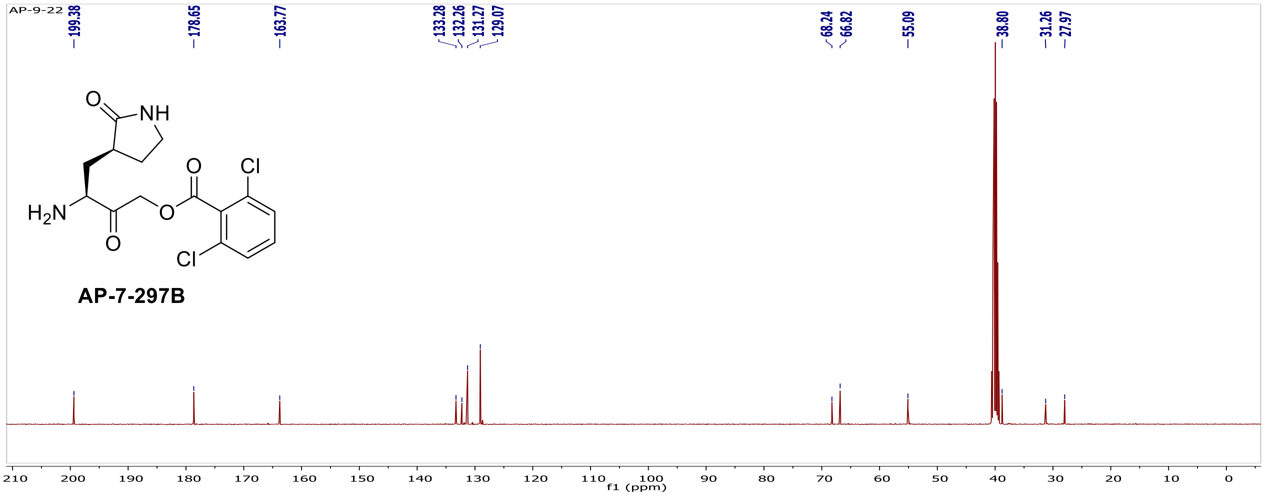

^{13}C NMR of Compound 7, AP-7-297B (AP-9-022)

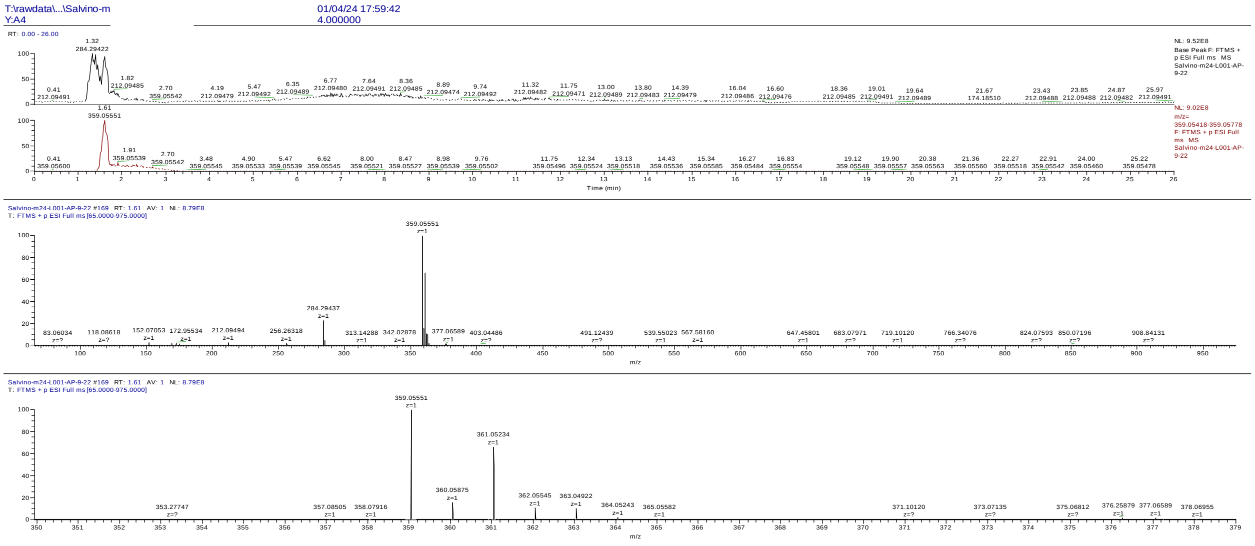

HRMS of Compound 7, AP-7-297B (AP-9-022)

Figure 6. Synthesis of Pfizer compound **18**.

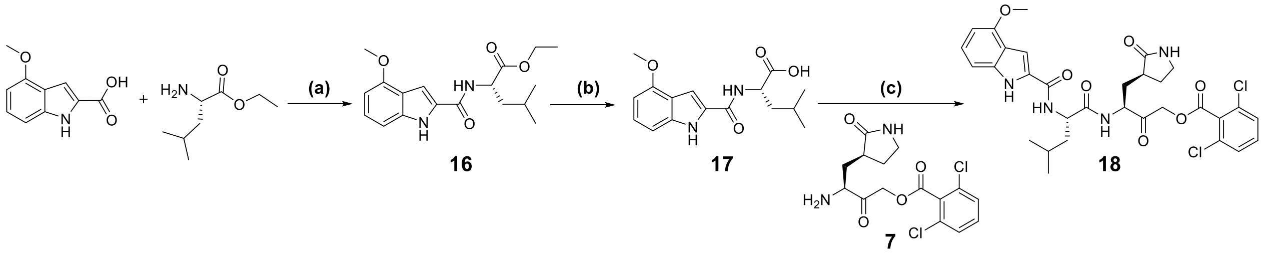

Ethyl (4-methoxy-1*H*-indole-2-carbonyl)-L-leucinate (16, AP-7-296); To a stirred solution of 4-methoxy-1*H*-indole-2-carboxylic acid (464 mg; 2.4 mmol) and (*S*)-ethyl 2-amino-4-methylpentanoate (382 mg; 2.4 mmol) in 20 mL dry CH_2Cl_2 at 0 °C was added diisopropyl ethyl amine (1.0 mL, 5.52 mmol) and T3P 50% solution in DMF by weight (1.98 g; 3.12 mmol) dropwise simultaneously. The reaction mixture was stirred for an additional 1 hour at 0 °C, and completion of the reaction was confirmed by LC−MS. The reaction mixture was quenched with cold water and the product was extracted with CH_2Cl_2 and dried over anhydrous Na_2SO_4. The solvent was removed under reduced pressure to provide the crude reaction product, which was purified by flash column chromatography to afford the title compound as a white solid (638 mg, 1.92 mmol, 80%). ^{1}H NMR (400 MHz, DMSO) δ 11.59 (s, 1H), 8.62 (d, *J* = 7.7 Hz, 1H), 7.36 (d, *J* = 1.7 Hz, 1H), 7.11 (t, *J* = 8.0 Hz, 1H), 7.01 (d, *J* = 8.2 Hz, 1H), 6.52 (d, *J* = 7.6 Hz, 1H), 4.52 – 4.46 (m, 1H), 4.12 (q, *J* = 7.1 Hz, 2H), 3.89 (s, 3H), 1.82 – 1.69 (m, 2H), 1.61 – 1.55 (m, 1H), 1.20 (t, *J* = 7.1 Hz, 3H), 0.95 (d, *J* = 6.4 Hz, 3H), 0.90 (d, *J* = 6.4 Hz, 3H). ^{13}C NMR (100 MHz, DMSO) δ 173.1, 161.7, 154.2, 138.3, 130.1, 125.0, 118.5, 105.8, 101.6, 99.7, 60.9, 55.5, 51.1, 24.9, 23.3, 21.6, 14.6.

ESI-HRMS (m/z): calculated for $C_{18}H_{25}N_2O_4$ $(M+H)^+$ = 333.1814 found: 333.1805

Ethyl (4-methoxy-1*H*-indole-2-carbonyl)-L-leucinate (16, AP-7-296);

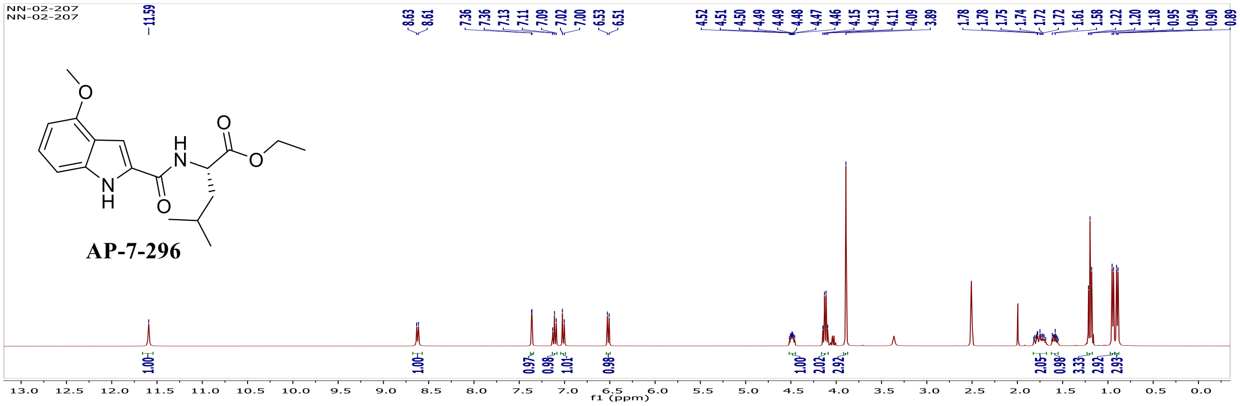

^{1}H NMR of Compound 16, AP-7-296

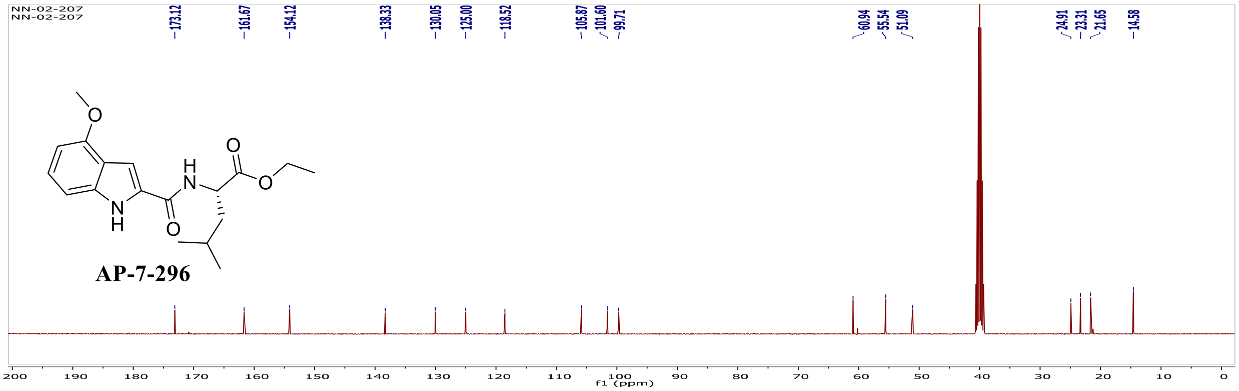

^{13}C NMR of Compound 16, AP-7-296

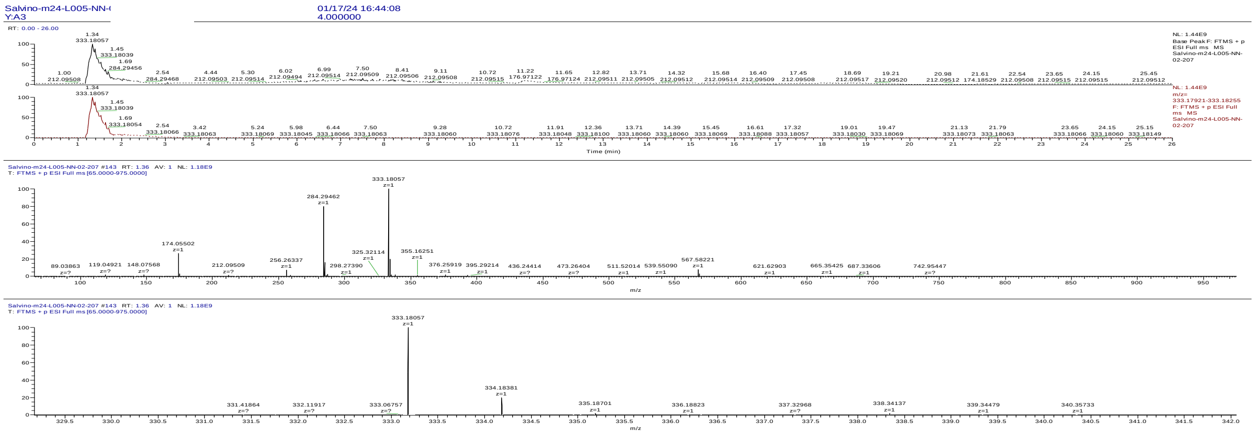

HRMS of Compound 16, AP-7-296

(*S*)-2-(4-Methoxy-1*H*-indole-2-carboxamido)-4-methylpentanoic acid (17, AP-7-296B); To a stirred solution of (*S*)-ethyl 2-(4-methoxy-1*H*-indole-2-carboxamido)-4-methylpentanoate (600 mg; 1.8 mmol) in 20 mL of MeOH, H_2O and THF (4:2:1) at room temperature was added LiOH·H_2O (238mg; 5.4 mmol). The reaction mixture was stirred for 5 hours. Completion of the reaction was confirmed by LCMS. Volatiles were evaporated by reduced pressure, and the crude product was acidified with 1 N HCl at 0 °C to obtain a white precipitate which was filtered and

dried under a high vacuum to afford the title compound (493 mg; 16.2 mmol, 90 %) as a white solid. ^{1}H NMR (400 MHz, DMSO) δ 12.54 (s, 1H), 11.52 (s, 1H), 8.46 (d, J = 8.0 Hz, 1H), 7.30 (d, J = 1.7 Hz, 1H), 7.05 (t, J = 7.9 Hz, 1H), 6.96 (d, J = 8.2 Hz, 1H), 6.46 (d, J = 7.6 Hz, 1H), 4.46 – 4.33 (m, 1H), 3.83 (s, 3H), 1.75 – 1.65 (m, 2H), 1.60 – 1.49 (m, 1H), 0.88 (d, J = 6.4 Hz, 3H), 0.84 (d, J = 6.4 Hz, 3H). ^{13}C NMR (100 MHz, DMSO) δ 174.7, 161.6, 154.1, 138.3, 130.3, 124.9, 118.5, 105.9, 101.4, 99.7, 55.5, 50.9, 25.0, 23.4, 21.6.
ESI- HRMS (m/z): calculated for $C_{16}H_{21}N_2O_4$ (M+H)$^+$ = 305.1501found: 305.1490

(S)-2-(4-Methoxy-1H-indole-2-carboxamido)-4-methylpentanoic acid (17, AP-7-296B);

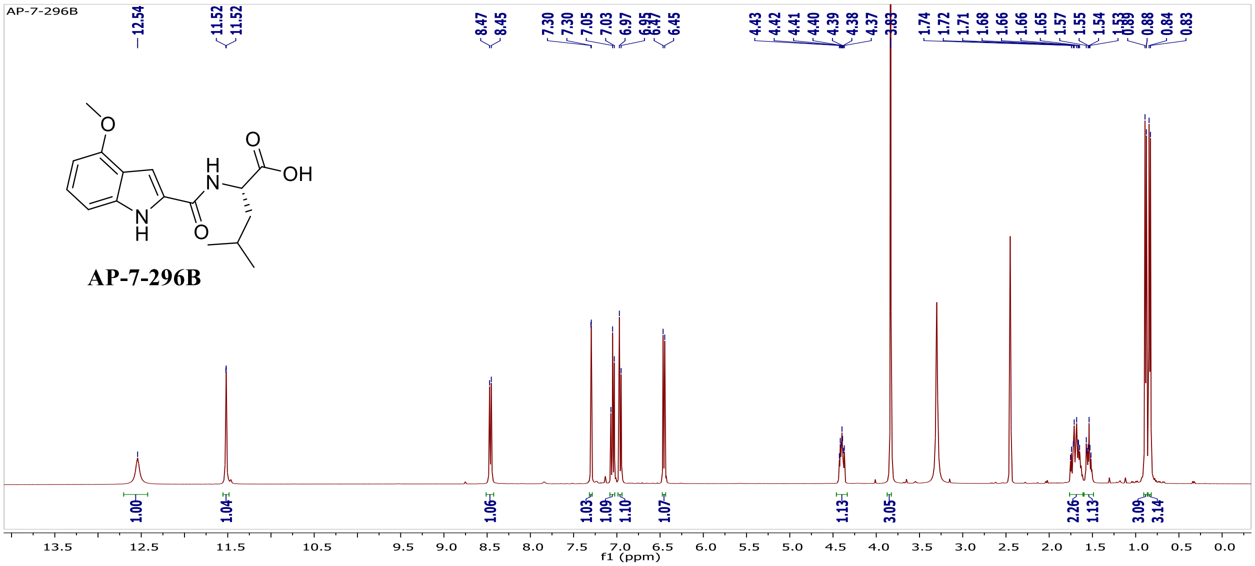

^{1}H NMR of Compound 17, AP-7-296B

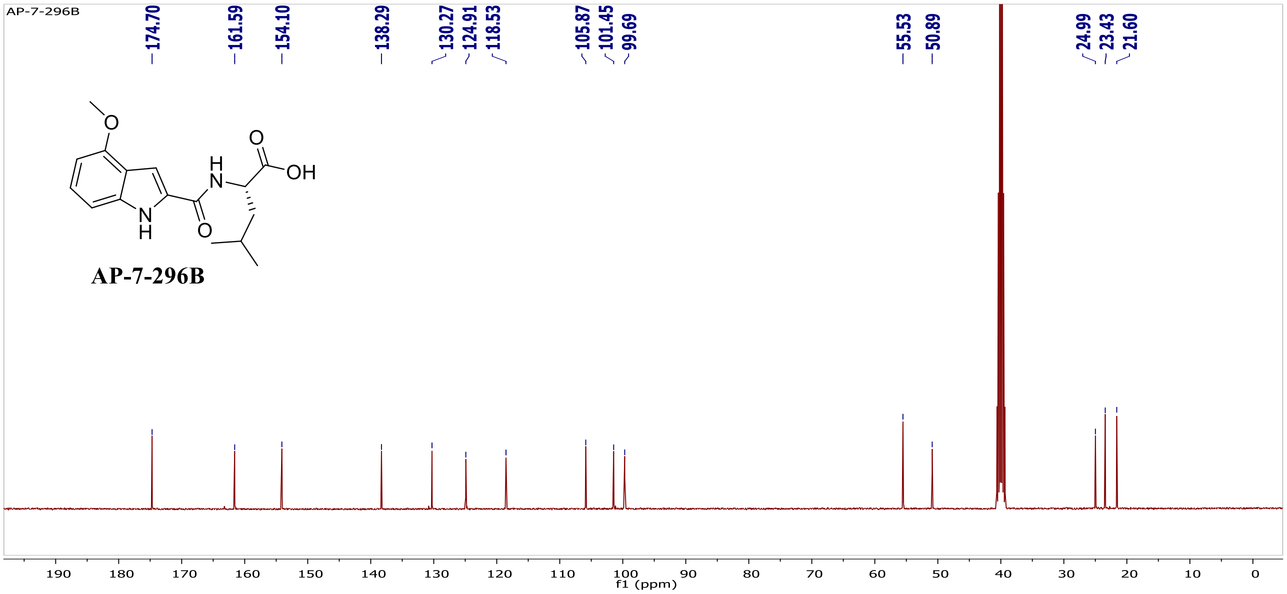

^{13}C NMR of Compound 17, AP-7-296B

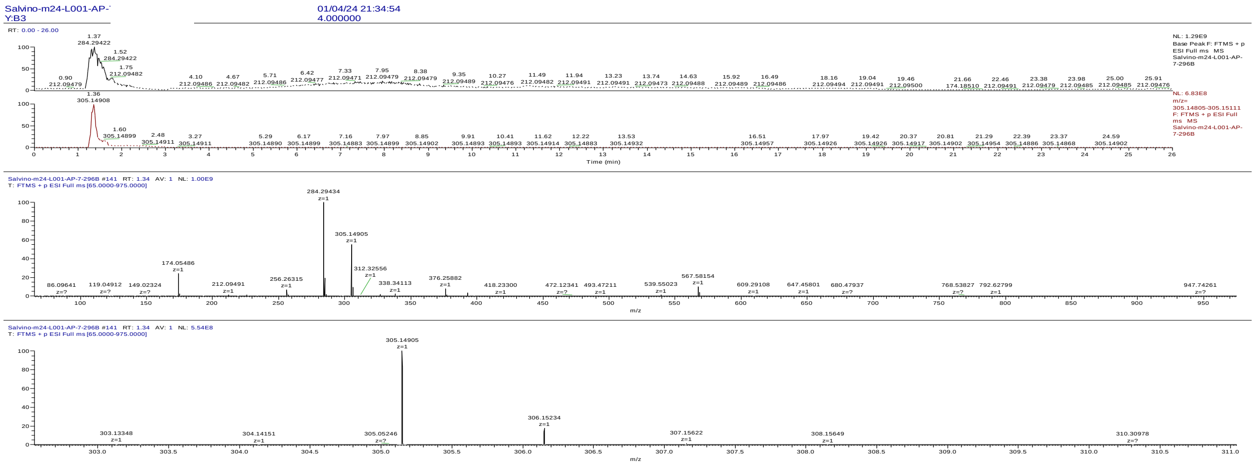

HRMS of Compound 17, AP-7-296B

(*S*)-3-((*S*)-2-(4-Methoxy-1*H*-indole-2-carboxamido)-4-methylpentanamido)-2-oxo-4-((*S*)-2-oxopyrrolidin-3-yl)butyl 2,6-dichlorobenzoate (18, AP-8-001); To a stirred solution of (*S*)-2-(4-methoxy-1*H*-indole-2-carboxamido)-4-methylpentanoic acid (300 mg; 0.98 mmol) and (*S*)-3-amino-2-oxo-4-((*S*)-2-oxopyrrolidin-3-yl)butyl 2,6-dichlorobenzoate hydrochloride (387 mg; 0.98 mmol) in 20 mL dry CH_2Cl_2 at 0 °C was added diisopropyl ethyl amine (0.7 mL, 0.7 mmol) and T3P 50% solution in DMF by weight (810 mg; 1.27 mmol) dropwise simultaneously. The reaction mixture was stirred for an additional 1 hour at 0 °C, and completion of the reaction was confirmed by LC−MS. The reaction mixture was quenched with cold water and the product was extracted with CH_2Cl_2 and dried over anhydrous Na_2SO_4. The solvent was removed under reduced pressure to provide the crude reaction product, which was purified by flash column chromatography to afford the title compound as a white solid (506 mg, 0.78 mmol, 80%). ^{1}H NMR (400 MHz, DMSO) δ 11.59 – 11.58 (m, 1H), 8.70 – 8.63 (m, 1H), 8.49 – 8.46 (m, 1H), 7.67 (d, J = 5.0 Hz, 1H), 7.63 – 7.60 (m, 2H), 7.58 – 7.54 (m, 1H), 7.38 (s, 1H), 7.10 (t, J = 7.9 Hz, 1H), 7.02 – 6.99 (m, 1H), 6.51 (d, J = 7.7 Hz, 1H), 5.31 – 5.16 (m, 2H), 4.54 – 4.50 (m, 2H), 3.89 (s, 3H), 3.17 – 3.09 (m, 2H), 2.35 – 2.20 (m, 2H), 2.14 – 2.00 (m, 2H), 1.79 – 1.66 (m, 4H), 0.96 – 0.94 (m, 3H), 0.91 (d, J = 6.3 Hz, 3H). ^{13}C NMR (100 MHz, DMSO) δ 201.5, 178.7, 173.5, 163.8, 161.7, 154.1, 138.3, 133.1, 132.5, 131.3, 131.3, 130.3, 130.2, 129.0, 124.9, 118.5, 105.9, 101.7, 99.7, 68.3, 55.5, 54.4, 52.1, 37.7, 27.6, 25.0, 24.9, 23.5, 21.8, 14.4.

ESI- HRMS (m/z): calculated for $C_{31}H_{35}Cl_2N_4O_7$ $(M+H)^+$ = 645.1883 found: 645.1871. ESI-LCMS (m/z): 645.42 $(M+H)^+$.

(S)-3-((S)-2-(4-Methoxy-1H-indole-2-carboxamido)-4-methylpentanamido)-2-oxo-4-((S)-2-oxopyrrolidin-3-yl)butyl 2,6-dichlorobenzoate (18, AP-8-001);

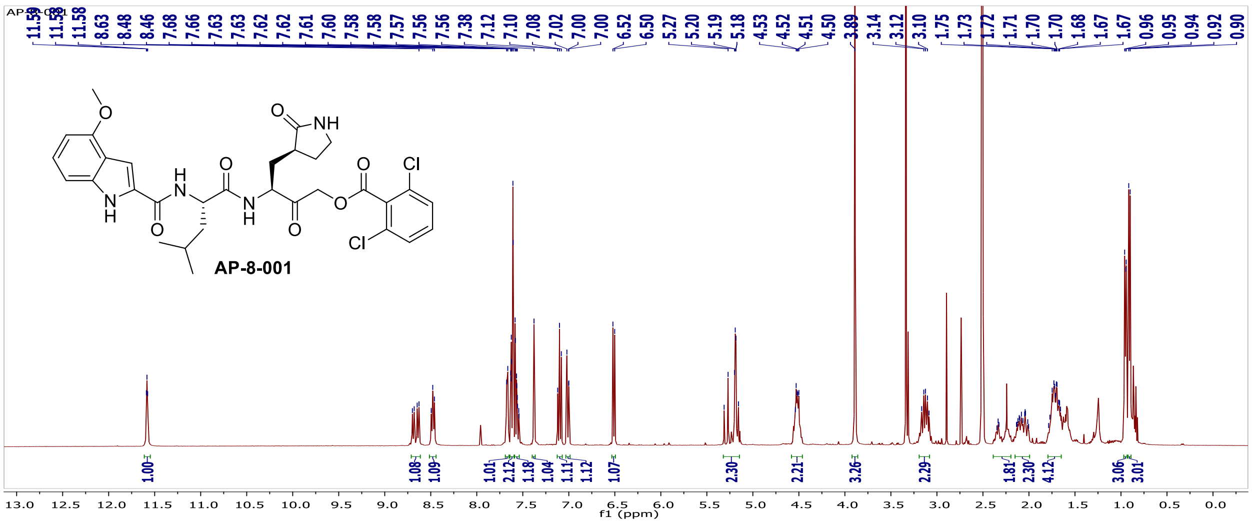

^{1}H NMR of Compound 18, AP-8-001

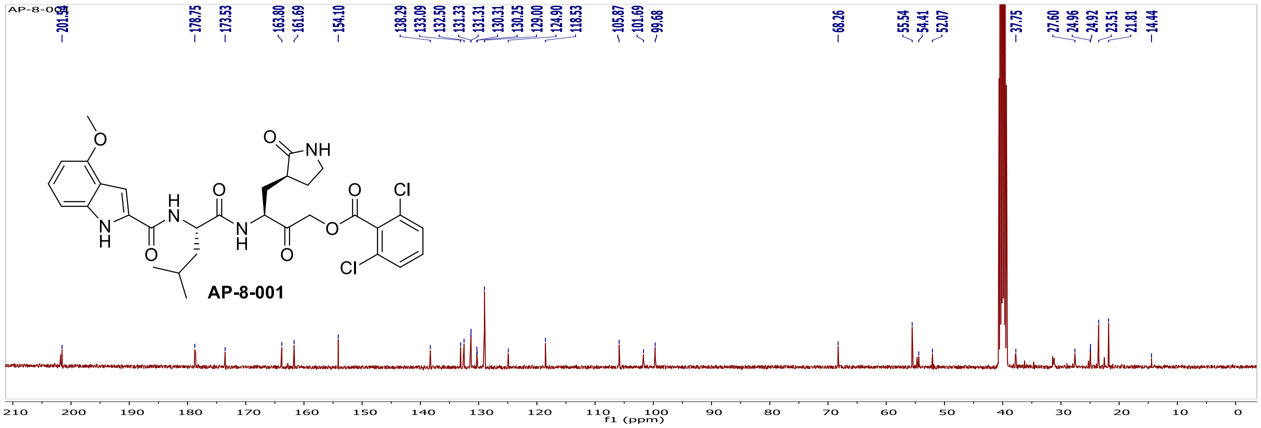

^{13}C NMR of Compound 18, AP-8-001

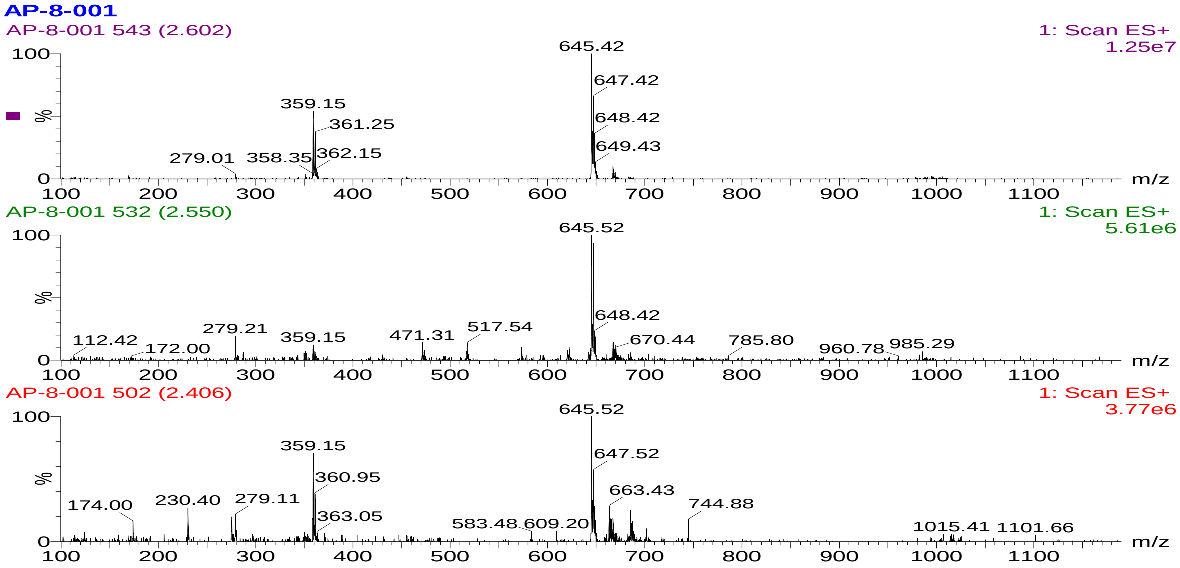

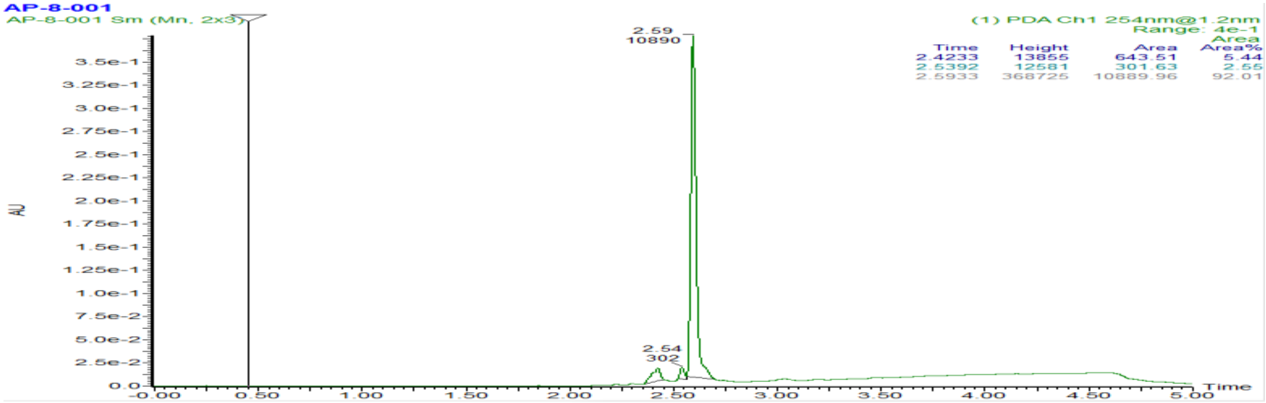

LCMS of Compound 18, AP-8-001

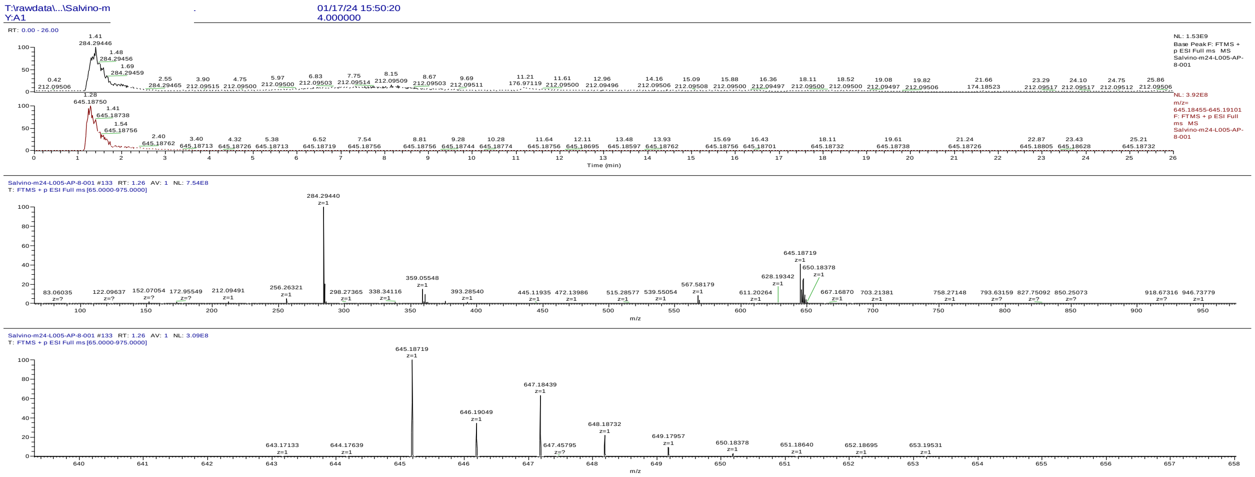

HRMS of Compound 18, AP-8-001

(*S*)-3-(2-((*S*)-3-Benzamido-4-oxo-3,4-dihydrobenzo[*b*][1,4]oxazepin-5(2*H*)-yl)acetamido)-2-oxo-4-((*S*)-2-oxopyrrolidin-3-yl)butyl 2,6-dichlorobenzoate (19, AP-8-011); To a stirred solution of (*S*)-3-(2-((*S*)-3-amino-4-oxo-3,4-dihydrobenzo[*b*][1,4]oxazepin-5(2*H*)-yl)acetamido)-2-oxo-4-((*S*)-2-oxopyrrolidin-3-yl)butyl 2,6-dichlorobenzoate hydrochloride (30 mg; 0.05 mmol) in 5 mL of dry CH_2Cl_2 at 0 °C was added triethylamine (25 mg; 0.25 mmol) and benzoyl chloride (14 mg, 0.1 mmol) dropwise simultaneously. The reaction mixture was allowed to stir for 12 hours at 0 °C. Completion of the reaction was confirmed by LC−MS. The reaction mixture was quenched with cold water and the product was extracted with CH_2Cl_2 and dried over anhydrous Na_2SO_4. The solvent was evaporated under reduced pressure and the crude product was purified by reverse phase HPLC to afford the title compound (15 mg, 0.02 mmol, 45%) as a white colored solid. ^{1}H NMR (400 MHz, DMSO) δ 8.65 – 8.57 (m, 2H), 7.77 – 7.75 (m, 2H), 7.59 (s, 1H), 7.54 – 7.45 (m, 4H), 7.41 – 7.36 (m, 3H), 7.27 – 7.20 (m, 2H), 7.19 – 7.16 (m, 1H), 5.15 – 5.11 (m, 1H), 5.01 – 4.92 (m, 2H), 4.55 – 4.44 (m, 3H), 4.39 – 4.33 (m, 2H), 3.11 – 3.01 (m, 2H), 2.26 – 2.17 (m, 1H), 2.08 – 2.01 (m, 1H), 1.97 – 1.91 (m, 1H), 1.65 – 1.56 (m, 2H). ^{13}C NMR (100 MHz, DMSO)

δ 201.7, 178.6, 169.9, 168.8, 166.6, 163.7, 150.1, 136.5, 133.9, 133.1, 132.5, 132.1, 131.3, 129.0, 128.7, 127.9, 127.8, 126.1, 124.0, 123.0, 76.2, 68.2, 54.7, 51.3, 49.6, 37.7, 31.4, 27.6.
ESI-HRMS (m/z): calculated for $C_{33}H_{31}Cl_2N_4O_8$ $(M+H)^+$ = 681.1519 found: 681.1500. ESI-LCMS (m/z): 681.34 $(M+H)^+$.

(S)-3-(2-((S)-3-Benzamido-4-oxo-3,4-dihydrobenzo[b][1,4]oxazepin-5(2H)-yl)acetamido)-2-oxo-4-((S)-2-oxopyrrolidin-3-yl)butyl 2,6-dichlorobenzoate (19, AP-8-011);

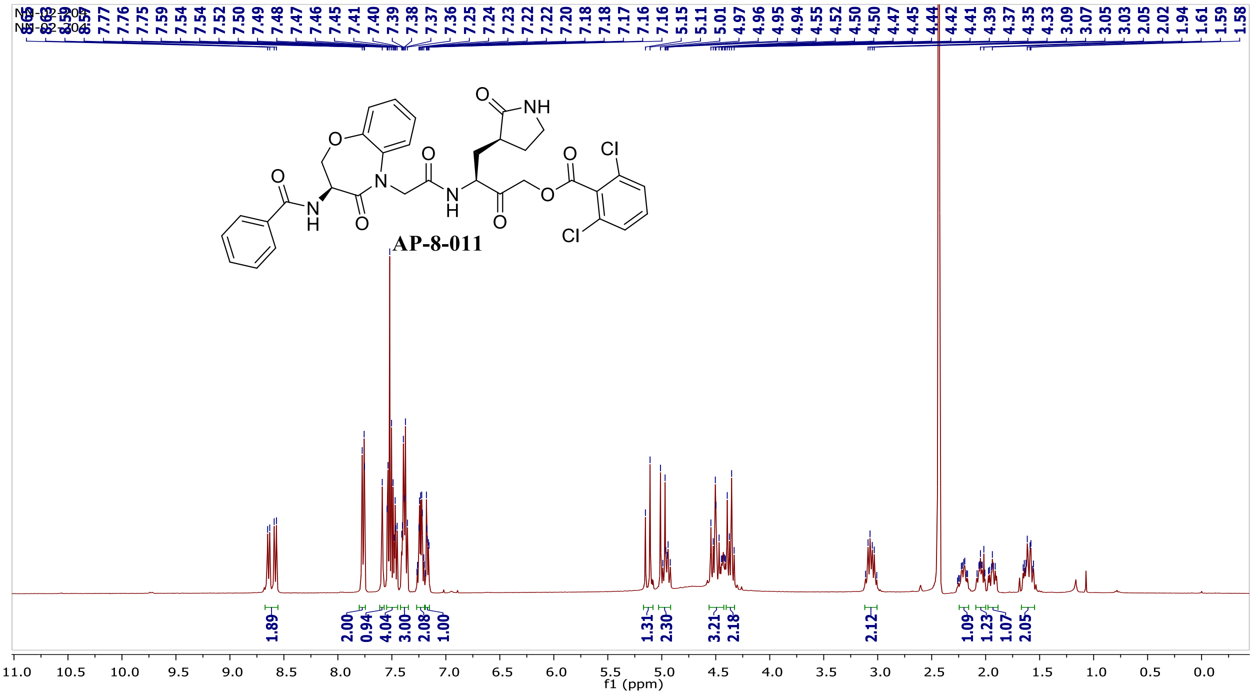

^{1}H NMR of Compound 19, AP-8-011

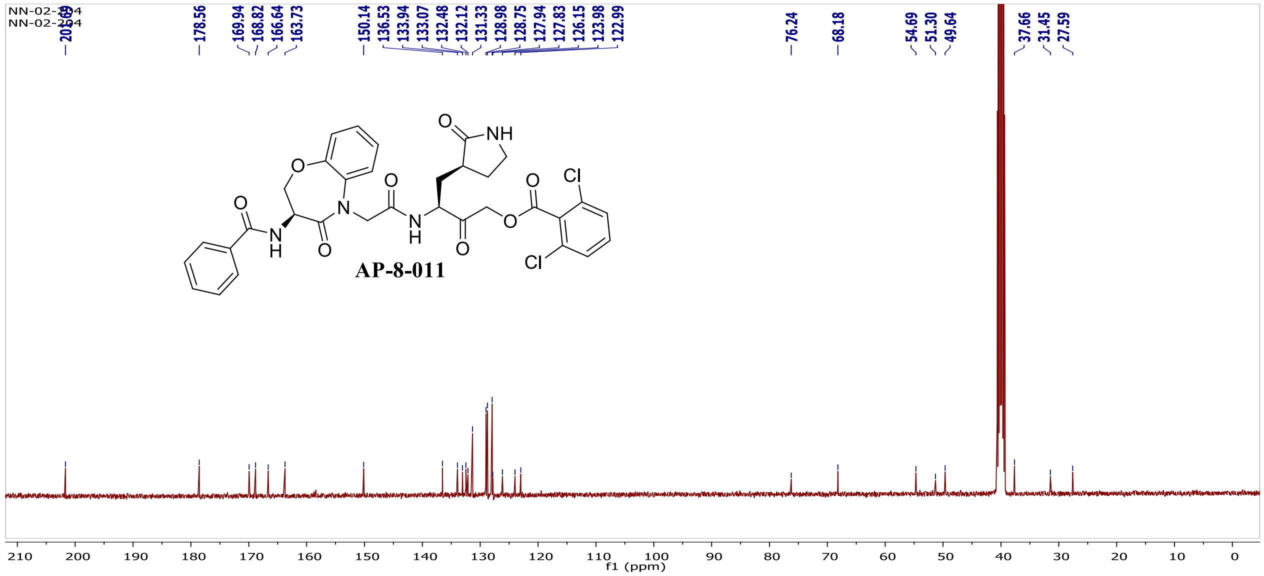

^{13}C NMR of Compound 19, AP-8-011

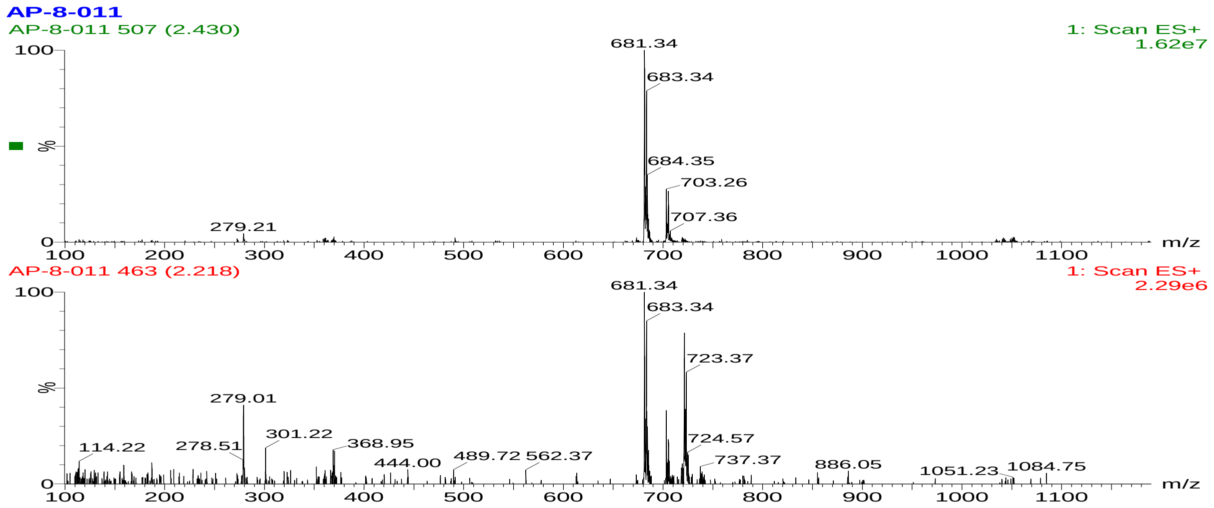

MS of Compound 19, AP-8-011

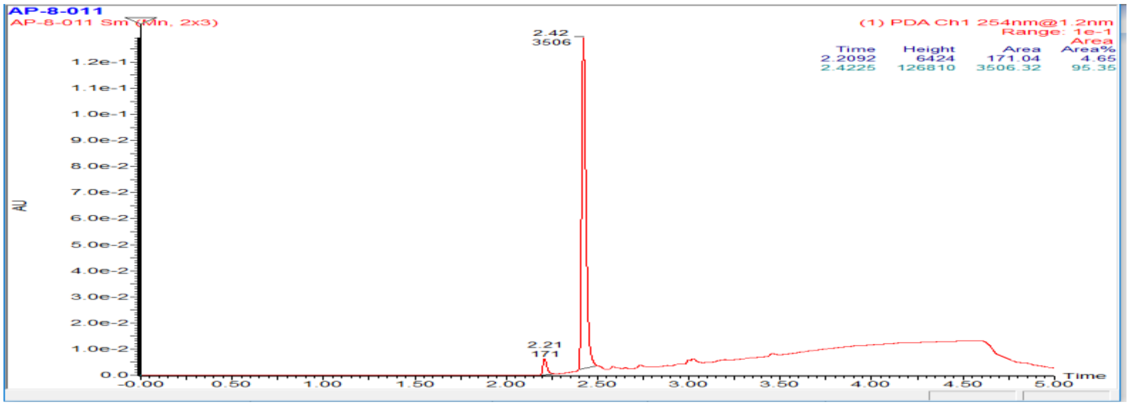

LCMS of Compound 19, AP-8-011

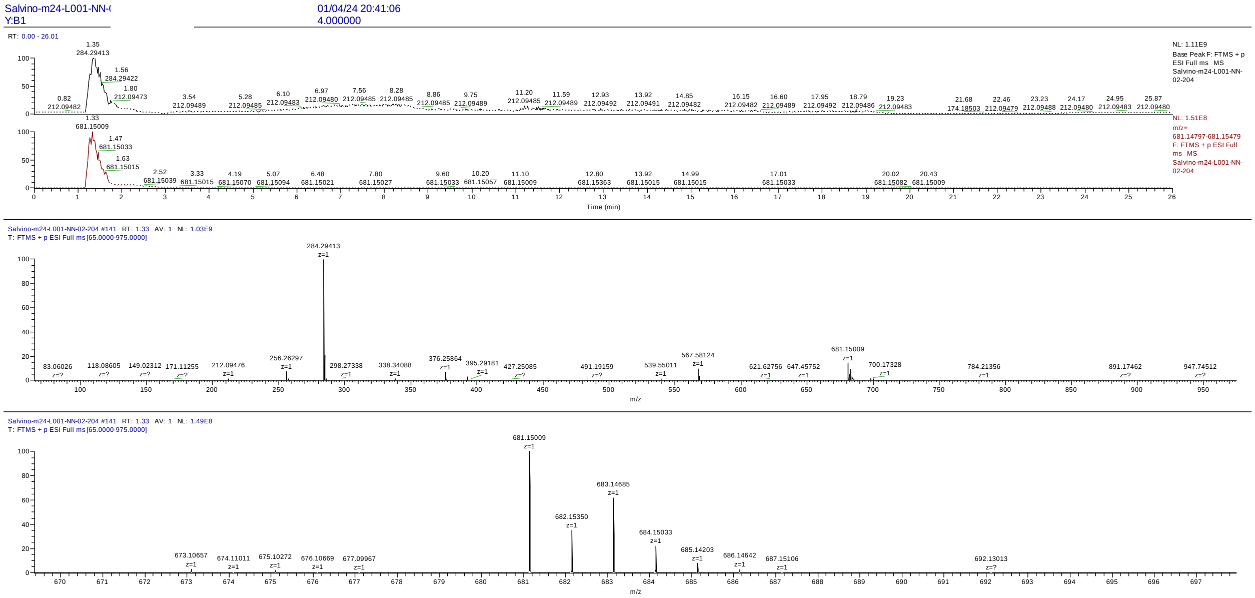

HRMS of Compound 19, AP-8-011

(*S*)-3-(2-((*S*)-3-(Isoquinoline-3-carboxamido)-4-oxo-3,4-dihydrobenzo[*b*][1,4]oxazepin-5(2*H*)-yl)acetamido)-2-oxo-4-((*S*)-2-oxopyrrolidin-3-yl)butyl 2,6-dichlorobenzoate (20, AP-8-012); To a stirred solution of (*S*)-2-(3-(tert-butoxycarbonylamino)-4-oxo-3,4-dihydrobenzo [*b*][1,4]oxazepin-5(2*H*)-yl)acetic acid (20 mg; 0.03 mmol) and Isoquinoline-3-carboxylic acid (6 mg, 0.03 mmol) in 20 mL dry CH_2Cl_2 at 0 °C was added diisopropyl ethyl amine (21 mg, 0.04 mmol) and T3P 50% solution in CH_2Cl_2 by weight (17 mg; 1.65 mmol) dropwise simultaneously. The reaction mixture was stirred for an additional 1 hour at 0 °C, and completion of the reaction was confirmed by LC−MS. The reaction mixture was quenched with cold water and the product was extracted with CH_2Cl_2 and dried over anhydrous Na_2SO_4. The solvent was removed under reduced pressure to provide the crude reaction product, which was purified by flash column chromatography to afford the title compound as a white solid (10 mg, 42%). ^{1}H NMR (400 MHz, DMSO) δ 9.40 (s, 1H), 9.03 (d, J = 7.4 Hz, 1H), 8.82 (d, J = 7.2 Hz, 1H), 8.53 (s, 1H), 8.27 – 8.17 (m, 2H), 7.89 – 7.81 (m, 2H), 7.64 – 7.46 (m, 5H), 7.33 – 7.31 (m, 3H), 5.26 – 5.22 (m, 1H), 5.14 – 5.03 (m, 2H), 4.72 – 4.68 (m, 1H), 4.59 – 4.57 (m, 2H), 4.42 – 4.38 (m, 1H), 3.14 – 3.11 (m, 2H), 2.32 – 2.30 (m, 1H), 2.05 – 2.00 (m, 1H), 1.69 – 1.64 (m, 2H), 1.28 – 1.25 (m, 2H). ^{13}C NMR (100 MHz, DMSO) δ 201.7, 178.6, 169.6, 168.8, 164.1, 163.7, 152.2, 150.1, 143.0, 136.3, 135.7, 133.0, 132.4, 131.9, 131.3, 129.9, 128.9, 128.5, 128.4, 128.1, 126.2, 124.1, 123.0, 120.6, 76.7, 68.2, 54.7, 51.7, 49.6, 37.7, 31.3, 27.6, 17.2.

ESI-HRMS (m/z): calculated for $C_{36}H_{32}Cl_2N_5O_8$ $(M+H)^+$ = 732.1628 found: 732.1610. ESI-LCMS (m/z): 732.37 $(M+H)^+$.

(S)-3-(2-((S)-3-(isoquinoline-3-carboxamido)-4-oxo-3,4-dihydrobenzo[b][1,4]oxazepin-5(2H)-yl)acetamido)-2-oxo-4-((S)-2-oxopyrrolidin-3-yl)butyl 2,6-dichlorobenzoate (20, AP-8-012);

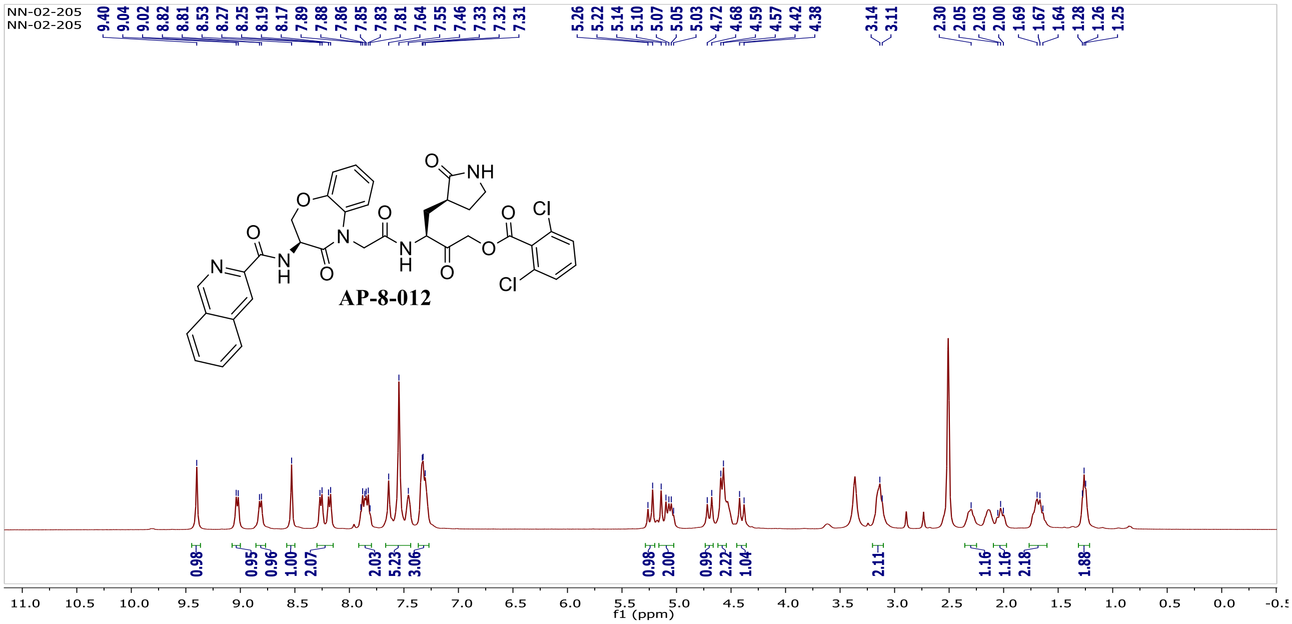

^{1}H NMR of Compound 20, AP-8-012

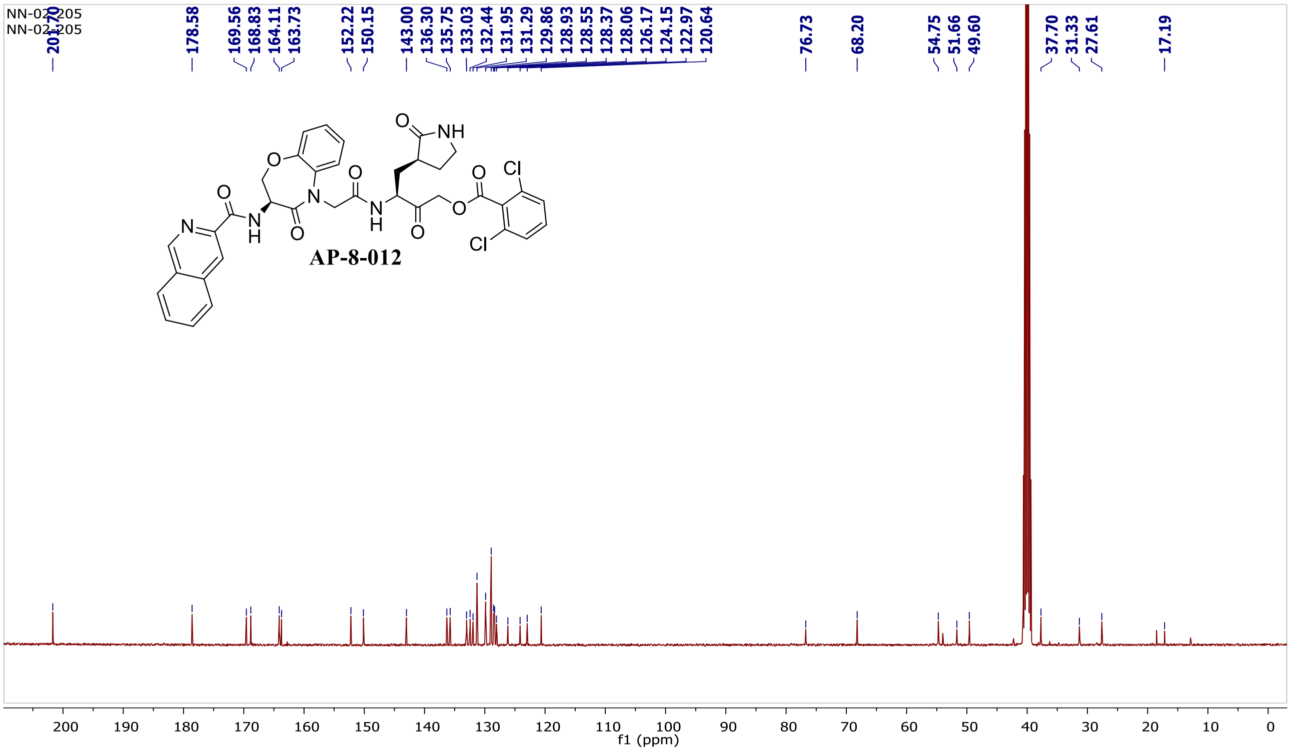

^{13}C NMR of Compound 20, AP-8-012

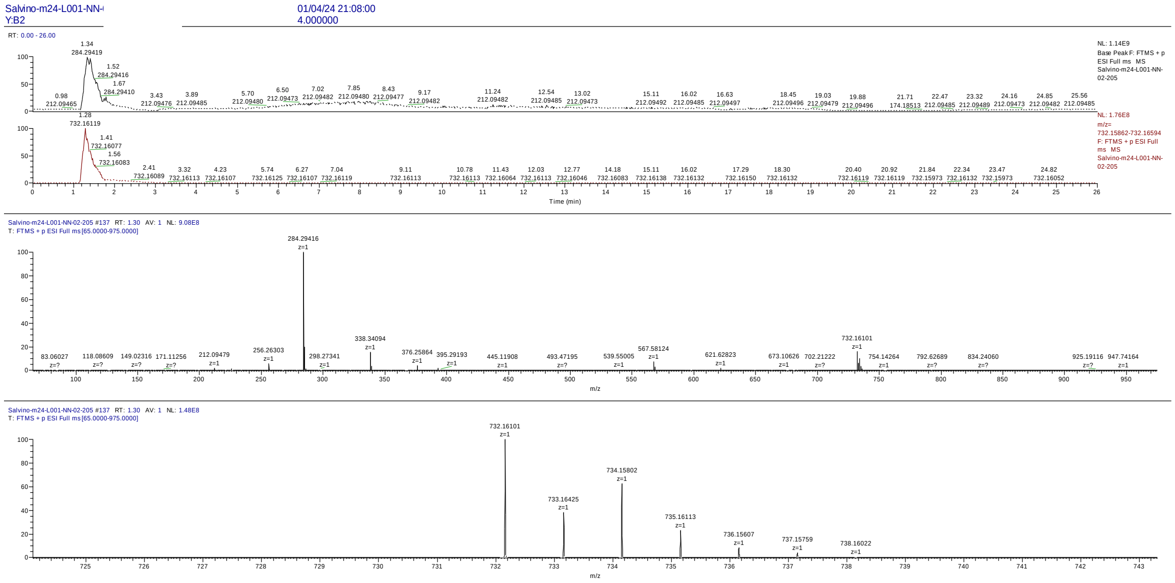

HRMS of Compound 20, AP-8-012

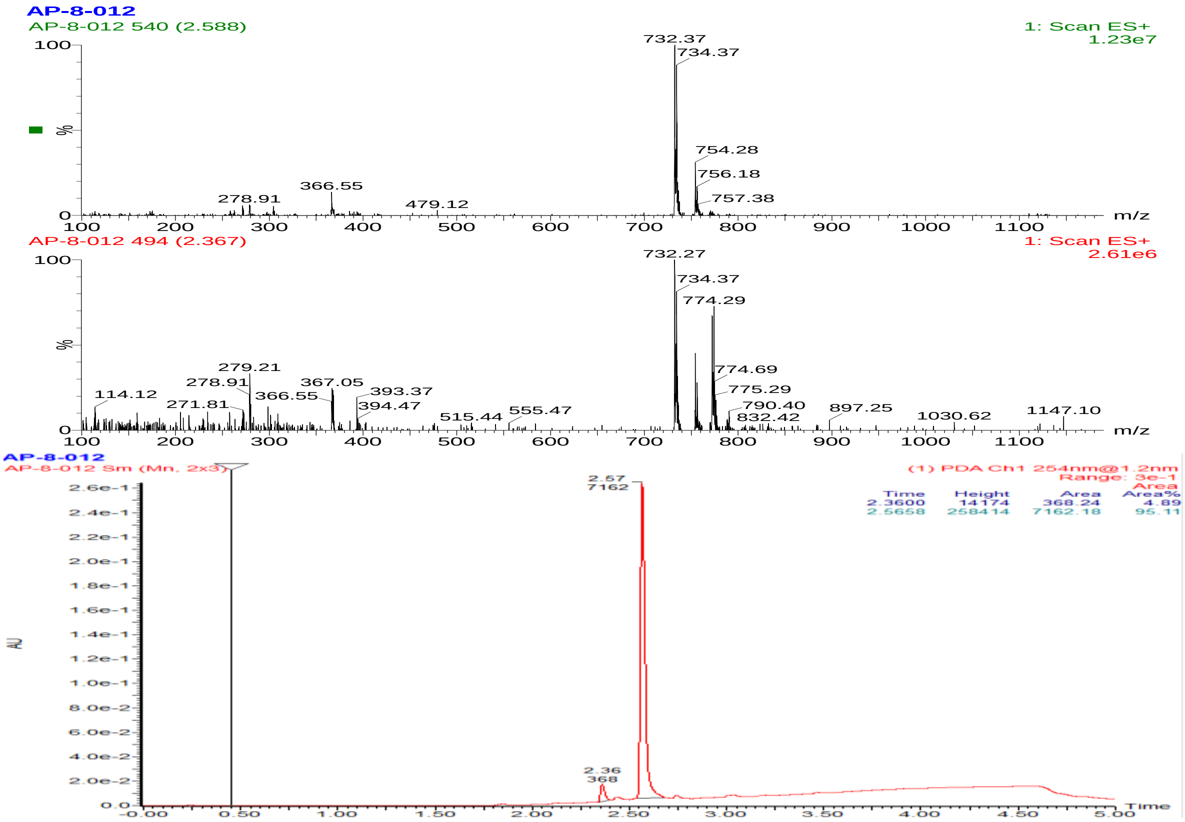

LCMS of Compound 20, AP-8-012

Figure S1: Synthesis of 21

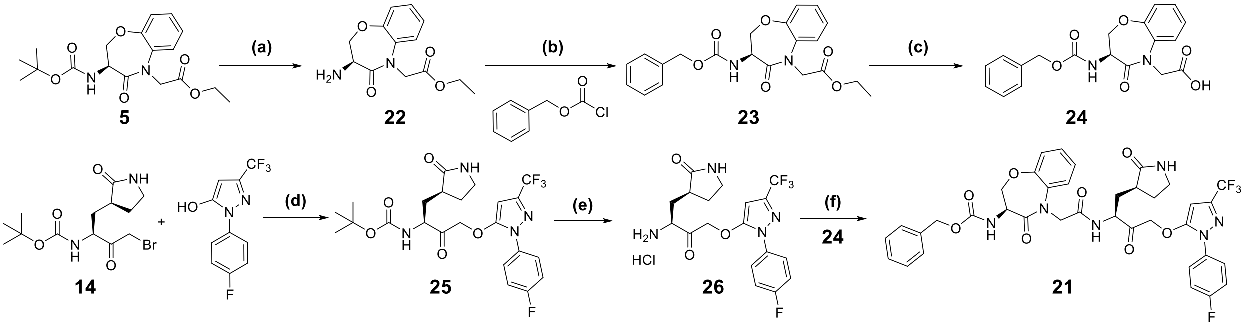

[a]**Reagents and conditions:** (a) 4N HCl in dioxane, DCM, 3 h. (b) Cbz Chloride, Et_3N, DCM, 0 °C - rt, 8 h. (c) 20 % aq NaOH in MeOH: THF: H_2O (5:5:1), rt, 4 h. (d) KF in anhyd. DMF, rt to 65 °C, 5 h. (e) 4N HCl in dioxane, DCM, 0 °C - rt, 2 h (f) 50 % T_3P in DMF, DIPEA, 0 °C - rt, 1 h.

Scheme S1 Experimental procedures.

(*S*)-Ethyl 2-(3-amino-4-oxo-3,4-dihydrobenzo[*b*][1,4]oxazepin-5(2*H*)-yl)acetate sulfate hydrochloride (22, NN-02-210); To a stirred solution of ethyl (*S*)-2-(3-((tert-butoxycarbonyl)amino)-4-oxo-3,4-dihydrobenzo[*b*][1,4]oxazepin-5(2*H*)-yl)acetate (400 mg; 1.09

mmol) in DCM was added 4N HCl in dioxane at 0 °C. The reaction mixture was stirred at room temperature for 3 hours, and completion of the reaction was confirmed by LC−MS. Volatiles were evaporated under reduced pressure and the crude product was co-distilled with dry toluene, and dried under high vacuum to afford the title compound (330 mg; 1.09 mmol, 100%) as a white solid. ^{1}H NMR (400 MHz, DMSO) δ 7.95 (s, 2H), 7.42 – 7.40 (m, 1H), 7.34 – 7.26 (m, 3H), 4.74 – 4.69 (m, 1H), 4.51 – 4.50 (m, 2H), 4.44 – 4.33 (m, 2H), 4.14 (q, J = 7.1 Hz, 2H), 1.20 (t, J = 7.1 Hz, 3H). ^{13}C NMR (100 MHz, DMSO) δ 168.8, 168.2, 150.0, 135.1, 128.4, 126.6, 124.0, 123.1, 75.6, 61.7, 50.2, 49.9, 15.6.
ESI-HRMS (m/z): calculated for $C_{13}H_{17}N_2O_4$ $(M+H)^+$ = 265.1188 found: 265.1181.

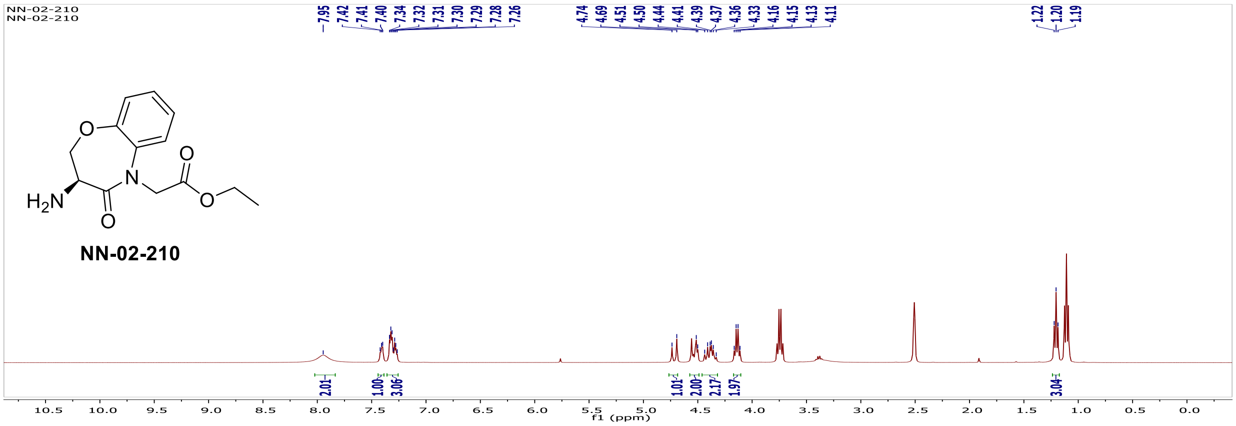

^{1}H NMR of Compound 22, NN-02-210

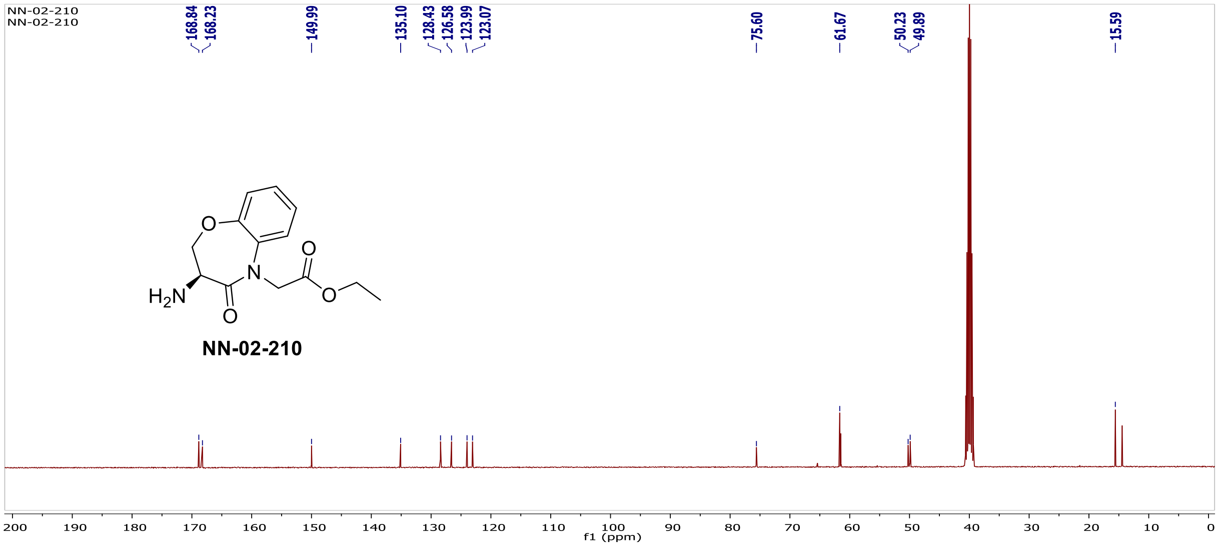

^{13}C NMR of Compound 22, NN-02-210

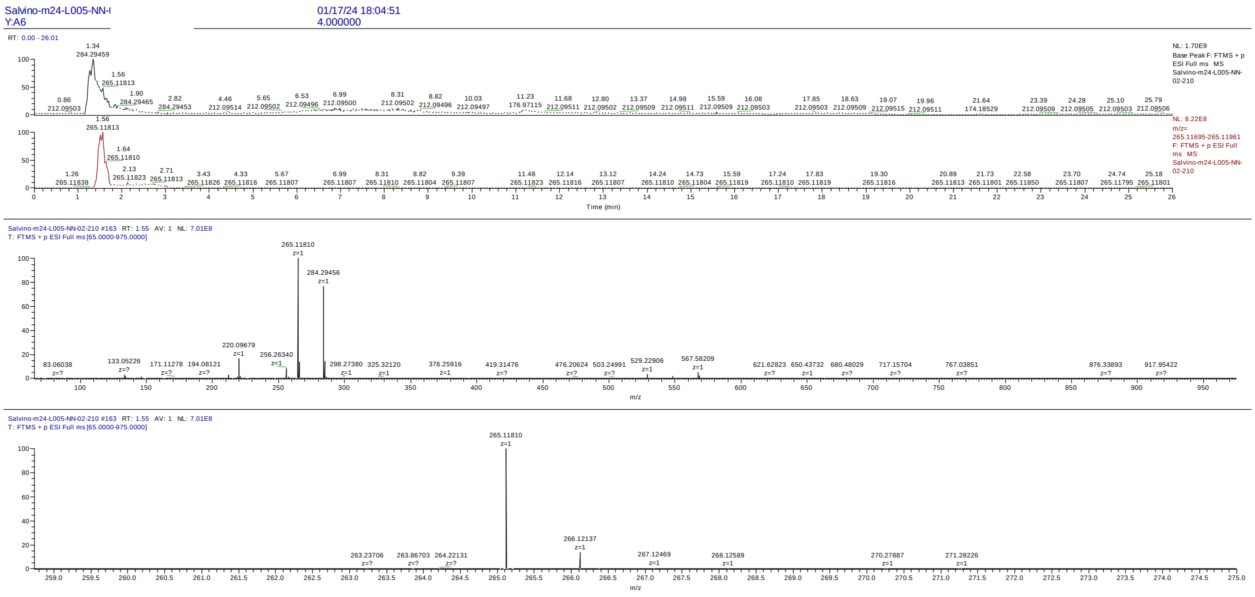

HRMS of Compound 22, NN-02-210

Ethyl (*S*)-2-(3-(((benzyloxy)carbonyl)amino)-4-oxo-3,4-dihydrobenzo[*b*][1,4]oxazepin-5(2*H*)-yl)acetate (23, NN-02-211); To a stirred solution of (*S*)-ethyl 2-(3-amino-4-oxo-3,4-dihydrobenzo[*b*][1,4]oxazepin-5(2*H*)-yl)acetate (NN-02-210) (300 mg; 0.997 mmol) in 10 mL of dry CH_2Cl_2 at 0 °C was added triethylamine (0.55 mL; 3.998 mmol) and benzyl chloroformate (255.0 mg, 1.49 mmol) dropwise simultaneously. The reaction mixture was allowed to stir for 8 hours at room temperature. Completion of the reaction was confirmed by LC−MS. The reaction mixture was quenched with cold water and the product was extracted with CH_2Cl_2 and dried over anhydrous Na_2SO_4. The solvent was evaporated under reduced pressure and the crude product was purified by flash column chromatography to afford the title compound (298 mg, 0.74 mmol, 75%) as a liquid. ^{1}H NMR (400 MHz, DMSO) δ 7.75 (d, J = 8.9 Hz, 1H), 7.41 – 7.27 (m, 8H), 7.24 – 7.20 (m, 1H), 5.00 (s, 2H), 4.64 – 4.60 (m, 1H), 4.55 – 4.47 (m, 2H), 4.40 – 4.30 (m, 2H), 4.14 – 4.07 (m, 2H), 1.17 (t, J = 7.1 Hz, 3H). ^{13}C NMR (100 MHz, DMSO) δ 170.3, 169.0, 156.2, 150.1, 137.1, 136.1, 128.8, 128.4, 128.3, 127.9, 126.2, 123.6, 123.0, 76.2, 66.3, 61.2, 51.3, 50.1, 14.4. ESI-HRMS (m/z): calculated for $C_{21}H_{23}N_2O_6$ $(M+H)^+$ = 399.1556 found: 399.1546.

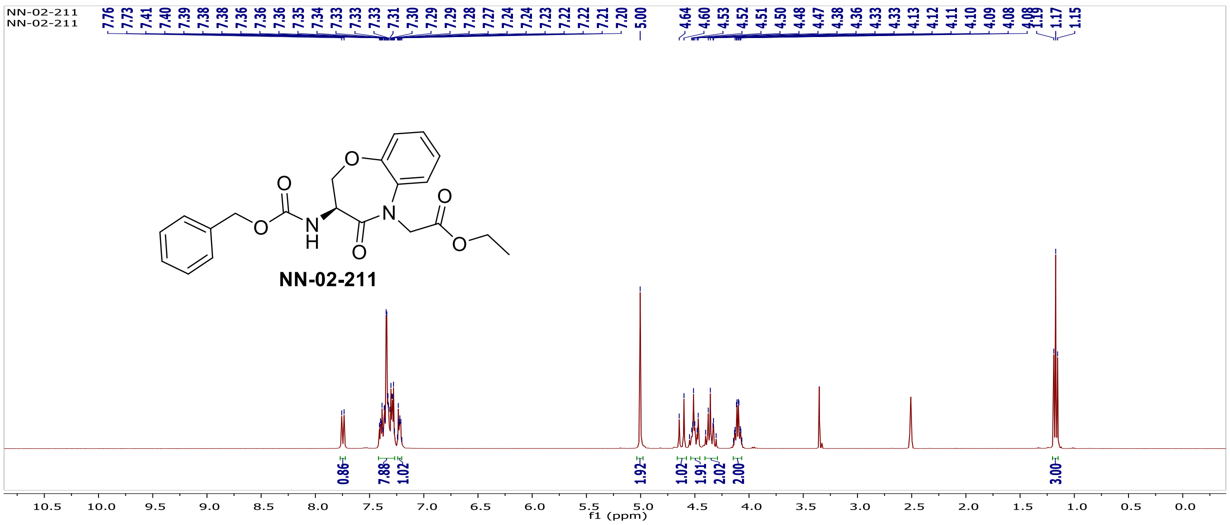

^{1}H NMR of Compound 23, NN-02-211

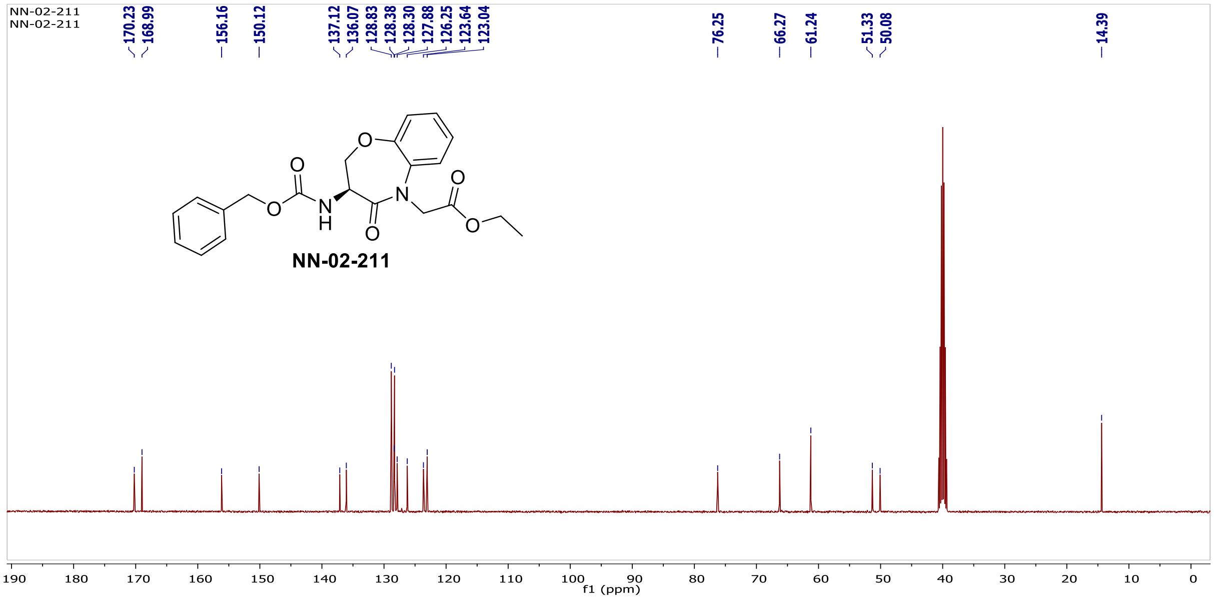

^{13}C NMR of Compound 23, NN-02-211

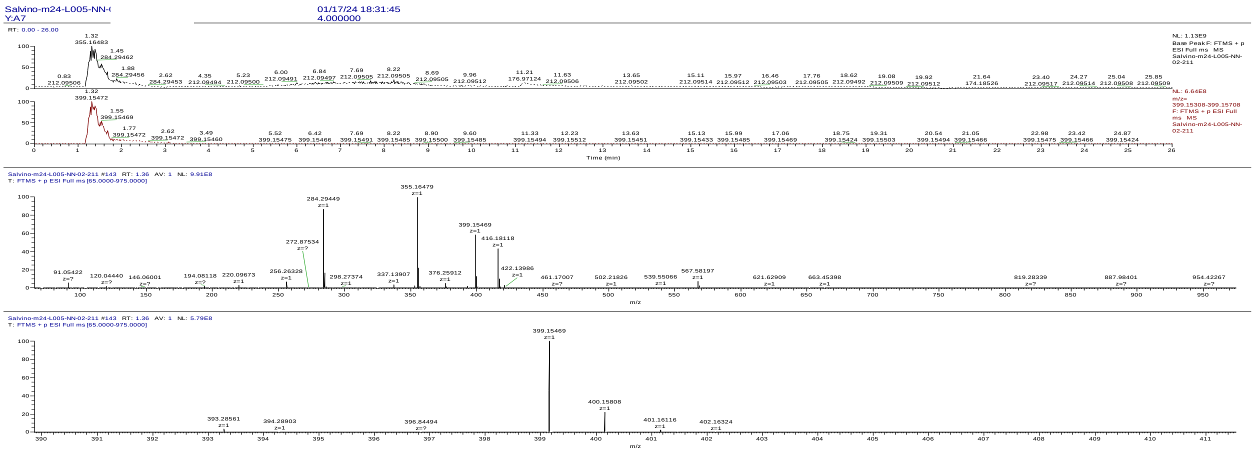

HRMS of Compound 23, NN-02-211

(*S*)-2-(3-(((Benzyloxy)carbonyl)amino)-4-oxo-3,4-dihydrobenzo[*b*][1,4]oxazepin-5(2*H*)-yl)acetic acid (24, NN-02-212); To a stirred solution of (*S*)-ethyl 2-(3-(benzyloxycarbonylamino)-4-oxo-3,4-dihydrobenzo[*b*][1,4]oxazepin-5(2*H*)-yl)acetate (NN-02-211) (50 mg; 0.125 mmol) in 10 mL of MeOH, H_2O and THF (5:5:1) at room temperature was added NaOH (20 mg; 0.502 mmol). The reaction mixture was stirred for 4 hours. Completion of the reaction was confirmed by LCMS. Volatiles were evaporated by reduced pressure, and the crude product was acidified with 1 N HCl at 0 °C to obtain a white precipitate which was filtered and dried under a high vacuum to afford the title compound (44 mg; 0.118 mmol, 95%) as a white solid. ^{1}H NMR (400 MHz, DMSO) δ 7.54 (d, J = 8.8 Hz, 1H), 7.43 – 7.40 (m, 1H), 7.30 – 7.22 (m, 5H), 7.18 – 7.13 (m, 2H), 7.10 – 7.08 (m, 1H), 4.91 (s, 2H), 4.48 – 4.41 (m, 2H), 4.28 – 4.16 (m, 2H), 3.76 – 3.72 (m, 1H). ^{13}C NMR (100 MHz, DMSO) δ 169.2, 156.0, 149.9, 137.5, 137.2, 128.8, 128.4, 128.3, 127.1, 125.9, 123.9, 122.6, 76.6, 66.2, 52.4, 51.2.

ESI-HRMS (m/z): calculated for $C_{19}H_{19}N_2O_6$ $(M+H)^+$ = 371.1243 found: 371.1234

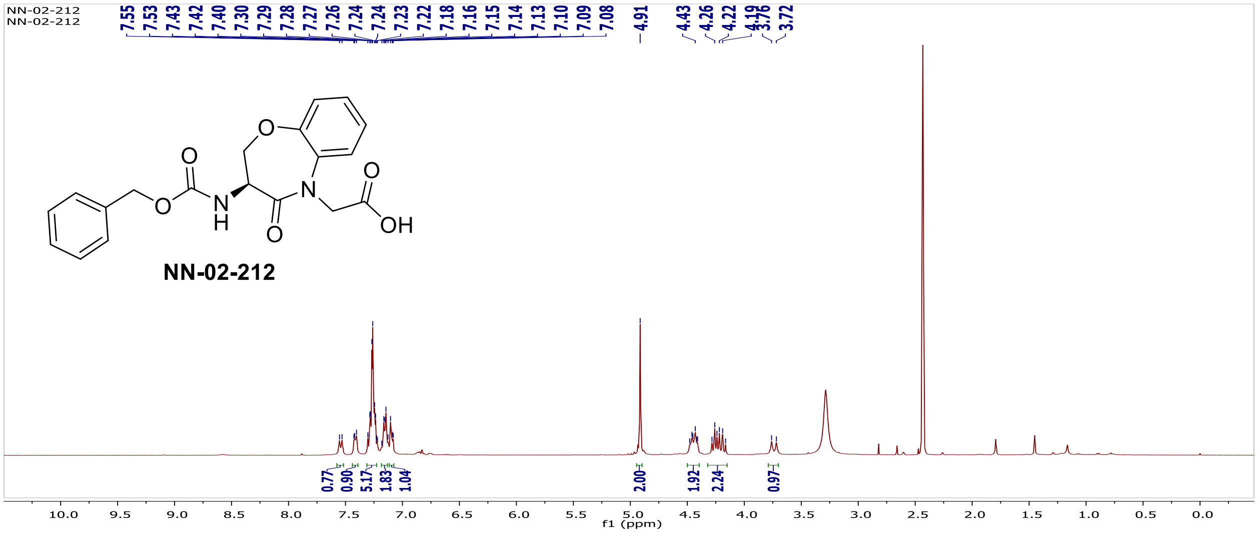

^{1}H NMR of Compound 24, NN-02-212

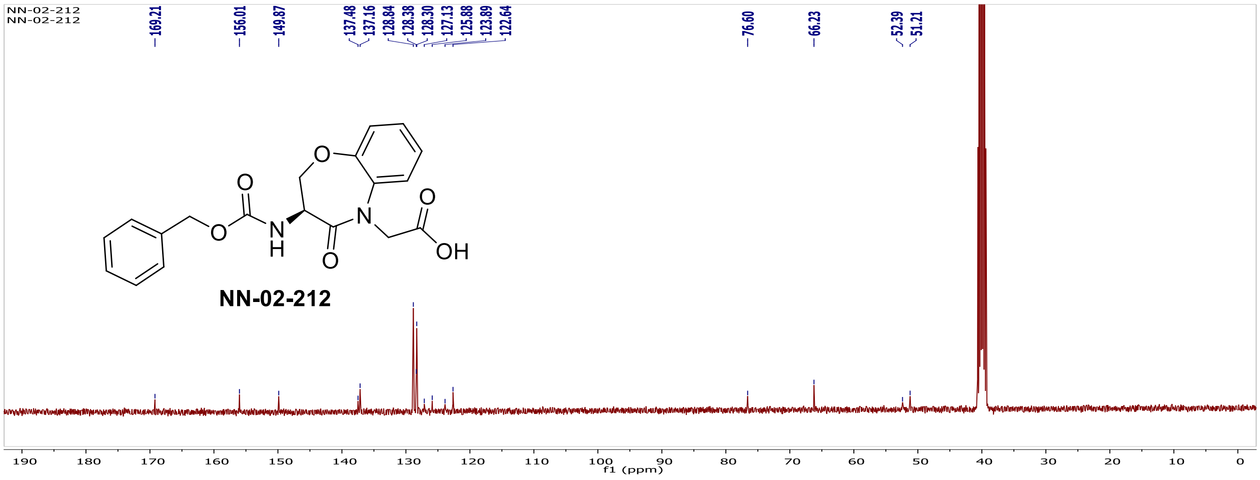

^{13}C NMR of Compound 24, NN-02-212

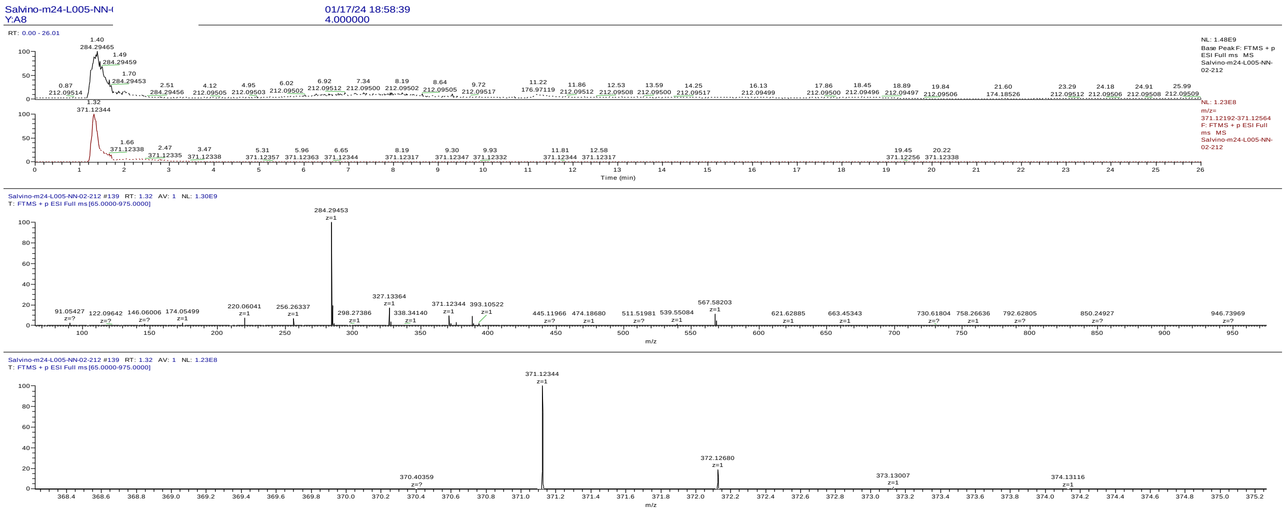

HRMS of Compound 24, NN-02-212

Tert-butyl (*S*)-4-(1-(4-fluorophenyl)-3-(trifluoromethyl)-1*H*-pyrazol-5-yloxy)-3-oxo-1-((*S*)-2-oxopyrrolidin-3-yl)butan-2-ylcarbamate (25, AP-7-279); To a stirred solution of tert-butyl (*S*)-4-bromo-3-oxo-1-((*S*)-2-oxopyrrolidin-3-yl)butan-2-ylcarbamate (**14**; AP-7-276 (AP-08-277); 517 mg, 1.48 mmol) and 1-(4-fluorophenyl)-3-(trifluoromethyl)-1*H*-pyrazol-5-ol (364 mg, 1.48 mmol) in 15 mL anhydrous DMF at rt, anhydrous potassium fluoride (215 mg, 3.7 mmol) was added and reaction temperature raised to 65 ^{o}C and stirred for 5 h under nitrogen atmosphere. Completion of the reaction confirmed by LC-MS, the reaction was cooled to rt, diluted with water (100 mL), and with ethyl acetate (30 mL), washed once with brine (15 mL), dried over Na_2SO_4, filtered, and concentrated under reduced pressure and the crude product was purified by flash column chromatography to afford title compound (609 mg; 1.18 mmol, 80%). ^{1}H NMR (400 MHz, DMSO) δ 7.78 – 7.75 (m, 2H), 7.69 – 7.61 (m, 2H), 7.42 – 7.38 (m, 2H), 6.37 (s, 1H), 5.27 (s, 2H), 4.21 – 4.15 (m, 1H), 3.17 – 3.13 (m, 2H), 2.29 – 2.23 (m, 1H), 2.19 – 2.15 (m, 1H), 1.94 – 1.87 (m, 1H), 1.68 – 1.60 (m, 2H), 1.41 (s, 9H). ^{13}C NMR (100 MHz, DMSO) δ 204.1, 178.7, 178.7, 161.4 (d, $J_{C\text{-}F}$= 243.8 Hz), 156.4, 154.8, 140.8 (q, $J_{C\text{-}F}$= 37.6 Hz), 133.9 (d, $J_{C\text{-}F}$= 2.9 Hz), 125.7 (d, $J_{C\text{-}F}$= 8.8 Hz), 121.5 (q, $J_{C\text{-}F}$= 267.1 Hz), 116.6 (d, $J_{C\text{-}F}$= 22.9 Hz), 86.5, 79.3, 75.0, 55.8, 38.0, 31.0, 28.5, 27.8.

ESI-HRMS (m/z): calculated for $C_{23}H_{27}F_4N_4O_5$ $(M+H)^+$ = 515.1918 found: 515.1909.

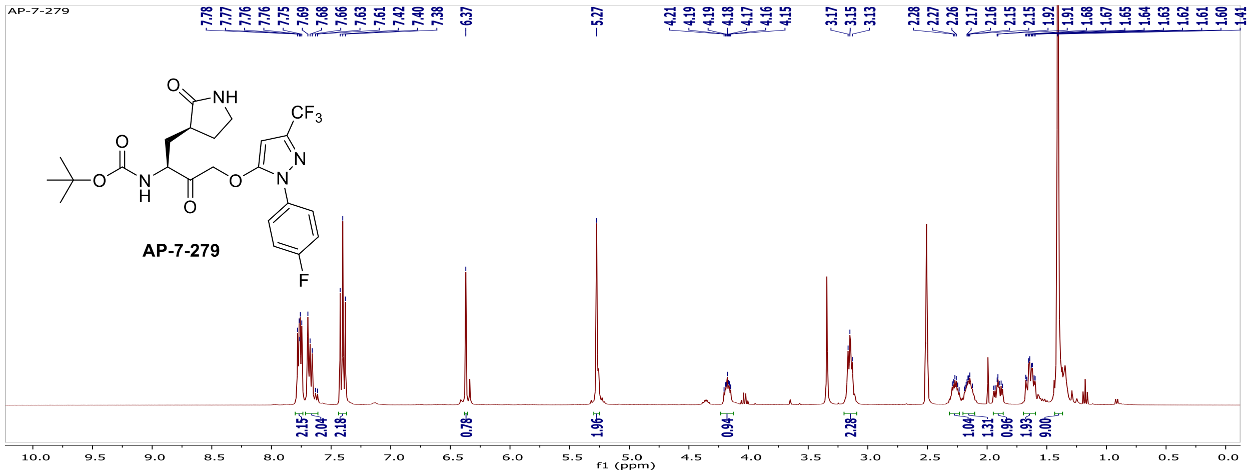

^{1}H NMR of Compound 25, AP-7-279

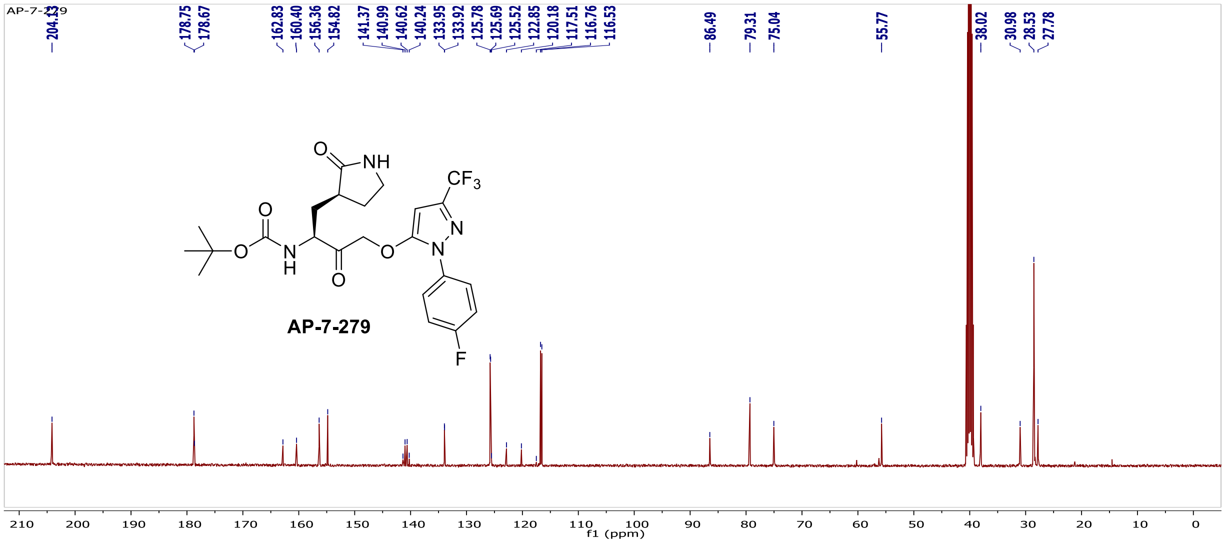

^{13}C NMR of Compound 25, AP-7-279

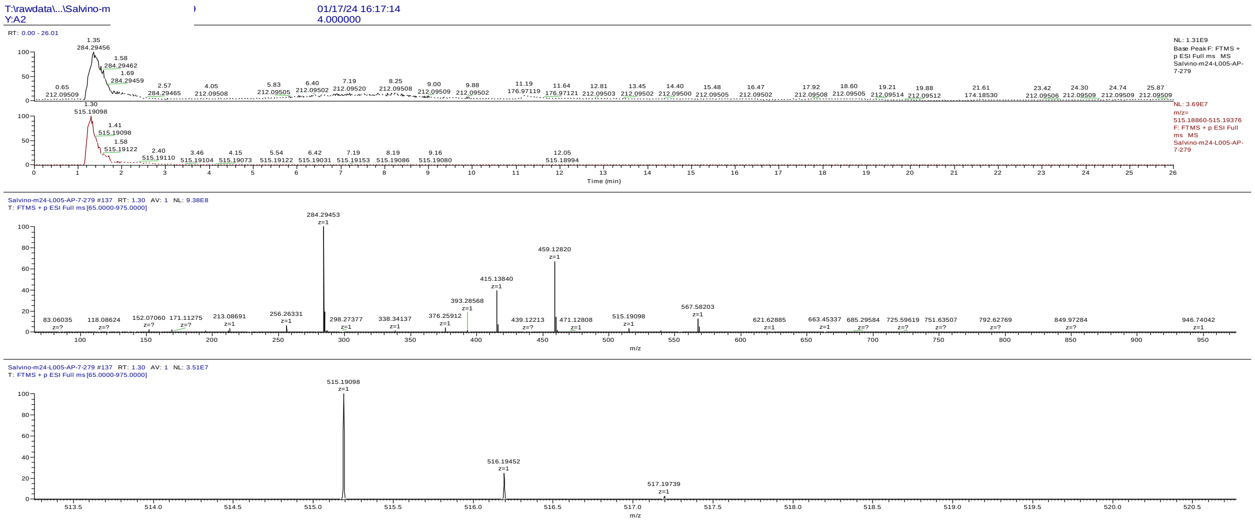

HRMS of Compound 25, AP-7-279

(*S*)-3-((*S*)-2-Amino-4-(1-(4-fluorophenyl)-3-(trifluoromethyl)-1*H*-pyrazol-5-yloxy)-3-oxobutyl)pyrrolidin-2-one hydrochloride (26, AP-7-279B (NN-02-208)); To a stirred solution of tert-butyl (*S*)-4-(1-(4-fluorophenyl)-3-(trifluoromethyl)-1*H*-pyrazol-5-yloxy)-3-oxo-1-((*S*)-2-oxopyrrolidin-3-yl)butan-2-ylcarbamate (50 mg, 0.097 mmol) in 8 mL of CH_2Cl_2 at 0 °C was added 4N HCl in dioxane. The reaction mixture was stirred at room temperature for 2 hours. Completion of the reaction was confirmed by LC− MS. Volatiles were evaporated under reduced pressure and the crude product was co-distilled with dry toluene, then dried under high vacuum to afford the title compound (44 mg; 0.097 mmol, 100%). ^{1}H NMR (400 MHz, DMSO) δ 8.64 (br. s, 2H), 8.01 (s, 1H), 7.78 – 7.74 (m, 2H), 7.41 (t, J = 8.8 Hz, 2H), 6.57 (s, 1H), 5.54 – 5.35 (m, 2H), 4.35 – 4.32 (m, 1H), 3.21- 3.17 (m, 2H), 2.60– 2.53 (m, 1H), 2.29 – 2.20 (m, 1H), 2.05 – 1.88 (m, 2H), 1.71 – 1.61 (m, 1H). ^{13}C NMR (100 MHz, DMSO) δ 200.8, 178.6, 161.7 (d, J_{C-F}= 244.0 Hz), 154.5, 140.8 (q, J_{C-F}= 37.0 Hz), 133.9 (d, J_{C-F}= 2.0 Hz), 125.8 (d, J_{C-F}= 8.1 Hz), 121.5 (q, J_{C-F}= 267.1 Hz), 116.7 (d, J_{C-F}= 23.0 Hz), 87.0, 74.9, 55.0, 38.7, 31.4, 30.8, 28.0.
ESI-HRMS (m/z): calculated for $C_{18}H_{19}F_4N_4O_3$ $(M+H)^+$ = 415.1393 found: 415.1384.

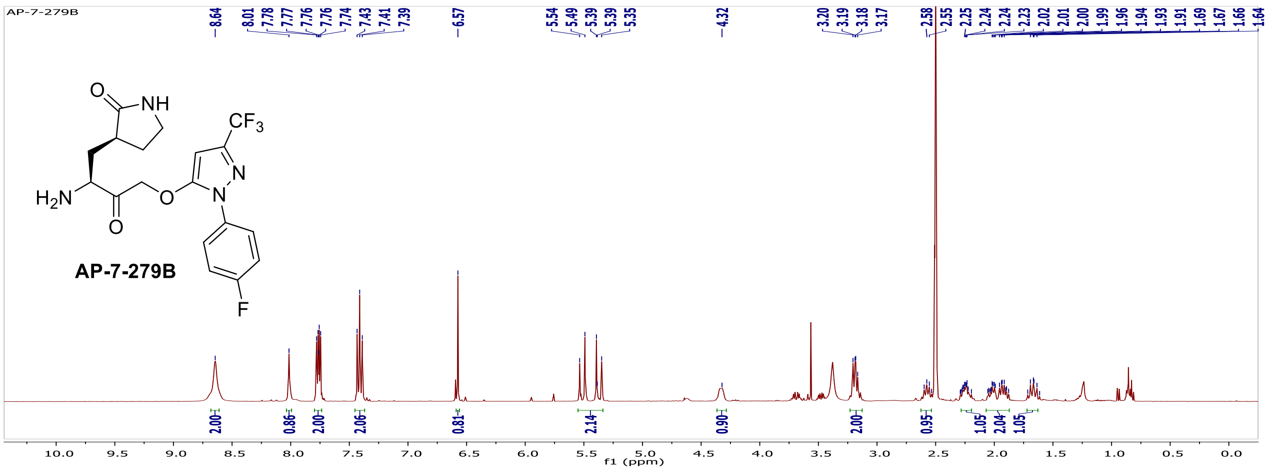

^{1}H NMR of Compound 26, AP-7-279B (NN-02-208)

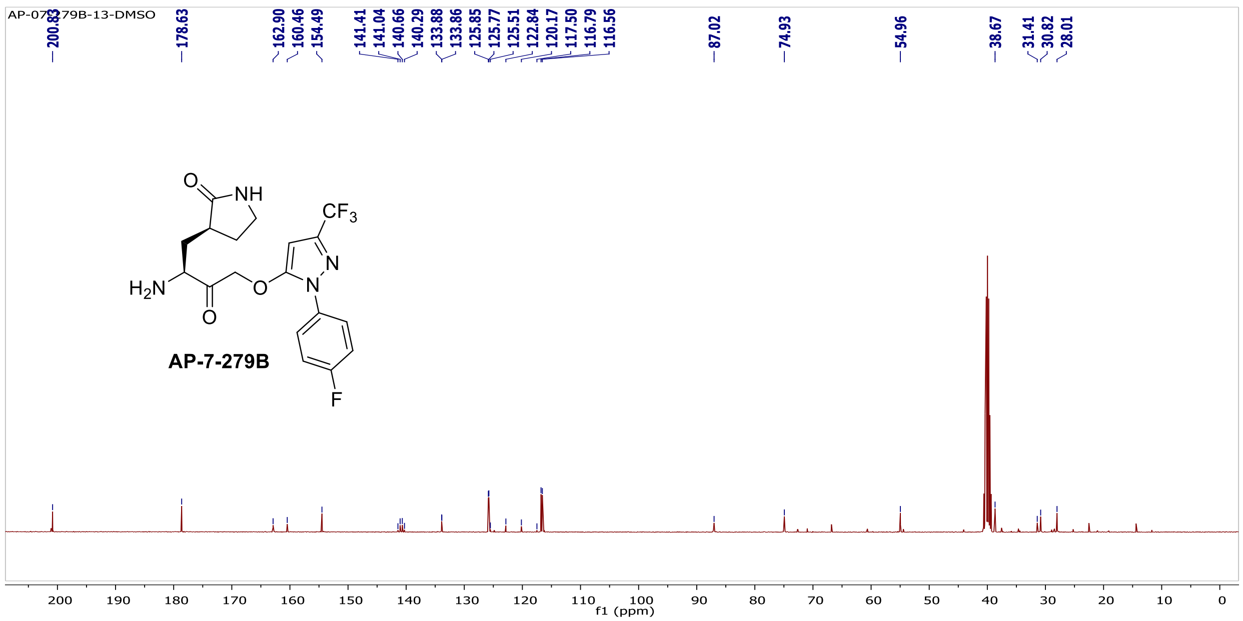

^{13}C NMR of Compound 26, AP-7-279B (NN-02-208)

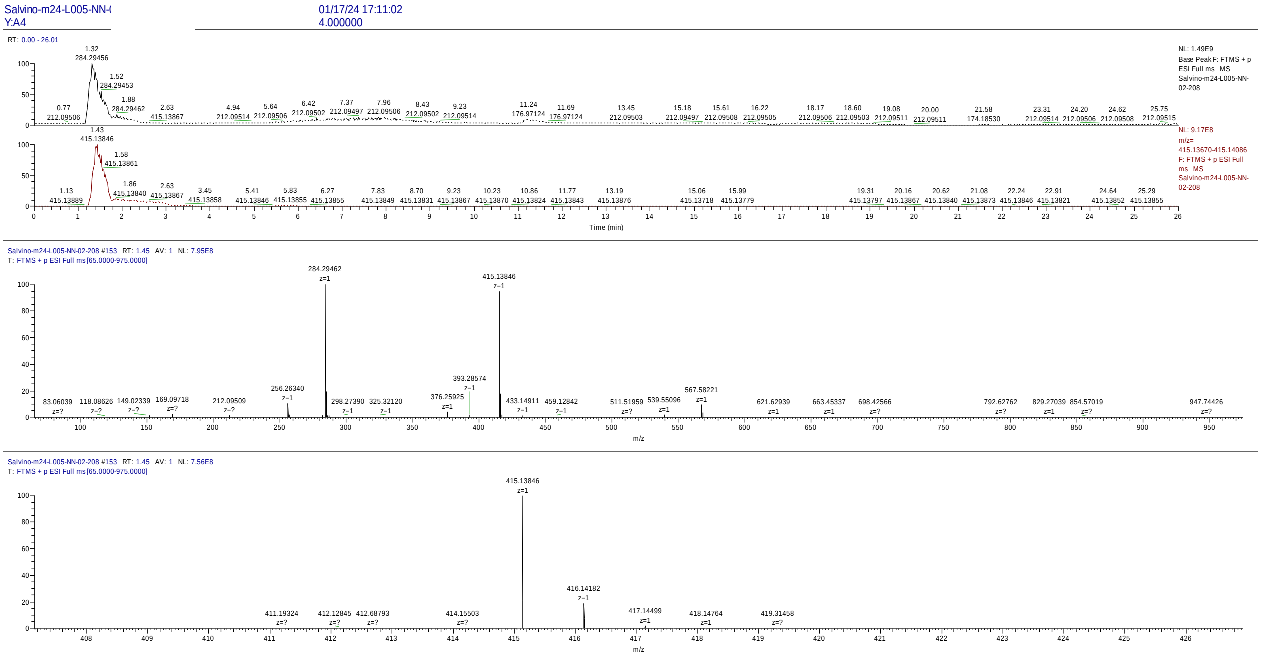

HRMS of Compound 26, AP-7-279B (NN-02-208)

Benzyl ((*S*)-5-(2-(((*S*)-4-((1-(4-fluorophenyl)-3-(trifluoromethyl)-1*H*-pyrazol-5-yl)oxy)-3-oxo-1-((*S*)-2-oxopyrrolidin-3-yl)butan-2-yl)amino)-2-oxoethyl)-4-oxo-2,3,4,5-tetrahydrobenzo[*b*][1,4]oxazepin-3-yl)carbamate (21, AP-8-014); To a stirred solution of ((*S*)-2-(3-(benzyloxycarbonylamino)-4-oxo-3,4-dihydrobenzo[*b*][1,4]oxazepin-5(2*H*)-yl)acetic acid (33 mg; 0.09 mmol) and (*S*)-3-((*S*)-2-amino-4-(1-(4-fluorophenyl)-3-(trifluoromethyl)-1*H*-pyrazol-5-yloxy)-3-oxobutyl)pyrrolidin-2-one hydrochloride (35 mg, 0.09 mmol) in 10 mL dry CH_2Cl_2 at 0 °C was added diisopropyl ethyl amine (46 mg, 0.36 mmol) and T3P 50% solution in

CH_2Cl_2 by weight (70 mg; 0.11 mmol) dropwise simultaneously. The reaction mixture was stirred for an additional 1 hour at 0 °C, and completion of the reaction was confirmed by LC−MS. The reaction mixture was quenched with cold water and the product was extracted with CH_2Cl_2 and dried over anhydrous Na_2SO_4. The solvent was removed under reduced pressure to provide the crude reaction product, which was purified by flash column chromatography to afford the title compound as a white solid (45 mg, 0.06 mmol, 65%). ^{1}H NMR (400 MHz, DMSO) δ 8.74 – 8.67 (m, 1H), 7.70 – 7.65 (m, 2H), 7.61 – 7.57 (m, 2H), 7.39 – 7.29 (m, 3H), 7.26 – 7.17 (m, 6H), 7.14 – 7.11 (m, 1H), 6.93 – 6.88 (m, 1H), 6.32 – 6.25 (m, 1H), 5.21 – 5.02 (m, 2H), 4.92 – 4.80 (m, 2H), 4.52 – 4.20 (m, 5H), 3.08 – 3.02 (m, 2H), 2.26 – 2.04 (m, 2H), 1.96 – 1.89 (m, 1H), 1.62 – 1.44 (m, 3H). ^{13}C NMR (100 MHz, DMSO) δ 203.2, 178.7, 170.2, 169.1, 161.6 (d, $J_{C\text{-}F}$= 242.0 Hz), 156.1, 154.7, 150.1, 140.9 (q, $J_{C\text{-}F}$= 38.0 Hz), 137.1, 136.5, 133.9 (d, $J_{C\text{-}F}$= 3.0 Hz), 128.8, 128.3, 128.2, 127.7, 126.1, 125.6 (d, $J_{C\text{-}F}$= 9.0 Hz), 124.0, 122.9, 116.6 (d, $J_{C\text{-}F}$= 23 Hz), 86.5, 85.2, 76.3, 74.7, 66.2, 54.6, 51.4, 37.6, 36.2, 31.2, 30.9. ^{19}F NMR (376 MHz, DMSO) δ 113.7 (s, 1F), 61.8 (s, CF_3),

ESI-HRMS (m/z): calculated for $C_{37}H_{35}F_4N_6O_8$ $(M+H)^+$ = 767.2452 found: 767.2445. ESI- LCMS (m/z): 767.39 $(M+H)^+$.

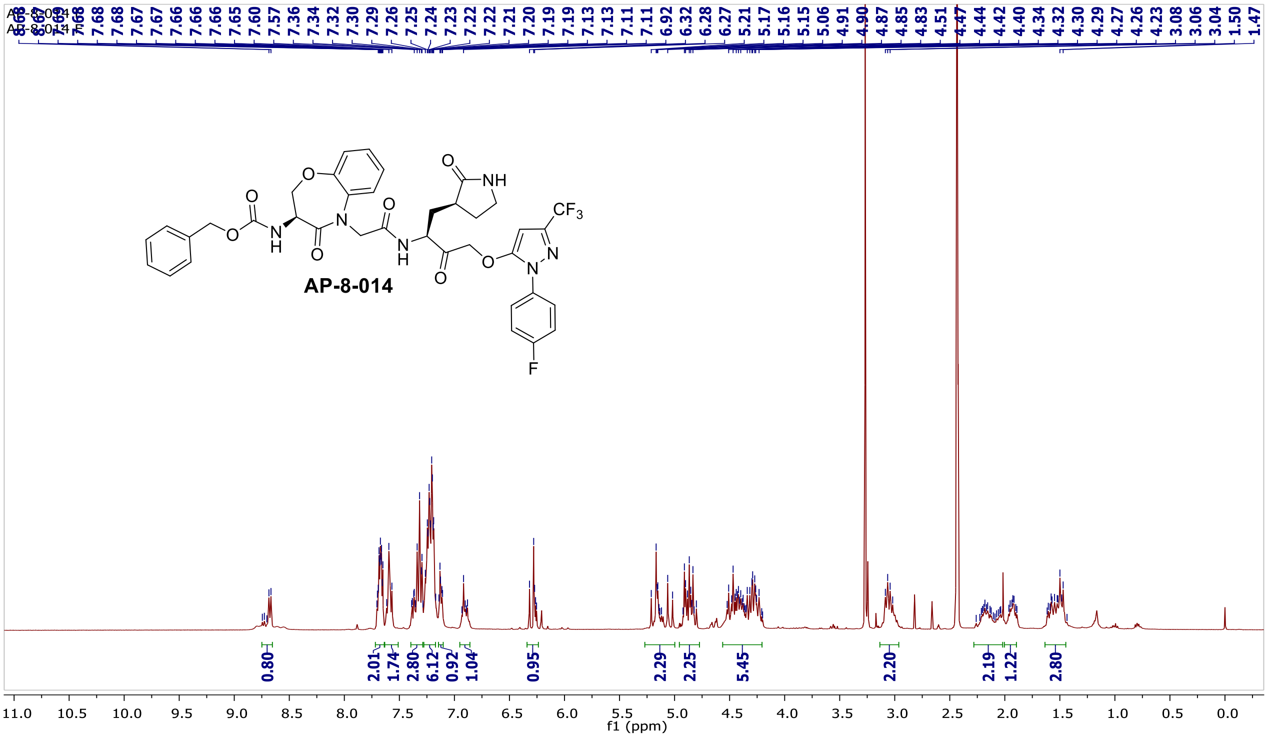

^{1}H NMR of Compound 21, AP-8-014

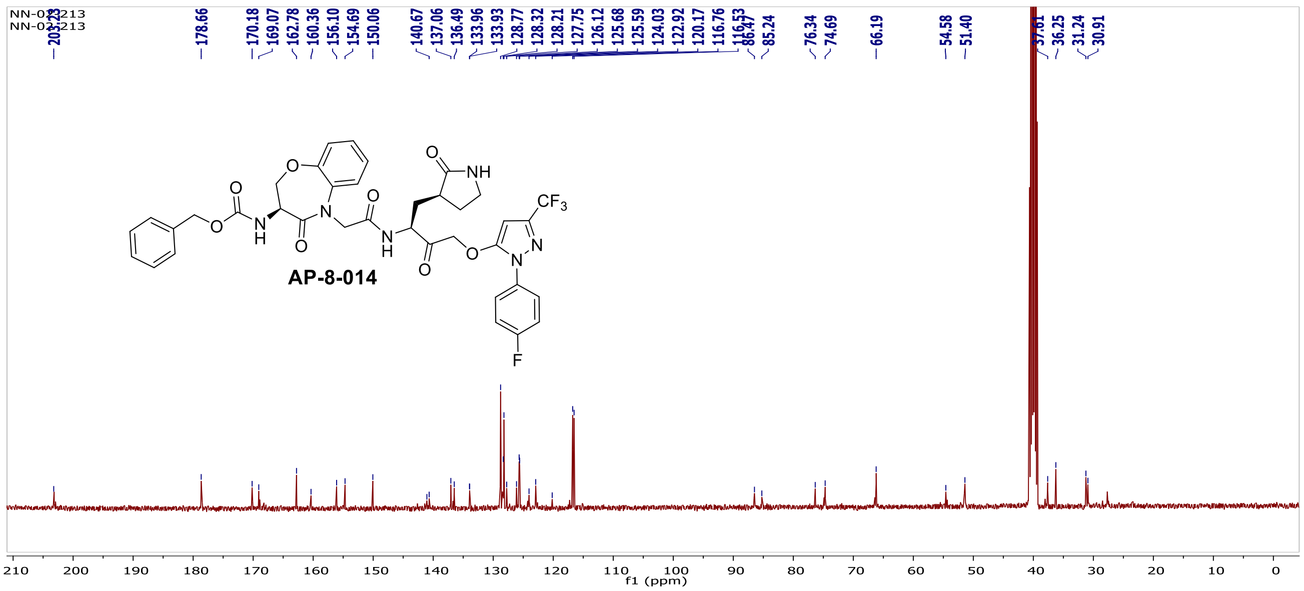

^{13}CNMR of Compound 21, AP-8-014

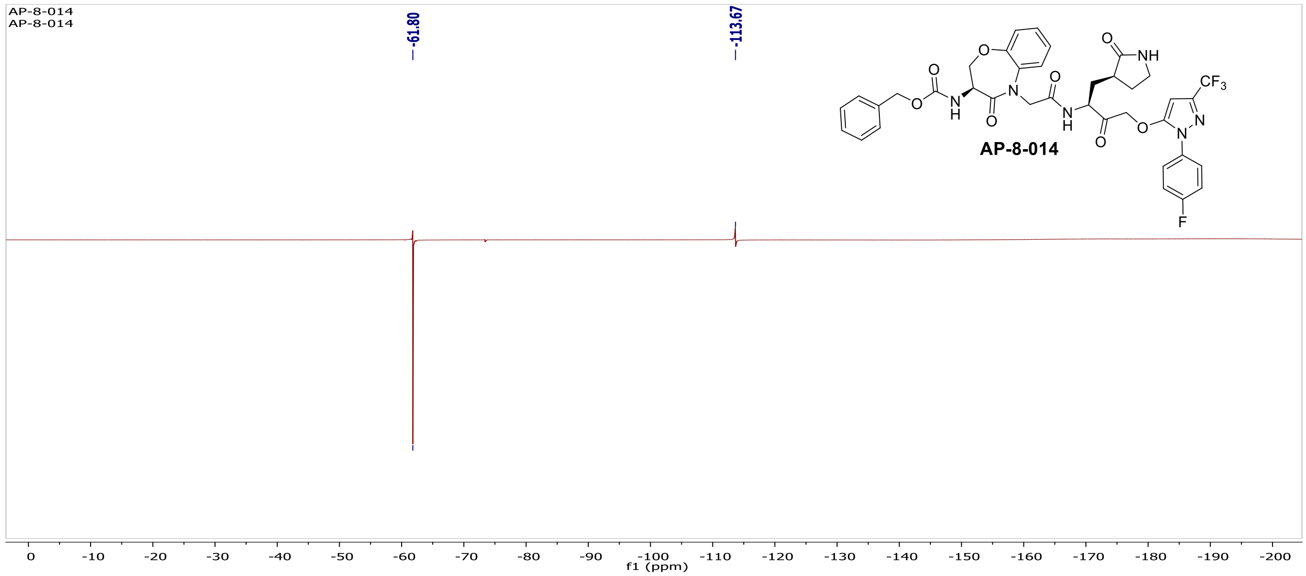

^{19}F NMR of Compound 21, AP-8-014

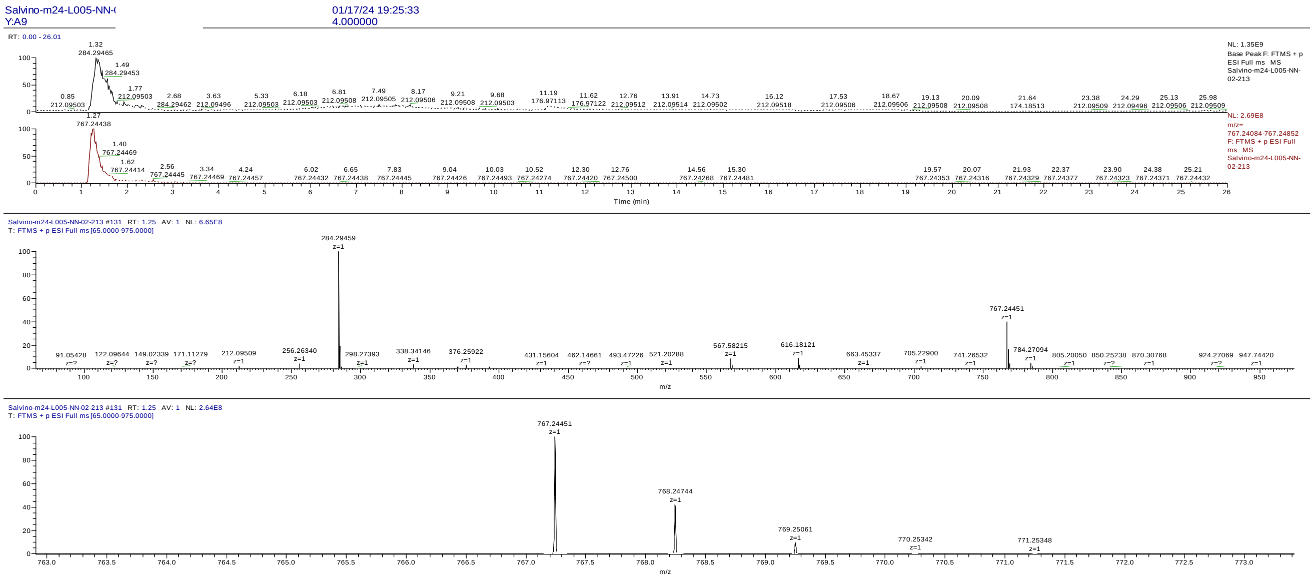

HRMS of Compound 21, AP-8-014

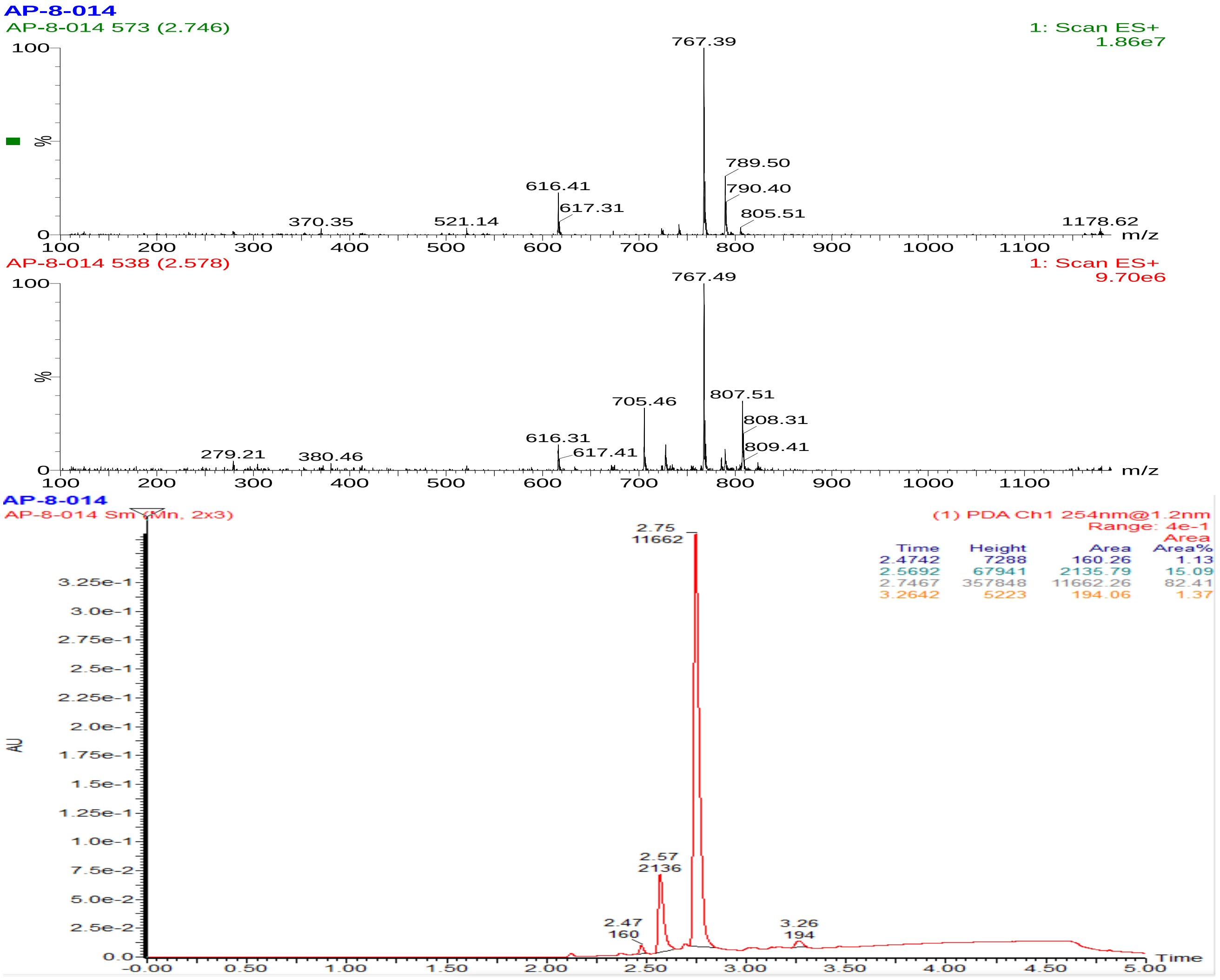

LCMS of Compound 21, AP-8-014
